# Protocol for Outcome Evaluation of Ayahuasca-Assisted Addiction Treatment: The Case of Takiwasi Center

**DOI:** 10.3389/fphar.2021.659644

**Published:** 2021-05-19

**Authors:** Brian Rush, Olivia Marcus, Sara García, Anja Loizaga-Velder, Gabriel Loewinger, Ariane Spitalier, Fernando Mendive

**Affiliations:** ^1^ Institute for Mental Health Policy Research, Centre for Addiction and Mental Health, Toronto, ON, Canada; ^2^ Department of Anthropology, University of Connecticut, Storrs, CT, United States; ^3^ Takiwasi Center for Rehabilitation of Drug Addicts and Research on Traditional Medicine, Tarapoto, Peru; ^4^ Nierika Institute, National Autonomous University of Mexico, Mexico City, Mexico; ^5^ School of Public Health, Harvard University, Boston, MA, United States; ^6^ Autonomous University of Barcelona, Barcelona, Spain

**Keywords:** ayahuasca, substance use, treatment, outcome, traditional medicine

## Abstract

The present study describes the protocol for the Ayahuasca Treatment Outcome Project (ATOP) with a special focus on the evaluation of addiction treatment services provided through Takiwasi Center, the first ATOP study site. The goal of the project is to assess treatment outcomes and understand the therapeutic mechanisms of an Ayahuasca-assisted, integrative treatment model for addiction rehabilitation in the Peruvian Amazon. The proposed intervention protocol highlights the significance of treatment setting in the design, delivery, and efficacy of an addiction rehabilitation program that involves the potent psychedelic tea known as Ayahuasca. After describing the context of the study, we put forth details about our mixed-methods approach to data collection and analysis, with which we seek to gain an understanding of why, how, and for whom this specific ayahuasca-assisted treatment program is effective across a range of outcomes. The ATOP protocol employs qualitative research methods as a means to determine which aspects of the setting are meaningful to clients and practitioners, and how this may correlate with outcome measures. This paper delineates the core principles, methods, and measures of the overall ATOP umbrella, then discusses the role of ATOP in the context of the literature on long-term residential programs. To conclude, we discuss the strengths and limitations of the protocol and the intended future of the project.

## Introduction

International research remains focused on finding effective interventions for substance use and other mental disorders that are appropriate for the social contexts in which they are implemented. Due to the limitations of mainstream addiction medicine and mental health treatments, there is growing interest in interventions based on traditional and complementary medicine, which is widely used by a majority of the global population and supported by international health initiatives (Sarris et al., 2014; Zhang et al., 2019). Given the tremendous burden of mental health globally (Vigo et al., 2016) and the limited coverage of mental health services, including substance use services, in most countries around the world (Kohn et al., 2004; Patel et al., 2010; Whiteford et al., 2013; Vigo et al., 2020), there is a global moral imperative to explore all promising alternatives, including ayahuasca-assisted treatment. The present paper describes the implementation and preliminary results of a research protocol designed as part of the Ayahuasca Treatment Outcomes Project (ATOP), with a goal to assess treatment outcomes and understand the therapeutic mechanisms of an ayahuasca-assisted, integrative treatment model in the Peruvian Amazon. Data collection commenced in 2016 as a mixed-methods longitudinal cohort study with long-term follow-up that will continue until at least through 2023. We report on the formation of the ATOP protocol, preliminary themes that have emerged in data analysis at baseline, and advocate for the strengths of a naturalistic mixed-methods investigation of a substance abuse treatment program that uses ayahuasca as part of its treatment model.

Ayahuasca is the Quechua-language name for a psychoactive plant beverage that originated in the Amazon region of several South American countries. For centuries, select indigenous and mestizo groups have considered ayahuasca a sacred medicine and one of several plant-based healing agents used by traditional healers (often referred to in Spanish as *curanderos*) (Gow, 1994; Luna, 1986; Anderson et al., 2012; Tupper, 2011). In the 20th Century, syncretic Christian churches that originated in Brazil, namely Santo Daime, Barquinha, and União do Vegetal (UDV), began using ayahuasca as a religious sacrament (Mercante, 2013; Dawson, 2017). The widespread popularity and eventual internationalization of Santo Daime, and to a lesser extent UDV, has been a significant factor in the spread of ayahuasca to many other parts of the world, in particular Europe, Australia, and North America. The internationalization of Peruvian mestizo shamanism and neo-shamanic practices also continue to influence the widespread growth of ayahuasca-related practices (Tupper, 2008; Labate and Jungaberle, 2011; Labate and Cavnar, 2014).

Ayahuasca is of growing interest to an international research community primarily for its putative therapeutic effects and its implications for global mental health, local and international drug policy, cross-cultural psychiatry, neuroscience, intellectual property law, indigenous rights, and intangible cultural heritage, to name just a few domains with keen stakeholders (see, for example, Labate and Cavnar, 2011; Tupper and Labate, 2014). Interest in its therapeutic potential is part of the resurgent research on the use of various hallucinogenic compounds (Tupper et al., 2015; Dakwar, 2016) for the treatment of substance use disorders (Garcia-Romeu et al., 2019; Nielson et al., 2018; Bogenshutz and Johnson, 2016:; Dakwar 2015; Bogenshutz et al., 2015; Ross, 2012; Bogenschutz and Pommy, 2012; Hendricks, 2014; Krebs and Johansen, 2012; Johnson et al., 2014) and other mental health challenges within the broad categories of depression and anxiety (Vollenweider and Kometer, 2010; Carhart–Harris et al., 2018a; Muttoni et al., 2019; Galvão-Coelho et al., 2021; Sarris et al., 2021), as well as eating disorders and protracted grief (González et al., 2017; Lafrance et al., 2017).

### Ayahuasca and Treatment for Substance Use Disorders

Preliminary evidence concerning the therapeutic effects of ayahuasca for alcohol and drug use disorders indicates treatment efficacy (Labate et al., 2010; Tupper, 2011; Mercante, 2013; Bouso and Riba, 2014; Dakwar 2015; Nunes et al., 2016). Based on the retrospective recall of lifetime alcohol and drug use, including severe substance use disorders, the results of observational studies among frequent ayahuasca users who are members of either the Santo Daime and/or the UDV churches in Brazil have consistently shown either complete remission in some cases or considerably reduced substance use in other cases (Grob et al., 1996; Fabregas et al., 2010; Halpern et al., 2008; Labate et al., 2010). A qualitative review of other ritual-therapeutic uses of ayahuasca also indicates efficacy for addressing problematic substance use including substance use disorders (Talin and Sanabria, 2017). Thomas et al. (2013) report positive outcomes on addiction-related measures in a follow-up assessment of members of an indigenous community participating in a structured ayahuasca retreat in Canada. Argento et al. 2019 conducted a qualitative analysis of interviews with participants in that same observational study. They reported reduced substance use and cravings as well as several factors underlying positive outcomes, including the awareness of negative thought patterns and behaviors not previously uncovered through conventional treatment and increased connection with self, others and nature/spirit. Other research based on qualitative interviews with people engaged with rural or urban-based ayahuasca-assisted treatment for substance use disorders in several countries and contexts also reports positive benefits that are attributed to multiple aspects of the therapeutic process, including mystical or “peak” experiences, insights, emotional processes and body-oriented effects (Harris and Gurel, 2012; Loizaga-Velder and Loizaga, 2014; Loizaga-Velder and Verres, 2014; Horak et al., 2018).

Alongside the growing literature on treatment effectiveness, toxicity and tolerability issues have also been investigated in human subjects. While more research is needed (Barbosa et al., 2012), the weight of the evidence suggests that occasional or long-term use of ayahuasca within well-structured ritual contexts carries little health risk and, importantly, no addictive potential (Grob et al., 1996; Riba et al., 2001; Fabregas et al., 2010; Bouso et al., 2012).

### Explanatory Mechanisms: Set and Setting Beyond Neuropharmacology

It is widely accepted that the underlying mechanisms through which ayahuasca-assisted treatment may exert its therapeutic effects are complex and multi-factorial. Psychological and neurobiological mechanisms of ayahuasca are similar to those induced by other psychedelics. While a full review of these potential mechanisms is beyond the scope of this paper, sufficient evidence has accrued to yield plausible hypotheses about underlying psychological and brain mechanisms contributing to treatment efficacy for substance use and other mental disorders. From a neuropharmacological point of view, the complexity of these hypotheses arises in part through the typical inclusion of two plant species in the ayahuasca brew that together implicate a variety of mechanisms for direct and indirect actions on both dopaminergic and serotonergic systems. This would include the general effects of tryptamines (namely, N,N-dimethyltryptamine or DMT) contained in the leaves of *Psychotria viridis* (locally known as Chacruna) as well as the harmala alkaloids contained in the ayahuasca vine (*Banisteriopsis caapi*). Dominguez-Clave et al. 2016 as well as Tófoli and de Araujo, (2016) have reviewed the pharmacology and neuroscience of ayahuasca and Araujo et al. 2015 offers a summary of mechanisms underlying the hallucinogenic properties of tryptamines more broadly, including DMT.

Brierley and Davidson (2012) also reviewed the implicated neurobiological mechanisms, in particular those related to harmine pharmacology. Findings suggest that harmine can regulate aberrant dopamine reuptake rates. This may be relevant for treatment of substance use disorders as reduced dopamine levels in the mesolimbic pathway interfere with the synaptic plasticity associated with the development and maintenance of addictions. These findings complement an earlier contribution by Vollenweider and Kometer (2010) on neurobiological mechanisms underlying psychoactive substances in the same family of psychedelics (e.g., LSD, ketamine) that impact glutamate neuroplasticity and other mechanisms implicated in treatment of mood disorders. Dos Santos et al. 2016 further updated this literature through a systematic review of clinical trials published in the last 25 years on the anti-depressive, anxiolytic and anti-addictive therapeutic benefits of ayahuasca, psilocybin, and LSD, including a review of a wide range of potential mechanisms. These mechanisms range from commonly accepted pathways related to agonism of the serotonin 5-HT_1A/2A/2C_ receptors and related modulation of glutaminergic neurotransmission, as well as anti-inflammatory action (see also Galvão-Coelho et al., 2020). Animal studies suggest that the anti-addictive potential of harmine and harmaline appear to involve imidazoline, glutamate, and dopamine pathways. Research with animals has demonstrated that ayahuasca can reduce alcohol induced sensitization and inhibit early behavior associated with alcohol use disorder, with one study concluding that ayahuasca can revert long term effects of alcohol dependency without itself inducing dependency (Oliveira-Lima et al., 2015). Modulation of cortisol levels has also been implicated (Galvão et al., 2018) as well as modulation of serum brain-derived neurotrophic factor (BDNF), a neurotrophin associated with major depression (Almeida et al., 2019). Finally, mechanisms associated with neuroplasticity and neurogenesis have been implicated by several studies of this family of psychedelic compounds (e.g., Morales-Garcia et al., 2017; 2020).

Based on neuroimaging, comprehensive neuro-psychological theory cites the important role of the default mode network (DMN) in the so-called entropic brain hypothesis, which hypothesizes that under the influence of psychedelics the entropic state of brain activity is heightened, which enhances sensitivity to set and setting and perceptions of connectedness (Palhano-Fontes et al., 2015; Carhart-Harris et al., 2018b; Carhart-Harris et al., 2018c; Carhart-Harris and Friston, 2019; Pasquini et al., 2020). Important activity in brain regions involved in emotional processing and introspection is also implicated (Kometer et al., 2012; Kraehenmann et al., 2015), including the functions of the interoceptive system that modulates approach or avoidance to environmental stimuli. The interoceptive system, closely embedded in the insula region of the brain, seems to be particularly important in drug use disorders as it processes information about body state and external stimulation, makes a prediction about risk and benefit, and then launches a behavioral response to achieve the desired goal (Paulus et al., 2013). Several neuroimaging studies also suggest that the altered state of consciousness produced by ayahuasca (and psilocybin) creates a disruption or interruption of the repetitive, rigid, and pathological pattern of negative and compulsive thoughts present in mood, anxiety and substance use disorders, contributing to changes in perspective, values and behavior (see summary of this literature by dos Santos et al., 2016).

While it is important to more fully understand the underlying neuropharmacological mechanisms, they are only part of a complex interplay between the mechanisms related to the dose and quality of the substance ingested and non-pharmacological factors such as the individual characteristic of the person (*set*) and the therapeutic context of the experience itself (*setting*) (e.g., Loizaga-Velder and Loizaga, 2014; Loizaga-Velder and Verres, 2014; Uthaug et al., 2021). The interplay between set, setting, and neuropharmacology is an important consideration within psychedelic science that involves analysis of factors that influence the user’s general mindset such as motivation for use, expectation of effects, intentions, and preparation (set) and the social interactions, physical location, and wider sociocultural conditions (setting) (Hartogsohn, 2016). Citing the original ideas postulated by Carhart-Harris and Nutt (2017), Carhart-Harris et al. (2018b) articulate the hypothesized link between these individual and environmental factors and potential underlying neuropharmacological mechanisms, namely increased serotonin 2 A receptor signaling mediating cortical plasticity and an associated sensitivity to internal (i.e., endogenous processes and pre-existing mental context) and external influence (i.e., the environment), is proposed to be the key underlying mechanism.

The role of set and setting has historically been a topic of high interest in the exploration of therapeutic models that incorporate psychedelic substances, including ayahuasca (Metzner et al., 1965; Carhart-Harris et al., 2018c; Pollan, 2018). Among the first to advocate for going beyond the mechanistic aspects of the neurobiological hypotheses, Liester and Prickett (2012) and Prickett and Liester (2014) discuss the role of transcendent experiences with ayahuasca in the treatment of substance use disorders, putting forth a ‘psychological theory’ as well as a ‘transcendental theory.’ Also emphasizing the psychological aspects, Dominguez–Clave et al. (2016) reviewed research on psychological variables and reported that ayahuasca appears to enhance facets of mindfulness related to acceptance and the ability to take a detached view of one’s own thoughts and emotions. They conclude that ayahuasca shows promise as a therapeutic tool by enhancing self-acceptance and allowing safe exposure to emotional events, citing its potential usefulness in the treatment of impulse-related, personality, and substance use disorders and also in the handling of trauma. Franquesa et al. (2018) emphasize underpinning psychological variables related to “de-centering” values and self, while Timmermann et al. (2018) have focused on the phenomenological relationship between DMT-induced experiences of ego dissolution (often characterized by ‘ego death’) and near-death experiences (NDEs), the latter of which are known to confer long-term positive changes in well-being. Psychological *integration* of the ayahuasca experience, with psychotherapeutic processes such as group sharing, art therapy and/or professional psychosocial support and interpretation, are also noted to be particularly important for positive outcomes in ayahuasca-assisted treatment and rehabilitation for substance use disorders (Loizaga-Velder and Loizaga, 2014).

Among other key elements of the ayahuasca experience such as symbolic aspects related to purging in a non-ordinary state of consciousness, psychological insights and emotional processing of significant life events, the importance of the transcendental, or “peak”, mystical experience is emphasized as a strong mediating factor in treatment outcome for many ayahuasca users. Mystical experiences have been found to have and important therapeutic value also in research on the use of psychedelics for alleviating depression and anxiety in terminally ill cancer patients (Griffiths et al., 2016), and other therapeutic processes (Majic et al., 2015). The intensity of mystical-type experiences during psilocybin-assisted therapy has been correlated with treatment outcomes for alcohol dependence (Bogenschutz et al., 2015) and nicotine dependence (Garcia-Romeu et al., 2015; Johnson et al., 2014; 2017). In an effort to explain such results, Carhart-Harris et al. 2018b emphasize the different dimensions of “connectedness” that arises in the psychedelic experience (e.g., connected to nature, to others, or the larger Universe in a mystical way). Kjellgren et al. (2009) characterize the stages of the ayahuasca experience as often culminating in a deep transpersonal experience and fundamental changes in worldview, personal development, interests, and orientation to life in general. In a broad-based Internet-based survey, Griffiths et al. (2019) found that ayahuasca users report mystical experiences to be more positive and with more enduring impact on life satisfaction, social relationships, spiritual awareness in everyday life, and attitudes about life and self, mood and behavior compared to those using other psychedelics such as LSD, psilocybin, or DMT.

With respect to the importance of the therapeutic setting, Griffiths et al. (2019) speculate that the more enduring impact of the ayahuasca experience may be related to its common use in a structured religious or spiritual group context. Similarly, ethnographic work reported by Talin and Sanabria (2017) concerning substance use-related recovery experiences with ayahuasca highlight the importance of collective ritual-ceremonial healing spaces and practices for therapeutic outcomes, which points to the importance of the therapeutic context. A retrospective qualitative study of ayahuasca use for the treatment of eating disorders also illustrated the multiple dimensions of the experience that may contribute to efficacy, including the group ceremonial context (LaFrance et al., 2017). In the more general literature on substance use treatment outcomes, there is considerable evidence for the importance of the treatment environment, both structural (e.g., institutional vs. more community-oriented services (Urbanoski and Rush, 2013) and functional (e.g., the importance of treatment milieu; therapeutic alliance).

Some organizations in Brazil (Mercante, 2013) and other South American countries offer ayahuasca-assisted treatment for substance use disorders in various structured and semi-structured formats and organizational contexts (Politi et al., 2018). In Peru, Colombia, Ecuador and elsewhere, there are many centers in which curanderos (i.e., local healers) offer ayahuasca in ceremonial contexts that differ widely in practice (Holman 2011; Apud 2015; Marcus and Fotiou 2019). These centers cater largely to foreigners who report a range of motivations for seeking ayahuasca shamanism, from self-improvement, to spirituality-oriented and healing experiences, as well as healing for substance use disorders and other mental health issues (Winkelman, 2005; Kavenska and Simonova, 2015). An additional complex layer of ayahuasca use in this context is the phenomenon of ‘ayahuasca tourism,’ which engenders certain perceptions, expectations, and a particularly curated kind of esthetic and relational experience with ayahuasca shamanism. Both benefits and risks associated with ayahuasca tourism have been reported (Kavenska and Simonova, 2015; Prayag et al., 2016; Bauer, 2018).

### The Ayahuasca Treatment Outcome Project

ATOP was developed as a multi-site research project with sub-projects and teams in different countries. Each sub-project is required to secure its own individual research funding and is subject to unique timelines, as well as regulatory and cultural circumstances. The ATOP team initially anticipated project sites in Peru, Mexico, Brazil and *Argentina*. There is a potential for additional sites to be added, each required to share a set of common principles, methods and measures that will support the roll-up of key aspects of the resulting data. These common elements are referred to as the ATOP umbrella. In effect, ATOP aims to tell a story concerning the effectiveness of ayahuasca-assisted treatment for addiction–a story with separate chapters but a common story line. To this end, it was acknowledged from the outset that the success of ATOP would depend on the decisions made by a central team on the core features of the ATOP umbrella, as well as the commitment of the sub-projects to follow the core principles and protocols. That being said, the ATOP leadership team acknowledges that the traditions and cultural/legislative context in each country will dictate some deviation.

The key principles and elements of a common protocol were identified at a three-day workshop held in October 2013 in Tarapoto, Peru, hosted by the Takiwasi Center. Participants represented a variety of backgrounds and experience from different treatment centers, scientific disciplines, and therapeutic orientations, including traditional healing, to form a protocol in coherence with the intercultural nature of potential ATOP project sites and the modern ayahuasca phenomenon. The meeting was designed to build upon a strong foundation of previous scientific research as well as to foster collaborative initiatives and knowledge exchange between stakeholders from different perspectives.

As a result of these structured deliberations a core set of inclusion criteria for study sites, methodological approaches, and measures were identified to allow for synthesis of the research across multiple sites.

### Core Inclusion Criteria

• The focus of ATOP was defined as the evaluation of ayahuasca-assisted services for *addiction-related challenges*, which may also involve co-occurring mental health challenges such as depression and anxiety disorders, as well as other physical health challenges. It was also decided to include tobacco use disorders within project scope but to exclude “process addictions” such as sex addiction, gambling or video gaming.
• Program sites would be selected only from Latin American countries and chosen to represent a continuum of approaches that *integrate* traditional Amazonian plant medicine, including ayahuasca as a core element, with a range of western practices such as psychotherapy and psychosocial supports. One criterion for site selection that was hotly debated for the ATOP umbrella was that there would be no use of other psychoactive substances in the therapeutic context, including Kambo and cannabis, but excluding tobacco given its core use in Amazonian traditional medicine and in the neo-shamanic, naturalistic context as a cleansing and healing agent.
• Last, another key principle for the ATOP umbrella was an agreement on the study *exclusion criteria* for individual participants, including pregnancy and current use of MAO-inhibiting anti-depressants, and other contra-indicated medications.

### Core Methods and Measures

The following general aspects of a common study protocol were considered essential features of the ATOP study design. Each of these is described in more detail below.

1. Contextual Description of Treatment Settings.
  • Original and/or existing ethnographic description.
  • Interviews with program managers, staff, and healers.
2. Observational design with mixed methods
  • Baseline, discharge and periodic post-discharge measurement of treatment outcomes with validated, quantitative measures, with minimum of one-year post-discharge follow-up.
  • Scheduled semi-structured interviews with study participants for qualitative analysis.

At the present time, the Takiwasi Center remains the first and only active ATOP study site. The ATOP-Takiwasi study was initiated in January 2016 and this paper reports the protocol used in data collection and analysis for the mixed-methods program evaluation, including post-treatment follow-up. Preliminary results have been published on select mental health outcomes achieved during treatment (Giovannetti et al., 2020) and on samples of Takiwasi patients assessed with selected measures prior to implementation of the full protocol reported on here (O’Shaughnessy, 2017; Berlowitz et al., 2019). As described below, the research team is now poized to report on the first cohort of patients followed for a full 12 months after discharge. Negotiations continue in Mexico in order to meet importation and other regulatory requirements for implementing the ATOP protocol at Nierika Center.

### Justification for the Study

Among the plethora of options for ayahuasca-assisted healing now available in the Amazonian and global context, the Takiwasi Center, and other potential ATOP sites such as Nierika in Mexico, are unique in ways which make them of high interest from a treatment research perspective, especially the study of treatment outcomes for substance use disorders. Takiwasi, for example, is approaching 3 decades of practice in treating problematic substance use and substance use disorders. It is officially recognized by the Peruvian Ministry of Health as an integrative rehabilitation clinic that incorporates rigorous and frequent medical and psychological pre-treatment assessment, ongoing monitoring, and supervision. This clearly sets it apart from the diverse and unregulated ayahuasca centers that now proliferate in the Amazon basin, as well as the neo-shamanic and New Age ayahuasca groups in urban spaces throughout the world. Takiwasi provides a stable organizational environment and infrastructure for the conduct of a scientifically sound study of treatment outcome, particularly in its reasonably well-defined and documented assessment and treatment protocol. Client assessment protocols and other program documentation record potentially predictive client characteristics and also facilitate measurement of treatment exposure to help explain treatment outcomes. Further, given its longevity and organizational maturity, it is useful to study Takiwasi as a longstanding model for contextualized intercultural psychedelic-assisted therapy in light of clinical trials which are now attempting to (re)invent a “new” therapeutic paradigm. From the point of view of examining the complex interplay of set and setting in the determination of treatment outcome, this highly complex treatment protocol offers a rich, if not somewhat daunting, opportunity given its syncretic hybrid of Amazonian and Christian cosmologies and diverse techniques of traditional plant medicine, rituals, psychotherapeutic and self-help approaches offered within the treatment milieu of a therapeutic community. Since its inception, Takiwasi has been supportive of more than 70 research studies, mostly master’s and doctoral theses, across several disciplines including psychology/psychiatry, neuroscience, sociology, anthropology, musicology, and art. Researchers have investigated the inpatient population (Presser-Velder, 2000; Horák 2013; Cervi et al., 2019; Defelippe et al., 2019; Berlowitz et al., 2020; Giovannetti et al., 2020), seminar participants (Dupuis, 2018, Dupuis, 2021), and a mix of inpatients, seminar participants, and outpatient diet-retreat participants (Håland 2014; Marcus and Fotiou 2019). Although there are high rates of treatment non-completion (Defelippe et al., 2019), preliminary data are suggestive of short-term treatment efficacy (Giove Nakazawa, 2002; Mabit, 2002; Berlowitz et al., 2019; Giovannetti et al., 2020) and ethnographic inquiry has implicated the various aspects of the treatment context that may influence treatment outcomes (Bustos, 2006; O’Shaughnessy, 2017; Marcus and Fotiou, 2019).

### Treatment for Substance Use Disorders and Long-Term Residential Programs

The interest in studying treatment outcomes at the Takiwasi Center extends beyond the specifics of its treatment model for ayahuasca-assisted healing, to include the potential contribution to research on long-term residential treatment models, and their place in the overall continuum of services for substance use treatment. Modern conceptualizations of substance use treatment systems articulate a place for residential treatment options, including long-term options that extend well beyond the more common 4–6-weeks treatment models (World Health Organization, 2016). That being said, residential treatment services must be viewed within a “stepped-care” model whereby they are most effectively and efficiently utilized after other less intensive options (i.e., one might need to “step up” to this option). Alternatively, in a “stepped-down” approach, residential treatment may include a post-discharge transition to outpatient treatment as part of a well-defined continuing care plan (Breslin et al., 1998; Bower and Gilbody, 2005; Rush and Urbanoski, 2019). These considerations also speak to the need to allocate scarce resources for substance use treatment wisely, recognizing that the effectiveness of residential treatment has been judged to be “moderate” at best (Martin and Rehm, 2012; Reif et al., 2014). Outcomes are also highly variable with respect to therapeutic communities specifically, with more positive outcomes reported by reviews focused on recovery-oriented outcomes such as legal and employment status and psychosocial functioning (Magor-Blatch et al., 2014) compared to systematic reviews focused on substance use outcomes per se (Malivert et al., 2012). Research is unequivocal, however, in reporting very low rates of treatment completion due to a multitude of individual and program-specific factors (Prangley et al., 2018). Previous research data reported for Takiwasi Center, the current study site, are consistent with this, with non-completion rates measured at 51.8% (Defilippe, et al., 2019). Following their systematic review of European therapeutic communities, Malivert and colleagues (2012) found that, on average, participants stayed only a third of the planned time, with completion rates ranging from 9 to 56%. All studies included in their systematic review showed that substance use decreased during treatment and, while relapse was common, treatment completion was the most predictive factor predicting abstinence at follow-up. Relatedly, positive treatment outcomes appear to be associated with the duration of long-term residential treatment, up to about 18 months, at which point efficacy appears to level off and decline (Zhang et al., 2003). The long duration of therapeutic communities also ultimately means that far fewer people will be treated compared to outpatient services, which research has consistently judged to be more cost-effective than inpatient options for most clients (Martin et al., 1998; Martin and Rehm, 2012; McCarty et al., 2014), thereby mitigating any significant improvement in effective treatment coverage of the overall population in need. In short, such facilities need to be used judiciously.

To summarize, there is a role for long-term residential treatment in well-circumscribed, individual circumstances, and within a stepped-care continuum of services and supports. The extant literature also highlights the complexity of the therapeutic community as a treatment intervention and the need for more focused research on critical components and factors associated with treatment completion and positive treatment outcome (Aslan and Yates, 2015). This includes research on motivational and expectancy-based factors associated with seeking and completing such a long-term and high intensity treatment option. More research is also needed to identify alternative and innovative treatment models for people with complex refractory substance use disorders that have challenged treatment success in briefer, less intensive options within the continuum of care.

## Summary and Research Goals

To summarize, there is considerable anecdotal, as well as retrospective and prospective quantitative evidence about the therapeutic effectiveness of ayahuasca-assisted treatment for problematic substance use, including substance use disorders, as well as mood and anxiety disorders. Several plausible neuropharmacological, psychological and other mechanisms of action have been proposed, including therapeutic mechanisms specific to substance use disorders. Factors associated with positive treatment outcome have been investigated quantitatively and qualitatively. Experts in this area agree, however, that more research is needed with more diverse samples and cultural/therapeutic contexts, as well as more systematic investigation with longer follow-up and comparison samples where possible (Brierley and Davidson, 2012; Bouso and Riba, 2014). Given the important role of both set and setting in research on psychedelic-assisted treatments in general, more studies are also needed that are aimed directly at understanding this complex interplay. This will require a mixed-methods approach that would *integrate* quantitative data on treatment outcome with both an ethnographic description of the therapeutic context and qualitative assessment of the subjective experiences of participants, in particular perceptions and meaning attached to critical ingredients of the treatment experience and the outcomes attained.

Among the many alternatives available today for those seeking ayahuasca-assisted treatment for substance-related challenges, including severe substance use disorders, Takiwasi Center represents a unique opportunity to further our understanding of both the therapeutic value as well as the active ingredients of an integrative therapeutic model. The global challenge related to effective treatment coverage, coupled with the rapid growth of alternative ayahuasca-assisted options in Latin America and globally that incorporate this traditional ethno-medicine, increases the urgency of work to document treatment efficacy and explore the underlying mechanisms. Work is also needed on limitations, potential contraindications, and risks. There are also important opportunities to better understand the role of long-term residential treatment alternatives for substance use disorders and their place in the overall treatment continuum. In short, the overarching goal of such work would be to identify effective therapeutic components of ayahuasca-assisted treatment in different communities and cultures and translate these findings into improved treatment for substance use disorders in both traditional medicine and contemporary contexts.

Specifically, the four goals of the ATOP project currently underway at Takiwasi Center are to:

• Contribute to the understanding of ayahuasca-assisted treatment for substance use disorders, in particular the understanding of the role of set and setting from the perspectives of both staff and patients.
• Inform other potential ayahuasca-assisted modalities for treatment of substance use disorders, as well as psychedelic-assisted treatment generally.
• Contribute to the body of knowledge on treatment of substance use disorders and factors associated with positive treatment outcome in long-term residential treatment alternatives; and
• Identify strengths, challenges and limitations within the current Takiwasi treatment model so as to contribute to ongoing efforts toward quality improvement.

### Research Methods

#### Research Setting

The Takiwasi Center was established in 1992 in the city of Tarapoto, located in the Upper Amazonian region of Peru. Among the programs and facilities known to have incorporated ayahuasca into their treatment protocol for rehabilitation from substance use disorders, Takiwasi is no doubt the best-known such facility internationally. The treatment model is referred to internally as a ‘therapeutic tripod’: traditional Amazonian medicine (including the ritual use of ayahuasca in combination with other plant-based medicines), psychotherapy, and community living (convivencia).

The Center offers a variety of programs and services including ten-day plant medicine retreats (known as *dietas*), seminars, and a long-term residential treatment program for substance use-related rehabilitation, which is the main focus of Takiwasi and of the present research. The residential program is for men only, following Peruvian regulations requiring same-sex residential treatment services, modeled in large part after a therapeutic community (TC) model. As a TC there is a heavy emphasis on the role of community living in the therapeutic process, engendered by sharing work and maintenance duties, recreation, and personal friendships, as well as the inevitable interpersonal challenges. The other aspect of the treatment model combines “ancestral healing practices of folk healers and shamans with modern psychology” such as transpersonal psychology, the work of Carl Jung, and Gestalt therapy, among other influences (Mabit et al., 1996; Dupuis, 2018; O’Shaughnessy 2017). The treatment philosophy puts a strong emphasis on spirituality, particularly in the framework of what the institution refers to as traditional Amazonian medicine, which reflects a broader Andean-Amazonian cosmology in which all plants have a spirit (a *madre*) that confers a teaching (Jauregui et al., 2011). Takiwasi is not a religious institution; however, there is a notable Catholic influence and presence maintained by its co-founders and current leaders of the institution (Dupuis, 2018; Horák, 2013). There is an on-site chapel and a resident Catholic priest (*padre*) who performs Sunday masses, baptisms, and other Christian rites for those who wish to participate (O’Shaughnessy, 2017). In the ceremonial maloca,^1^ the walls are decorated with images representing the Christ, the Virgin Mary, and Saint Michael (Dupuis, 2021), thus the participants inevitably encounter Christian iconography whether or not they participate in chapel gatherings. The main building also contains iconography from other spiritual traditions as well, such as Buddhism, and linked to practices like yoga and meditation.

The model of addiction is embedded in a spiritual framework, wherein problematic substance use is conceived as a misguided spiritual ‘self-initiation’ that turns into harmful behaviors (Mabit et al., 1996). According to Mabit et al. 1996, Amazonian shamanism offers a ‘true’ initiation that can help individuals reorganize their inner Universe and reconnect with the sacred, thus reorienting the relationship with substances, with the self, and with the world (O’Shaughnessy, 2017). The philosophy at Takiwasi acknowledges that the contextual and ritualized use of sacred medicines in an appropriate setting (such as guided ceremonial ayahuasca use) can be highly beneficial for the individual. Indeed, the center clearly distinguishes ‘drugs’ and ‘drug use’ from ancestral medicines such as ayahuasca and *mapacho* (*Nicotiana rustica*). The program also recognizes that upon discharge participants may, if they choose, return to selective low-risk substance use. Thus, longer-term treatment goals are adapted to each individual and this may or may not include complete abstinence as an indicator of treatment recovery (Giove Nakazawa, 2002).

The Takiwasi treatment regime is highly structured, following many common practices of therapeutic communities in general. Before entry into the program, in addition to a typical clinical assessment, applicants must write a detailed life history that describes their childhood, upbringing, life goals, sexual relationships, and relationships with family, romantic partners, and work life, as well as their goals for the program and life in recovery. Prospective patients are then interviewed remotely and encouraged to visit the center. Inpatients sign a contract for a voluntary nine-month internment, divided into three “trimesters” or treatment phases. As per the contract, once treatment begins the inpatients must remain on the premises for the duration of the program. Each patient is assigned a therapist with whom they will meet weekly for the duration of the program. The first eight days are committed to detoxification, wherein the patient is isolated from the other inpatients and attended to by their therapist and the healers, while adhering to a regime of restricted diet, herbal saunas and baths, and drinking depurative plants. After enduring this initial stage, the individual officially joins the therapeutic community. In addition to individual psychotherapy sessions, patients participate in group therapy activities, ergotherapy, a variety of workshops (which might take the form of yoga, martial arts, dance, or sports), and are assigned various plant formulas for daily intake. Patients participate in weekly rituals with purgative plants, three to four eight-day isolation plant retreats (dietas), and ayahuasca ceremonies two times a month. The therapists visit patients every two days during their isolation retreats to help integrate the experience. During the dieta, a specialized Indigenous or mestizo healer or assistant attends to the patient one, two or three times a day to provide the assigned plants according to prescription. Throughout the program, patients are instructed on the significance of plants-as-teachers and the importance of the dieta and the ritual shamanic process now made accessible to non-Indigenous people through the Center’s psychology-oriented, syncretic and shamanic framework.

### Study Design

Study design is grounded on the premise that the treatment protocol at Takiwasi is a complex intervention, delivered within a unique organization with multiple mechanisms of action. In Takiwasi, there are many treatment components that include but are not limited to: the ingestion of ayahuasca in syncretic shamanic ceremony; traditional use of other key medicinal plants; ritual ingestion of purgative plants; daily ingestion of *plantas de contención* (containment plants, taken daily in lower concentration); individual and group therapy sessions; ergotherapy; and *convivencia.* All of these components provide the ‘setting’ of treatment and support, which interact with and alter the ‘set; ’ that is, as patients participate in different rituals and therapies over several months, their expectations and perceptions of the treatment and their own progress will necessarily change. Because of the complexity of the intervention and its organizational context, we draw upon the literature on the evaluation of complex interventions (Sridharan and Nakaima, 2020) and the role of context (LaFrance et al., 2012; Rog, 2012), so as to ensure critical attention to the interaction between set and setting. This approach takes into account not just individual elements such as complexity of patient profiles, previous treatment history and level of participation in various aspects of the program, but the programmatic and organizational context of Takiwasi as a whole. To better address this complexity, and consistent with the overarching ATOP protocol requirements, the research protocol is based on a mixed-methods approach to accommodate measurement of individual health and wellbeing outcomes over time, as well as assessment of interpersonal interactions and subjective reports on the significance of different treatment components. A mixed-methods approach is consistent with a complexity-based evaluation design as well as with trends in the literature on recovery from substance use disorders (e.g., American Society of Addiction Medicine, 2013; Kaskutas et al., 2014; Costello et al., 2018) that advocate going beyond assessment of abstinence to incorporate a broad range of metrics for positive treatment outcome (see also the International Consortium for Health Measurement Outcomes, 2020).

Our research design was also influenced by several practical considerations. Although Takiwasi serves our purpose as a naturalistic “living research laboratory,” there is not a realistic opportunity to randomly assign patients to alternative treatment conditions, including a no-treatment control group. We also recognize that randomized controlled trials are not without their own challenges (Cohen et al., 2004; Rothwell, 2005; Melberg and Humphreys, 2010; Hyde and Delphin-Rittmon, 2014), especially for complex interventions heavily influenced by set and setting (Stame, 2004). Lack of proximity to enhanced medical facilities also precluded incorporating a neuroimaging component despite early interest among the ATOP team in doing so. Constraints are also imposed by the length of the Takiwasi program (typically nine months) as well as anticipated high treatment drop-out rates, which we forecasted would present challenges in recruiting a study population of sufficient sample size for complex multivariate modeling of treatment exposure and outcome required to identify the most active programmatic ingredients from a statistic point of view. This further highlighted the need for a strong qualitative component to the study design and thoughtful integration of the qualitative and quantitative data.

**FIGURE 1:**
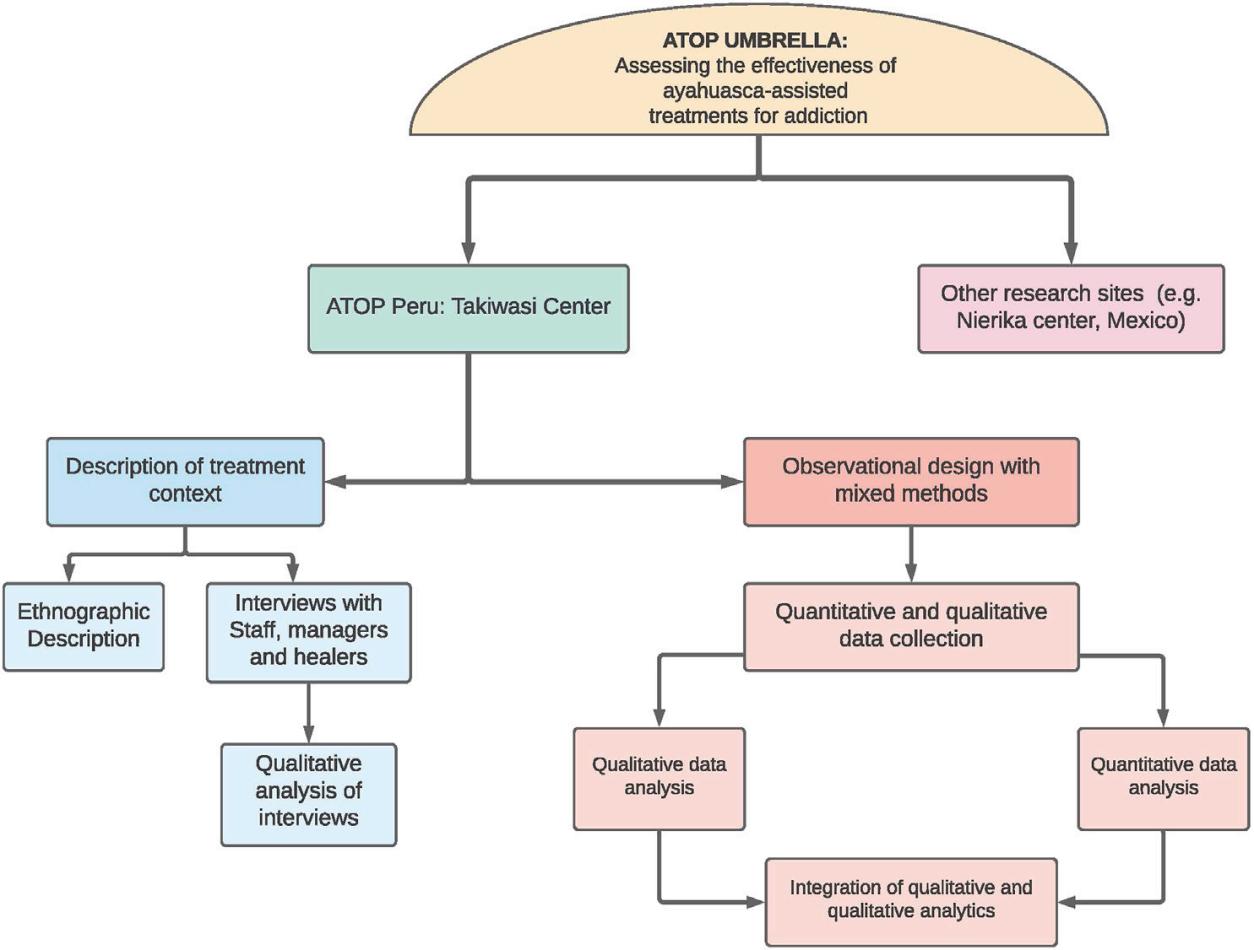
Overview of ATOP Study Design.

**FIGURE 2:**
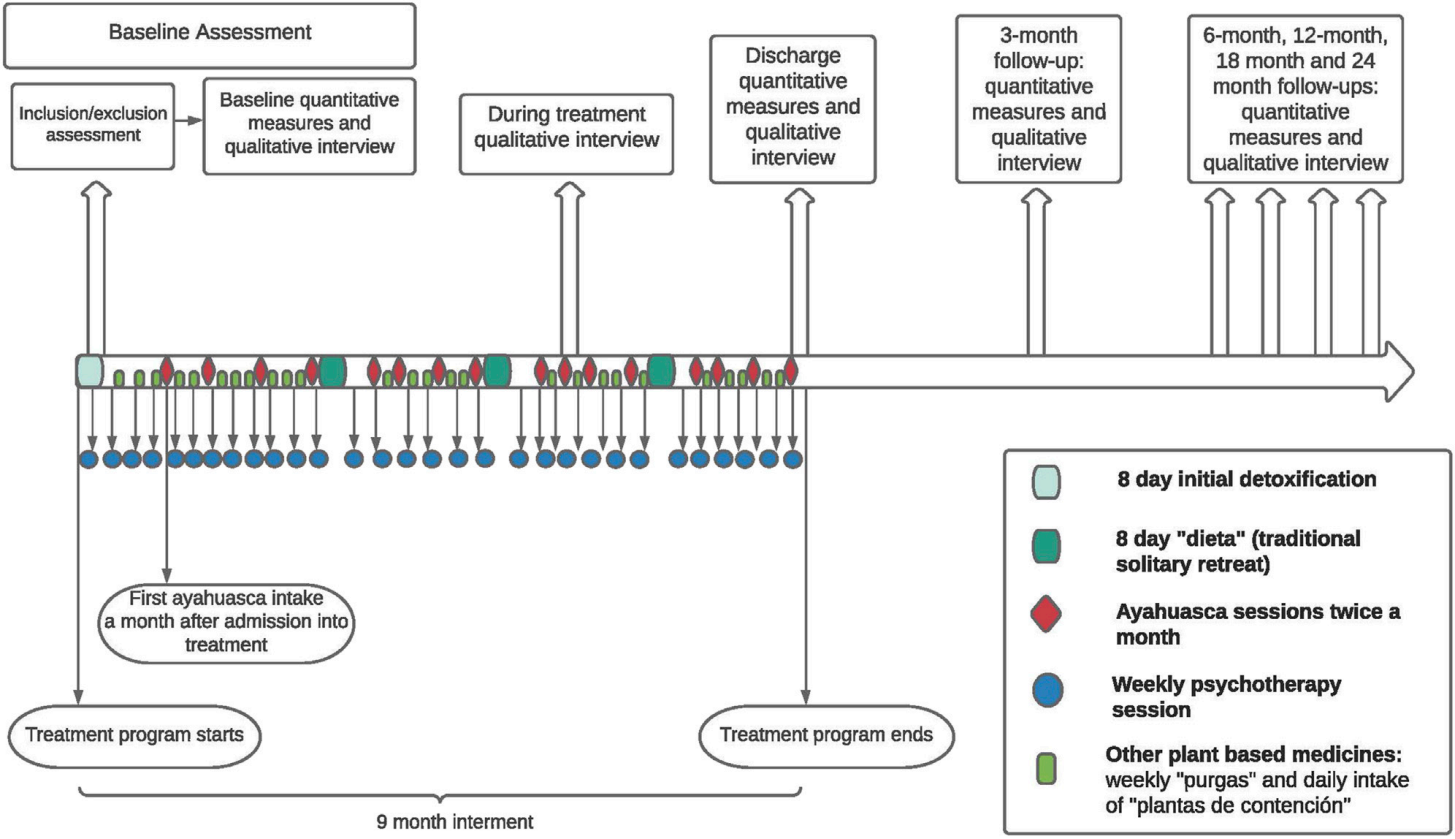
Timeline.

Consistent with the above considerations, a research design was formulated that is appropriate and feasible within the Takiwasi context. Figure 1 provides a high-level overview of the various components of the ATOP protocol implemented at Takiwasi with Figure 2 providing a more detailed timeline for the various measurement processes in relation to the various elements of the Takiwasi therapeutic processes. Specifically, a prospective cohort design using a set of core quantitative measures at baseline (i.e., program intake), discharge and 3, 6, 12, 18-months and 24-months follow-ups. The baseline assessment includes a research-based, psychiatric interview to define status regarding substance use and other mental disorders by international standards–The MINI International Neuropsychiatric Interview (Lecrubier et al., 1997; Sheehan et al., 1998). The purpose of this structured interview is for recruitment (i.e., eligibility depends on a research diagnosis of alcohol/drug abuse or dependence and for purposes of sample description and sub-group analysis according to mental health co-morbidity, e.g., co-occurring depressive or anxiety disorder). Following completion of this diagnostic instrument and other aspects of eligibility determination (i.e., assessment of cognitive impairment (Katzman et al., 1983), the remaining baseline measures are administered. These measures cover severity of substance use disorder, quantity and frequency of substance use, motivation for treatment, a range of mental health measures, quality of life, and spirituality. The specific instruments used are identified below according to study phase, all but one of which has been validated in Spanish, that being the Treatment Entry Questionnaire, which the study team translated and back-translated according to accepted international guidelines. Although initially conducted via telephone on Internet (e.g., Skype), the follow-up quantitative data collection was converted to an on-line platform for purposes of cost-efficiency and with personal support available as needed by the respondent. Importantly, all follow-up contacts are initiated and supported by the Research Coordinator who is independent of the programmatic structure of the Takiwasi treatment program itself. First contact with the follow-up Research Coordinator is immediately after inclusion of the participant in the study and then regularly thereafter to book future appointments and maintain accurate information on the client’s living situation.

The qualitative component of the mixed methods design includes patient semi-structured interviews undertaken in the same temporal sequence as the quantitative measures: that is at program entry, during treatment, at discharge, and at each follow-up point. To improve understanding of the treatment setting (i.e., environment and interpersonal interactions), patient interviews are complemented by semi-structured interviews with staff, therapists and healers as well as a contextual description of the Takiwasi treatment center based on existing literature (Mabit and Sieber, 2006; Håland, 2014; O’Shaughnessy, 2017; Mercante, 2013; Dupuis, 2018; Dupuis, 2021; Politi et al., 2019). A sub-project was also initiated part way through project implementation to identify factors associated with non-completion of the program.

The research protocol at Takiwasi was approved by the Comité de Ética PRISMA, *Lima*, Perú.

### Measures

*Quantitative* Table 1 identifies the quantitative measures used to establish a baseline and measure change over time, as well as treatment motivation at treatment entry, an important potential modulator of outcomes.

**TABLE 1:** Quantitative measures used at baseline and post-treatment.

Quantitative measure	Description
Addiction Severity Index—5th Version (ASI-5)	ASI scores provide a general profile of problem severity in specific areas (drug use, employment, medical and psychiatric disorders, family/social relationships, legal status) and is a proven international standard for treatment planning and post-treatment outcome evaluation. This project employed the Spanish version of the ASI-5 McLellan et al. (1992)
Global Appraisal of Individual Needs –Substance Use Grid	This instrument comprises a small set of items and subscales of a much larger tool used for treatment planning and process and outcome evaluation for substance use treatment - the GAIN-I Dennis, (1999); Coleman-Cowger et al. (2013). The tool is needed to supplement the ASI-5, which does not provide sufficient detail on actual drug use, or treatment history. The GAIN-I, including these sub-scales, has been translated into Spanish. The sub-scales measuring drug use have been adapted to reflect street names of various drugs in the peruvian and Latin american context
Beck Depression and Anxiety Inventories (BDI and BAI)	These are self-administered instruments to measure symptoms of depression and anxiety with 21 items rated on a scale from 0 to 3 Beck et al. (1988); Steer and Beck, (1997). Results are used to classify depression and anxiety as minimal, mild, moderate and severe. They are validated in Spanish Bonicatto et al. (1998); Vega-Dienstmaier et al. (2014)
WHOQOL-BREF—Quality of Life	The WHOQOL-BREF instrument comprises 26 items, which measure the following broad domains: Physical health, psychological health, social relationships, and environment. The WHOQOL-BREF is a shorter version of the original instrument WHOQOL-100 Saxena et al. (2001); The WHOQOL Group, (1998). It assesses the individual's perceptions in the context of their culture and value systems, and their personal goals, standards and concerns. The WHOQOL instruments were developed collaboratively in a number of centers worldwide and have been widely field-tested. The Spanish version has shown good psychometric properties Lucas-Carrasca, (2011); Saxena et al. (2001)
WHOQOL-SRPB—Spirituality, Religiosity and Personal Beliefs	The WHOQOL-SRPB consists of 32 questions, covering quality of life aspects related to spirituality, religiousness and personal beliefs (SRPB). This instrument has been developed from an extensive pilot test of 105 questions in 18 centers around the world. The resulting 32-item instrument represents the finalized version of the WHOQOL-SRPB to be used for field trials. It is available and tested in Spanish Lucas-Carrasco,( 2011)
Treatment Entry Questionnaire (TEQ-9) [BASELINE ONLY]	This brief nine-item questionnaire measures motivation for treatment based on self-determination theory and contains three separate, validated dimensions that relate to identified motivation (e.g., sought treatment because really identified with goals of the program and wanted to make changes); external motivation (e.g., sought treatment because other people pressured); or introjected motivation (e.g., sought treatment because of internal conflict such as to avoid feeling ashamed) Wild et al. (2006); Urbanoski and Wild, (2012); Wild et al. (2016). The instrument has been translated and back-translated to Spanish

*Qualitative* Semi-structured interviews are conducted with recruited program participants at different points: baseline, during treatment, discharge, and each point of follow-up, synchronized with completion of the follow-up quantitative measures. The semi-structured interview guides are included in Supplementary Material.

*Baseline* Information gathered at baseline includes the duration/trajectory of substance use problems and previous formal and informal treatment experiences within this trajectory. Life experiences related to overall health and mental health are also explored. Other areas of questioning include motivations and expectations for the program experience lying ahead, previous experience with ayahuasca, psychedelics, and religious or spiritual practices.

*During treatment and discharge* Since Takiwasi uses ayahuasca in a manner that draws heavily upon Peruvian practices, the specific questions in the interviews during the treatment process reflects these practices. Topic areas for the interview include: general perceptions of the overall Takiwasi treatment experience; the experience with ayahuasca and the importance of subsequent integration activities; and the perception that this has led to changes in the person’s current sense of wellbeing or life satisfaction.

A framework developed previously by Loizaga-Velder and Loizaga (2014) was used to develop the questions that explore the dimensions of the subjective experience with ayahuasca and its therapeutic context. Following this framework, specific feedback is sought on various elements of the overall ceremonial/therapeutic process, including elements of the ceremonial experience (i.e., ceremonial songs [*icaros*], energetic interventions by curandero/a, purging, ritual blowing of tobacco smoke [*sopladas*], *perfumes/aromas*); activities aimed at integration (e.g., psychotherapy or other therapeutic assistance, group sharing); other relevant healing activities such as yoga, exercise or meditation; *purgas* (i.e., therapeutic use of emetic plants); *dietas* (isolation retreats supported by psychoactive plants); shared group experience (e.g., group support, catalyst for group dynamics); and aspects of the treatment milieu (e.g., activities together, gardening, sharing or cooking meals). These areas queried with a five-point rating scale in terms of perceived importance followed by an opportunity for open-ended comment and clarification on the most and least helpful therapeutic elements. The rating scale responses are transferred to the quantitative data file for analysis. Although there was one Likert-type question concerning the Catholic practices of ‘liberation’ and ‘genealogic tree masses’, there were no focused questions or qualitative inquiry concerning perceptions and experience of religious elements (namely Christianity or other syncretic elements) woven into ritual-therapeutic treatments.

The core themes analyzed in the baseline and follow-up qualitative interviews are presented in Table 2.

**TABLE 2:** The core themes analyzed in the baseline and follow-up qualitative interviews.

Data collection phase	Core themes explored
Baseline interviews	Ayahuasca, psychedelics, shamanism (e.g., experience, learnings, worries, expectations before coming to takiwasi)
	Sociodemographic data
	History/context of personal and familial substance use
	Perceptions of mental health and healthcare systems
	Spirituality/religiosity
	Takiwasi-related themes (e.g., reasons for coming; concerns and expectations about treatment; initial perceptions of takiwasi)
	Treatment capital (e.g., previous substance use and/or mental health treatment)
During treatment, discharge, and post-discharge interviews	Meanings/personal significance (e.g., building upon previous treatment experience)
	Program experience (e.g., continuity between ayahuasca sessions; effect on craving; perceived addictive potential; frequency of sessions)
	Recovery process (e.g., perception of benefits, challenges, ability to integrate experiences and learnings into everyday life)
	Satisfaction with services (e.g., overall perception of services received and suggestions for improvement)

#### Study Recruitment and Measuring Treatment Exposure

The status of participant recruitment is summarized below. As of December 17, 2020, a total of 128 participants had completed intake into Takiwasi and were potentially eligible for ATOP. Of these, 42 left the program before participating in any ayahuasca ceremony as part of their treatment. A total of 86 initial participant interview were undertaken at baseline and, of those scheduled for their one-year follow-up (n-60), 48 have completed both the one-year qualitative and quantitative interview, representing an 80% one-year completion rate. The current sample size at 18-months (*n* = 30) and at 24-months (*n* = 25) reflect on-going scheduling and are not reflective of drop = out during follow-up.

**FIGURE 3:**
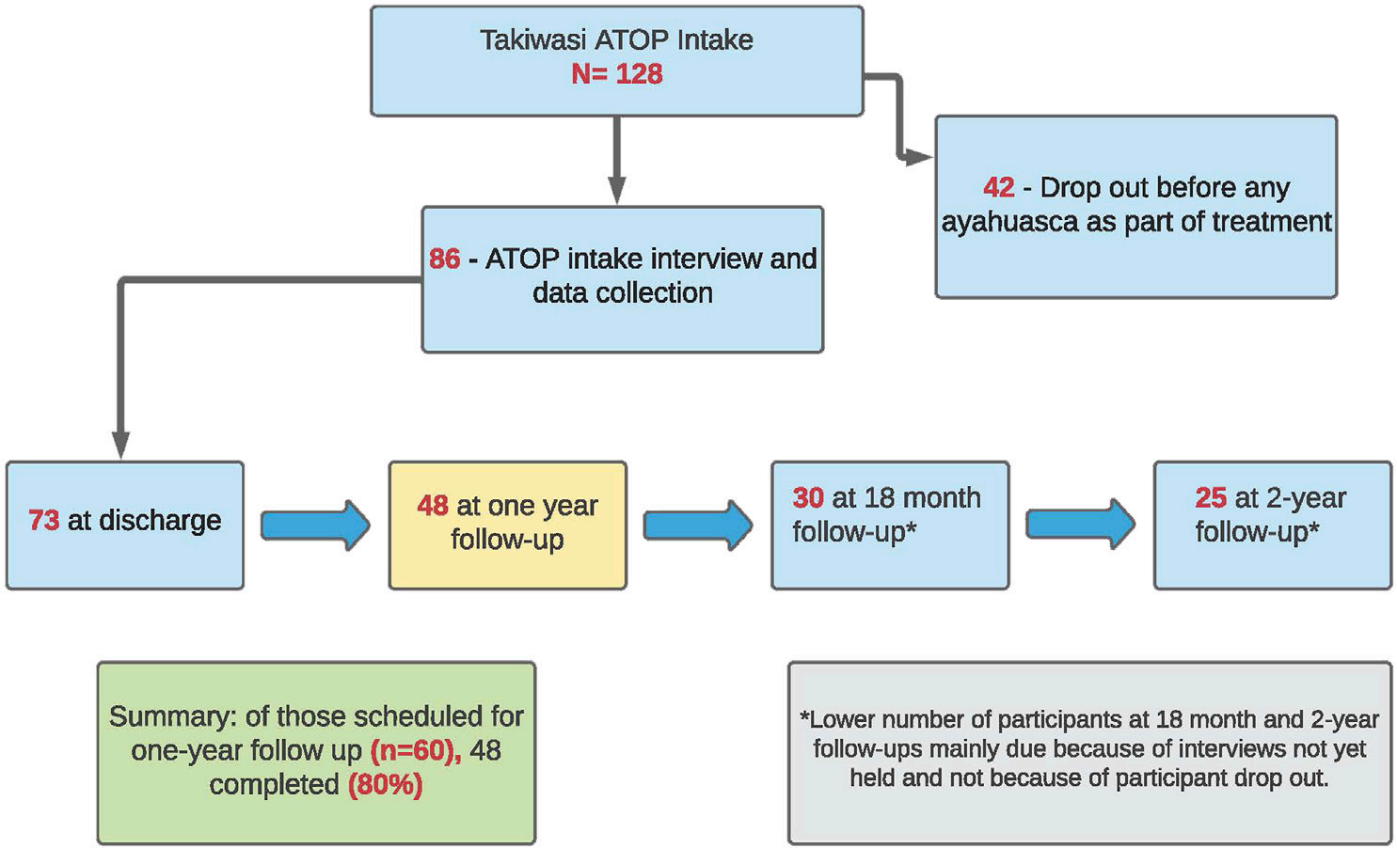
ATOP recruitment overview-Takiwasi (as of Dec. 17, 2020).

As shown in Figure 3, a significant proportion of clients who initiated services at Takiwasi during the study period left the program before any use of ayahuasca (42 of 128 or 32.8%), and therefore, are not eligible for study inclusion. As noted earlier, such high drop-out rates are common in long-term therapeutic community programs and may include reasons that concern the program itself (e.g., too long; content didn’t fit with expectations and personal values); the program environment (e.g., the nature and type of facility or community context); or personal issues (e.g., family or employment concerns made it difficult to continue; psychological issues such as resistance to continued inner work or drug cravings). Some leave treatment programs against advice of program staff and others leave for such personal reasons. This presents a challenge for any observational study with a follow-up component in substance use treatment services since those who finish the program and/or who are located for follow-up may be different in meaningful ways than those who leave the program prematurely.

A related challenge that is important to account for in the study design and analysis is the level of treatment exposure for those formally accepted into the study cohort after the initial ayahuasca experience. We approach the measurement of treatment exposure in two ways. First, with respect to treatment completion the study cohort is divided into three sub-groups: 1) completed treatment; 2) left the program prematurely but voluntarily; and 3) suspended from the program. For the current 12-months sample of 48 clients, the number in each group is 30 (60%); 14 (29%); and 4 (8%), respectively. While all three sub-groups are included in the discharge and follow-up samples, the number of weeks of treatment participation is calculated for each patient. The second approach to measuring treatment exposure is based on an extract of information from the patient files concerning the nature and extent of participation in program activities, for example, the number of ayahuasca sessions, days of dieta, integration sessions with staff. A program participation index will be calculated from these extracted data, also for inclusion in the analysis.

To investigate the potential for response bias in our study sample due to early or subsequent treatment drop-out, a process has been undertaken to locate clients who left prematurely in order to explore reasons for leaving the program. At the time of this writing, the questionnaire has been drafted (see Supplementary Material) and the survey is being mounted for on-line completion. Once finalized, those recruited for the study who have left prematurely will be contacted using the last available email address and a request made for completion of the questionnaire. Also, characteristics of program completers and non-completers will be compared (e.g., age, country of origin; results of the intake psychiatric interview and/or other baseline data).

### Interviews with Managers, Staff and Healers

In addition to the interviews with program participants, semi-structured interviews are conducted with managers, staff, therapists, and healers (see Supplementary Material). This interview captures the person’s role in the center, their professional formation, their own personal religious and spiritual practices, and their use of, and beliefs about, alcohol or other drugs and related treatment practices. Other questions query their training and experience working with ayahuasca, narratives about their own ayahuasca experiences as well as those specifically related to possible links between ceremonial experiences and people’s subsequent change in behavior or habits related to alcohol and other drugs. Narratives also explore their beliefs and experiences regarding the relationship between the different elements of the Takiwasi ‘therapeutic tripod’ in facilitating behavioral changes related to use of alcohol or other drugs.

A total of 17 interviews have been conducted as of December 17, 2020.

### Contextual Description of Takiwasi Center

Consistent with the overall ATOP protocol, the key elements of this contextual description include the program’s *physical and organizational infrastructure* (e.g., physical space, management structure, budget, length of program); *staff/personnel* (e.g., number and type of therapists/staff, the training and experience of curanderos/as and therapists); *prevailing beliefs and mores about addiction* (e.g., curanderos/as and therapists beliefs with respect to causes of addiction, the critical ingredients of effective treatment and definition of good outcome as well as the most common success factors and challenges for people seeking to make changes); *interventions provided*, for example, the combinations of various plants with or without ayahuasca, use of psychiatric medication or other therapeutic techniques, strategies for safety, integration, follow-up and/or continuing care; cultural and community context (e.g., relationship to Indigenous communities, community support and capacity building). See Supplementary Material for guide for site description.

#### Analysis Plan

While the details of the analysis plan differ significantly for the qualitative and quantitative components of the mixed methods, they share several common elements: 1) guided by specific pre-determined research questions in support of the broader project goals identified above; 2) undertaken by two analytic teams working independently and according to their area of expertize (i.e., qualitative or quantitative) but meeting bi-weekly as a unified project team to discuss progress, and develop a consensus on critical questions and issues brought to the meeting from each team.

With respect to the research questions guiding the analysis, these questions were developed by the overall team to create an overarching analytic framework, with the quantitative and qualitative analyses contributing differentially to each question. For example, with respect to Takiwasi patients, both quantitative and qualitative analyses were aimed at the following questions:

• What is the individual’s previous experience with substance use treatment and what learnings and challenges are being brought into this new experience at Takiwasi? Is previous treatment experience associated with treatment outcomes?
• What is the individual’s situation at treatment entry with respect to physical and mental health challenges and overall life satisfaction? Are baseline physical and mental health challenges associated with treatment outcomes?
• What meanings are attached to the overall Takiwasi treatment experience as well as key elements (e.g., treatment milieu, specific medicines, dieta, purging, icaros, therapeutic integration) and how have these meanings evolved during the course of the program? What ratings attached to the significance of these experiences are most closely associated with treatment outcome?

The key questions specific to the quantitative analysis are:

• What are the outcomes achieved at 12 months post-discharge (e.g., self-reported reduction in substance use and overall addiction severity; improvements in symptoms of anxiety and depression; change in overall quality of life)?
• What factors are independently associated with these 12-months post-discharge outcomes (e.g., treatment motivation; baseline addiction severity or mental health status; discharge status on improvements in mood and anxiety or spirituality).

Examples of questions that are the exclusive focus of the qualitative analysis of patient data include:

• What is the person’s subjective understanding of their substance use-related challenges and what is the match between the individual’s perspective and the treatment provided at Takiwasi?
• How do clients perceive their recovery process at different follow-up points? What outcomes are reported as being the most meaningful?
• Do participants report incorporating learnings and experiences during treatment into their life after discharge?

*Quantitative analytics—Patients* The quantitative analysis team is composed of three team members, aided by an external native Spanish-speaking volunteer. Among the formal team members, one is a native Spanish speaker and two speak Spanish at an intermediate level. The data preparation and analysis will proceed through six steps: 1) preparation of a unified, person-level Excel data file that includes all baseline and subsequent quantitative data points as well as abstracted information from patient files; 2) descriptive analysis of the characteristics of the study sample with frequency distributions and cross-tabulations (see Supplementary Table S1); 3) descriptive analysis of patients’ treatment participation and perceived importance of Takiwasi treatment activities (see Supplementary Table S2); 4) visualization of trends over time across the range plethora outcome measures (See Supplementary Table S3). Importantly, statistical significance testing is not performed on any observed changes over time in these multiple outcomes so as not to violate the principles on which the subsequent stage of multivariate modeling is based (i.e., mitigating increased odds of Type 1 error across multiple, non-theory driven comparisons) (Kuchibhotla et al., 2020); 5) a team-based, measures minimization process so as to reduce the number of predictor and outcome variables based on previous research and a priori conceptual modeling of hypothesized relationships. For example, of the many measures of substance use and problem indicators, one or possibly two primary substance use outcomes measures will be identified, and as well many potential predictor and moderator variables associated with mental health, treatment motivation, treatment participation and ratings of program experience. Finally, 6) Preparation of DAGs (Directed Acyclic Graphs) to conceptually model the hypothesized relationship between predictor and moderating variables and outcome. These DAGs then serve as the basis for multivariate regression modeling (Herman and Robins, 2020). Regression analyses will start with less complex, three to five variable DAGS and build from there based on variance explained through addition of predictors and moderators (Hernan and Robins, 2020) with due consideration for statistical power. See Supplementary Figure S1 for an example of a hypothesized and relatively complex DAG.

Post-discharge outcomes relative to baseline will be analyzed with a generalized linear mixed effects model. Random intercepts (and random slopes where necessary) will be included to account for correlation between observations within study participants. The regression models will also include (potentially time-varying) baseline and within-treatment covariates (e.g., treatment motivation, baseline addiction severity, and number of hours engaged in non-ayahuasca treatment activities such as psychotherapy, group integration, ergotherapy). Statistical inference will proceed in a manner consistent with the DAGs constructed before any models are fit. The relevant parameter and variance estimates associated with all models that are fit will be reported.

*Qualitative analytics—Patients* The qualitative team is composed of four researchers, two of which are native Spanish-speakers, and the other two speak Spanish at an expert level. Qualitative analysis will employ a grounded theory approach (Miles and Huberman, 1994; Strauss and Corbin, 1994), in which the team creates codebooks based on themes that emerge from the interview data with inpatients. Grounded theory approaches work best with relatively open-ended questions and narratives which allow for deep analysis and emergence of key themes, which ultimately become integral to building a case-specific theory about outcomes related to set and setting at Takiwasi. In line with this approach, the original guiding research questions may be modified in an iterative process according to significant themes that emerge in the data.

The team selected a cloud-based qualitative data analysis program (www.dedoose.com) through which they can remotely cross-code data and conduct collaborative analyses. Analysis will focus on several themes guided by the specific research questions, including recovery capital, expectations and perceptions of treatment, spirituality, religiosity, and social influences, among others.

*Integrative quantitative and qualitative analytics—Patients* A framework has been developed to guide the integration of the quantitative and qualitative analysis according to a constant comparative approach to shared evaluation questions. Supplementary Tables S1–S4 illustrate this approach. An important part of this integrative process will involve stratifying the study participants into three groups on the basis of a consensus rating by the project team of each patient’s overall treatment outcome. For convenience purposes, the three groups will be labeled only, as “very poor/poor,” “moderate,” “very good/excellent.” The qualitative themes related to treatment outcome will then be cross-referenced within each of these three groups with the aim being to identify themes reflecting important predictive or moderating factors. These analyses will complement the multivariate modeling based on the DAGs.

*Qualitative analytics* Managers, staff, therapist and healer interviews: As with the patient interviews a set of pre-defined research questions will guide the analysis of these interviews. The primary research focus for these analyses concerns the homogeneity of the therapeutic culture at Takiwasi. Emergent themes will also be identified concerning the convergence and divergence of the Takiwasi therapeutic culture over time. The research questions and their respective aims are:

• What are therapists’ main theoretical training and has it influenced their work at Takiwasi (Aim: To identify patterns in practical or theoretical background among practitioners who gravitate toward Takiwasi).
• Do therapists utilize their non-psychotherapeutic skills with clients at Takiwasi (e.g., reiki, yoga, etc) (*Aim:* To determine how many alternative healing practices are being applied during clients’ residency).
• What are therapists' views on religion? How do their personal views relate to practices at Takiwasi or their relationships with clients (Aim: To explore the subject of institutional religiosity, including the role of Christianity within the context of spirituality).
• How has Takiwasi affected therapists' perceptions of care, psychology/psychotherapy, and practice? How did their time at Takiwasi influence their proximate career goals (Aim: To explore the 'training' aspect of Takiwasi and how it influences practitioners).
• What are the prevailing views and subjective understanding of addiction and related challenges (Aim: to explore the match between practitioners and the treatment provided at Takiwasi).
• Do practitioners link their own experiences with ceremonies and dietas to therapeutic efficacy and/or therapeutic alliance (Aim: To determine the role of ayahuasca in developing a unique kind of therapeutic alliance).
• Are there specific aspects of the ayahuasca experience (e.g., dietas, icaros, purging, cleansing/limpiezas, visions, death experiences) that managers, staff, therapists and healers think are particularly helpful for healing? For alcohol and drug addiction specifically (Aim: To explore potential links between prevailing views on program components and treatment outcomes).

The qualitative analysis team will employ a grounded theory approach, creating codebooks based on themes that emerge from the interview data with these Takiwasi stakeholders. In line with this approach, the original guiding research questions may be modified in an iterative process according to significant themes that emerge in the data. The same Dedoose qualitative analysis program will be employed so as to work remotely and collaboratively.

## Discussion

Considerable research has pointed to the therapeutic value of ayahuasca and ayahuasca-assisted treatment for substance use disorders (Thomas et al., 2013; Bouso and Riba, 2014; Nunes et al., 2016; Argento et al., 2019) as well as other mental health challenges, in particular depression and anxiety (Osório et al., 2015; Tupper et al., 2015; Palhano-Fontes et al., 2018; Muttoni et al., 2019), many of which co-occur with problematic substance use (Chan et al., 2008). This interest in ayahuasca coincides with a resurgence in interest and research in psychedelic medicine, including chemically similar pharmacological compounds such as LSD and pharma-grade psilocybin (Muttoni et al., 2019; Brierley and Davidson, 2012). Research on ayahuasca also represents a growing interest in traditional medicine in general, which includes, but is by no means limited to, other entheogens such as peyote, ibogaine, and psilocybin mushrooms. Indeed, ayahuasca is poized to be situated on the bridge between ancestral medical traditions and the modern, westernized medical application of psychedelics. In both ancestral and modern applications, the role of set and setting, more recently framed as the therapeutic “context” (Carhart-Harris et al., 2018c) have important relationships to therapeutic benefit as well as risk. Importantly, the role of “context” resonates with developments in the science of program evaluation, in particular realist evaluation and complex interventions (Pawson and Tilley, 1997; Rog, 2012), and summarized succinctly by the phrase: “outcome = intervention + context”.

Building upon a strong foundation laid in the multidisciplinary workshop held in Tarapoto, a comprehensive evaluation protocol was developed to investigate outcomes associated with ayahuasca-assisted treatment for substance use disorders at Takiwasi Center. This institution was determined to be an excellent starting point for what was envisioned as a multi-site research project with a common protocol. Advantages associated with Takiwasi as a study site include its history of support for internally and externally driven research and evaluation, and the syncretic nature of its organizational environment and treatment program. This diversity provides fertile ground for testing a protocol designed from the outset to investigate the role of context in the determination of treatment outcomes.

The research protocol, described above in detail, is poized to make a significant contribution toward each of four study goals. Our first goal is to contribute to an understanding of ayahuasca-assisted treatment for substance use disorders. Our mixed methods approach within a longitudinal cohort design, situated in the context of complexity-based evaluation, will be critical to identifying key elements of set and setting that are most closely associated with treatment outcomes. To this end, the major strengths of the protocol include: the use of multiple, validated measures of predictors, potential moderators, and outcomes, which are assessed at multiple time points from program entry through 24 months post-discharge; multiple measures of program participation and satisfaction with services; and state-of-the-art statistical testing methods that will minimize risks of Type I error that can arise through multiple, non-theory-based significance testing. The comparative approach to addressing research questions via complementary quantitative and qualitative analytics is similar conceptually to that employed by Thomas et al. 2013 and Argento et al. (2019) in their study of ayahuasca-assisted treatment for substance use disorders in Canada, but now advantaged with a more extensive baseline assessment of patient characteristics, a larger sample size and longer follow-up period. As the current research progresses toward its first analysis and reporting on one-year follow data, it will prove to be the most rigorous and comprehensive study to date focused exclusively on ayahuasca-assisted treatment for substance use disorders.

The second study goal is to inform other potential ayahuasca-assisted treatment modalities for substance use disorders, as well as psychedelic-assisted therapy more generally. It is anticipated that the study results concerning the role of the various non-pharmacological elements of the treatment context will be useful to others that wish to incorporate this medicine into a structured treatment process. These critical ingredients may also inform the development of other psychedelic-assisted treatments for substance use and other mental health disorders as well as their evaluation. Following Carhart-Harris et al. (2018c), our findings may point to key features of treatment context worthy of pursuing with a more controlled clinical trial design; for example, music, psychotherapeutic integration, and treatment milieu. It will be particularly important to explore what aspects of the treatment model under investigation in this project might translate to more readily accessible outpatient services using ayahuasca- or other psychedelic-assisted interventions, and for whom they are most appropriate. In translating the findings to other treatment modalities, it will be important to maintain the focus on treatment set and setting as critical ingredients of the treatment intervention itself, and not viewed as an element of potential placebo to be controlled out of the outcome equation. Using our study as an illustrative case-in- point, the research is conducted in a naturalistic setting and focuses on evaluating outcomes of an integral treatment program as a whole. As such, the drinking of ayahuasca intake and subsequent experiences occur within a complex setting following the Amazonian tradition, where different practices take place (e.g., singing, application of perfumes, and perhaps intake of other psychoactive medicinal plant preparations like tobacco juice) during the same session. From a theoretical point of view, according to the cosmovision inherent in Amazonian traditional medicine, as well as the clinical experience of the Takiwasi Center, the visionary and psychedelic component of ayahuasca is not the only, nor necessarily the most important, therapeutic mediator. Indeed, Amazonian healers point emphasize the importance of the ritual as whole to trigger the healing process. Moreover, in the Amazonian indigenous and mestizo context, patients frequently do not take ayahuasca in the ceremony and it is rather just the healer who uses the psychedelic-visionary effect to facilitate diagnosis and treatment choice.

With respect to furthering work with ayahuasca or other psychedelic-assisted interventions for substance use disorders, we also advocate that close consideration be given to our comprehensive set of measures so as to facilitate cross-study comparisons. Notably, our measures map well onto the recommended outcome domains for evaluating substance use treatment (International Consortium for Health Measurement Outcomes, 2020). Further, we aim to demonstrate that situating this measurement model within a mixed methods study design will bring significant value for moving the field forward and, to this end, all measurement tools used in the study protocol are provided as easily accessible Supplementary Material, including the semi-structured interview guides. Pending resolution of importation challenges for bringing ayahuasca to Mexico for research purposes, we plan for at least one additional ATOP site, and more may eventually be added consistent with ATOP study criteria. The common measurement model and mixed methods design sets the stage for a multiple case study approach, with Takiwasi Center being the first chapter in the story of ayahuasca-assisted treatment for substance use disorders based on the ATOP protocol.

Further with respect to the ATOP measurement model, the team elected not to include measures of the immediate psychedelic (e.g., Hallucinogenic Rating Scale; Strassman et al., 1994) or “mystical/peak” experience (e.g., Revised Mystical Experiences Questionnaire (Barrett et al., 2015) to correlate with treatment outcomes. While the measurement of mystical experiences has proven valuable in lab-based clinical trials (e.g., Rothberg et al., 2021), and many other types of psychedelic research (Johnson et al., 2019), it was determined not to be a good fit for the longitudinal, naturalistic design of ATOP. From a practical viewpoint, the ayahuasca used at Takiwasi is consumed on multiple occasions from different batches, and for this reason will undoubtedly vary to some degree in pharmacological composition and effects. More important, however, is that the purpose of this study is *not* to assess specific effects of ayahuasca as a dose-response mechanism, nor aim to systematically disentangle pharmacological vs. non-pharmacological factors, but rather to evaluate an established intervention in a naturalistic setting. In this naturalistic context the mystical experience in any specific ceremony is neither necessary nor sufficient to explain outcomes associated with the multiple therapeutic aspects of treatment at Takiwasi for the duration of a patient's nine-month participation.

The third study goal is to contribute to the body of knowledge on treatment of substance use disorders and factors associated with positive treatment outcome in long-term residential treatment alternatives. In part, with this protocol we will advance understanding of the effectiveness of the long-term residential therapeutic community treatment model and its role within the overall continuum of treatment and support. In particular, we answer the call for more research on TC’s to look inside the proverbial “black box” to investigate the role of different elements of this complex treatment model in improving treatment completion and different types of outcomes. Does ayahuasca-assisted treatment within the therapeutic community model alter the trajectory of treatment completion and outcomes, and for whom? How do participants rate the importance of conventional elements of the model (e.g., treatment milieu, therapy) as compared to elements incorporated from traditional medicine and other syncretic sources? What role does spirituality play within the spectrum of outcome measures and in sustaining post-discharge improvements? For example, continuation of spiritual practices and related distinctions between safe and harmful use of psychoactive substances. What might we learn from Takiwasi’s spiritual model of addiction, including how it is operationalized through the syncretic integration of shamanic, Christian and other spiritual practices and their perceived importance in determining the outcome of treatment? Might information in this area provide insight into how the TC model, and other treatment models and modalities, could be enhanced within a broad bio-psycho-social-spiritual framework? Indeed, the results concerning the role of spirituality as a potential predictor of outcome, and as an outcome in its own right, will have implications for the field of substance use treatment writ large, given its long-standing interest in spirituality (Geppert et al., 2007; Miller and Bogenschutz, 2007).

Fourth, and last, the research protocol aims to identify strengths, challenges and limitations within the current Takiwasi treatment model so as to contribute to ongoing efforts toward quality improvement. Although a great deal of research has been supported by and undertaken by the Takiwasi Center, to date there is, no clearly documented process by which this research contributes to service enhancement. Sridharan and Nakaima (2011) speak to the strengths of realist, complexity-based evaluation in building evaluation capacity within an organization. It is with this spirit that the project team anticipates significant knowledge exchange opportunities among Takiwasi Center managers, staff, therapists, and healers to both debate the results and maximize opportunities for individual and organizational learning and growth.

### Limitations and Conclusion

It is important to highlight some of the limitations of the study protocol in achieving its ambitious goals. Despite the many strengths of studying ayahuasca-assisted treatment in a naturalistic setting, there are some uncontrolled factors that hinder our ability to suggest precise conclusions about efficacy. For example, the precise composition of the ayahuasca tea and the dosage of consumption (ml/kg) are not available. Similarly, we could not measure the phenomenological strength of the psychedelic experience (e.g., Barrett et al., 2015). Further, the basic study design precludes strong statements of causality, although it’s important to note considerable expert opinion in the field of psychedelic science about the limitations of clinical trials in studying these complex interventions that are so heavily dependent on treatment context. And while our protocol may identify particular aspects of treatment context that could potentially be manipulated in an experimental design, we remain very cognizant of the perspective of healers experienced with this traditional medicine that the healing potential is in the “gestalt” of the practice and the participant’s experience (Berlowitz et al., 2018).

Lastly, we note the high rate of program not-completion, a factor common to TC’s and clearly a concern with respect to Takiwasi Center specifically and a source of potential bias. To mitigate this concern to the extent possible, a sub-project has been initiated to follow-up with all Takiwasi study patients who have left the program prematurely to explore reasons for leaving the program. The study protocol also includes a variable that measures length of program participation and which will be used a potential moderating variable in the multivariate analysis. Aside from controlling for potential bias, we also view this as an important aspect of the study itself given positive outcomes that have been obtained with a single dose of ayahuasca (Osório et al., 2015; Sanches et al., 2016; Palhano-Fontes et al., 2018), and potential implications for Takiwasi and other ayahuasca-assisted treatment modalities.

These limitations aside, our protocol serves as an important example of the comprehensive approach needed to evaluate outcomes associated with a context-dependent ayahuasca-assisted treatment intervention for substance use disorders. The protocol may also provide a usable framework for conducting studies with psychedelic-assisted therapy more generally. Applied within the context of a long-term residential treatment facility such as Takiwasi Center, the data yielded from this study will also offer insight into the therapeutic community treatment model for substance use disorders as implemented in a unique and complex environment.

## Footnotes

1 A maloca is a traditional longhouse often constructed of wood with a palm-thatched roof. In the Peruvian mestizo tradition, ayahuasca ceremonies are typically conducted in a maloca. This ‘rustic’ structure lends an ambience to the setting, demarcating it as a ‘safe’ ritual space. In Takiwasi, in particular, there are strict rules about how to exit and enter the maloca once the ceremony has begun.
