# Supplementary material for: Protocol for Outcome Evaluation of Ayahuasca-Assisted Addiction Treatment: The Case of Takiwasi Center
Source: Front Pharmacol. 2021 May 19;12:659644. doi: 10.3389/fphar.2021.659644 (PMC8170098; doi:10.3389/fphar.2021.659644)
Supplement: Supplementary file 1 [file Presentation1.PPTX]

## Slide 1
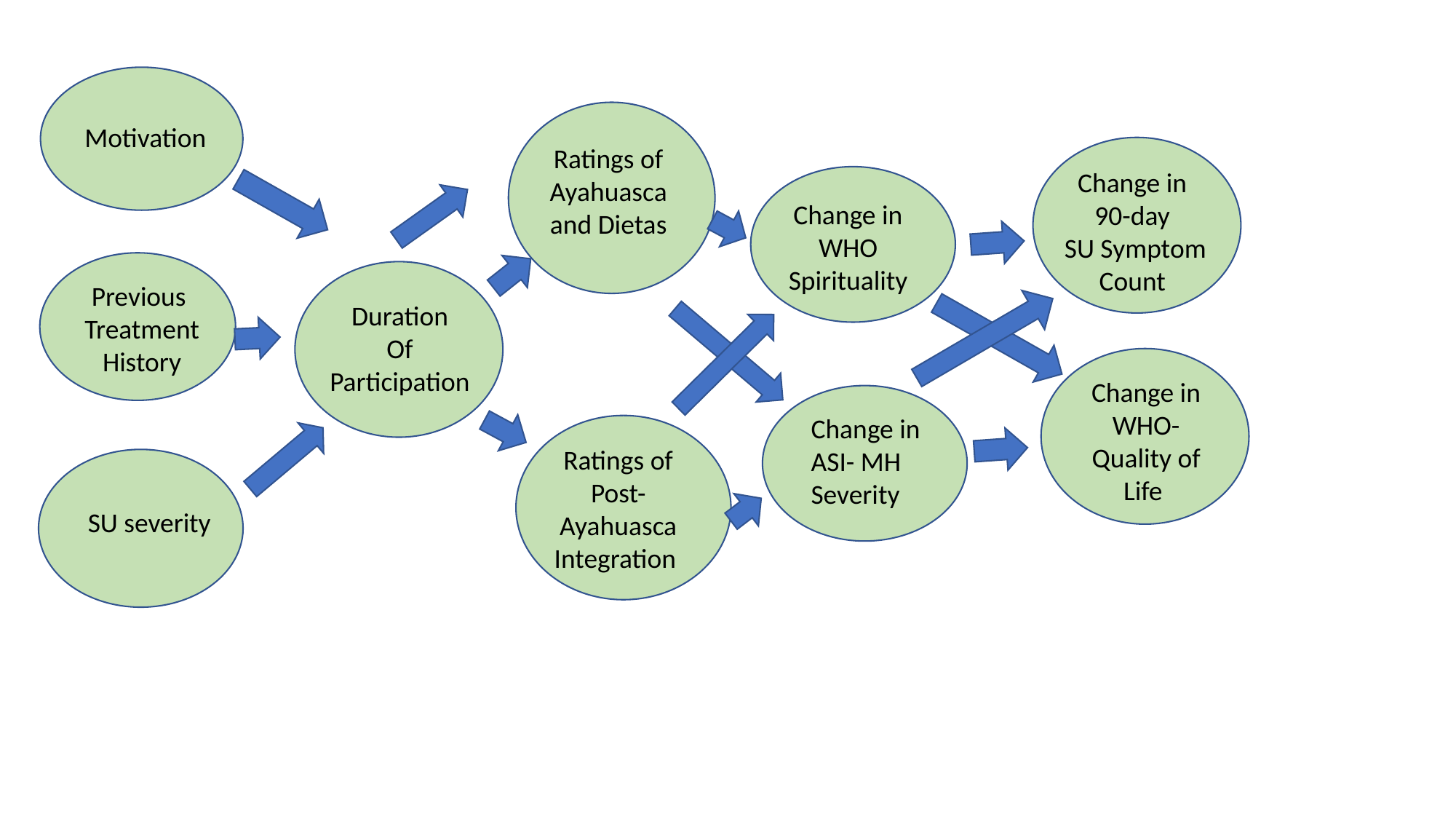

Motivation
Ratings of Ayahuasca and Dietas
Change in
90-day
SU Symptom Count
Change in WHO Spirituality
Previous
Treatment History
Duration
Of
Participation
Change in WHO-Quality of Life
Change in ASI- MH Severity
Ratings of Post-Ayahuasca Integration
SU severity
