# Supplementary material for: Protocol for Outcome Evaluation of Ayahuasca-Assisted Addiction Treatment: The Case of Takiwasi Center
Source: Front Pharmacol. 2021 May 19;12:659644. doi: 10.3389/fphar.2021.659644 (PMC8170098; doi:10.3389/fphar.2021.659644)
Supplement: Supplementary file 2 [file DataSheet1.PDF]

Supplementary Materials

Protocol for Outcome Evaluation of Ayahuasca-Assisted Addiction Treatment: The Case of Takiwasi Centre

Brian Rush

Olivia Marcus

Sara García

Anja Loizaga-Velder

Gabriel Loewinger

Ariane Spitalier

Fernando Mendive

This PDF file includes:

Tables S1 to S5
Annex 5.1. Baseline treatment interview with patients
Annex 5.2.1. During treatment and follow-up interview with patients
Annex 5.2.2. Short follow-up interview with patients
Annex 5.3. Takiwasi staff interview
Annex 5.4. Cultural Formulation Interview (Spanish version)
Annex 6. Package of Questionnaires
- Annex 6.1. MINI International Neuropsychiatric Interview (Spanish Version)
- Annex 6.2. Katzman Short Orientation, memory, concentration test (Spanish version)
- Annex 6.3. Baseline treatment questionnaire
- Annex 6.4. Addiction Severity Index (ASI) (Spanish version)
- Annex 6.5. GAIN (VGNI) (Spanish version)
- Annex 6.6. Beck Anxiety Inventory (Spanish version)
- Annex 6.7. Beck Depression Inventory (Spanish version)
- Annex 6.8. WHOQOL-BREF Quality of Life Assessment (Spanish Version)
- Annex 6.9. WHOQOL-BREF Spirituality, Religiousness and Personal Beliefs (Spanish version)
- Annex 6.10. Client Satisfaction Questionnaire (in Spanish)

Table S1. Baseline Sample Characteristics

<table>
<tr><th>Baseline Characteristics</th><th>Quantitative Indicator
N (%) or M (SD)</th><th>Qualitative Research Questions</th></tr>
<tr><td colspan="2">Recovery Capital</td><td rowspan="18">Patient characteristics: What are the background characteristics of Takiwasi clients?

Psychedelic/ayahuasca practices: What have been the previous experience(s) with psychedelic substances and ayahuasca specifically. What, if any, significant learnings and challenges from those experiences are being brought into this new experience at Takiwasi?

Religious and spiritual practices: What have been the previous experience(s) with religious or spiritual practices?

Treatment history: What has been the previous experience with substance use treatment and learning and challenges are being brought into this new experience at Takiwasi?

Problem awareness and understanding of addiction: What is the person’s subjective understanding of their addiction challenges and what is the match between person’s perspective and the treatment provided at Takiwasi?</td></tr>
<tr><td>Region</td><td></td></tr>
<tr><td>Age</td><td></td></tr>
<tr><td>Marital status</td><td></td></tr>
<tr><td>Living situation</td><td></td></tr>
<tr><td>Education</td><td></td></tr>
<tr><td>Religion</td><td></td></tr>
<tr><td>Employment status</td><td></td></tr>
<tr><td>ASI-Family</td><td></td></tr>
<tr><td>ASI Employment</td><td></td></tr>
<tr><td>ASI Legal</td><td></td></tr>
<tr><td>WHO – Quality of Life</td><td></td></tr>
<tr><td>WHO - Spirituality</td><td></td></tr>
<tr><td>Previous Use of Hallucinogens</td><td></td></tr>
<tr><td>Ayahuasca</td><td></td></tr>
<tr><td>Other</td><td></td></tr>
<tr><td>Previous Treatment</td><td></td></tr>
<tr><td>Residential</td><td></td></tr>
</table>

Non-residential		
**Treatment Motivation**		
Interoceptive		What are the *motivations, expectancies and worries* about the treatment experience ahead? Are these associated with treatment outcomes?
External		
Internal		
**Health and Mental Health Comorbidity**		
No. of diagnoses (last 12 months)		*Co-occurring mental and physical health challenges:* What is the current situation with respect to physical and mental health challenges and overall life satisfaction?
ASI Physical Health Index		
ASI MH Severity Index		
Beck Depression Inventory		
Beck Anxiety Inventory		*Substance use:* What has been the individual and family history with respect to substance use and related problems, including most recent challenges?
**Substance Use and Severity**		
Age of first use?		
Addiction Severity Index		
Alcohol		
Drugs		
GAIN SU Symptom count		
# days used in past 90 days		
# days high or drunk in past 90 days		
# days not meeting responsibilities		
# consecutive days use		

Table S2. Participation and Perceived Importance of Takiwasi Treatment Activities

**Takiwasi Treatment Activities**	**Quantitative Indicator** N (%) or M (SD)	**Qualitative Research Questions?**
**Participation in Treatment and Support Activities**		*Meanings/personal significance:* What meanings are attached to the overall Takiwasi treatment experience as well as key elements (e.g., treatment milieu, specific medicines, dieta, purging, icaros, therapeutic integration) and how have these meanings evolved during the course of the program?
Treatment duration (in weeks)		
# of Ayahuasca sessions		
# days of dieta		
# hours of counselling		
# hours of post-Ayahuasca integration		
# hours of other therapeutic activities (e.g., yoga, massage		*Program experience:* Do participants report satisfaction with the Takiwasi program and what aspects of the experience are perceived as being the most meaningful for achieving positive outcomes during the program and going forward post-discharge? What has been the most challenging aspect of your treatment?
**Perceived Importance of Program Activities**		
Group Therapy		
Individual therapy and counselling		
Work activities (garden, carpentry, kitchen etc)		
Community life		
Treatment with emetic plants (purges)		
Experience with Ayahuasca		
Ícaros (songs) in the rituals		
Cleansing or curing in the rituals		
Perfumes used in the rituals		
Planta de contención		

Follow up sessions about the ayahuasca experience		
**Perceptions of the experience with Ayahuasca**		
Significance of spiritual aspect of Ayahuasca experience (Rating Scale and comments)		
Contribute to changes in personal sense of wellbeing (Rating Scale and comments)		

Table S3. Changes over time in treatment outcome – data visualization and qualitative analysis

**Outcome Domain and Measurtes**	**Baseline** N (%) or M (SD)	**Discharge** N (%) or M (SD)	**3- mon.** N (%) or M (SD)	**6-mon.** N (%) or M (SD)	**12-mon.** N (%) or M (SD)	**Qualitative Research Questions**
						What is the relationship between treatment outcomes and baseline recovery capital?<br>• Patient demographic characteristics<br>• Previous psychedelic/ayahuasca practices<br>• Previous religious and spiritual practices<br>• Previous treatment history<br>• Problem awareness and understanding of addiction<br>*Recovery Process:* How to clients perceive their recovery process at different follow-up points? What outcomes are reported as being the most meaningful?
**Health and Social Functioning**						
ASI Composite – Health		NA				
ASI Composite – Empl.		NA				
ASI Composite – Legal		NA				
ASI Composite – Family		NA				
**Mental Health**						

ASI MH		NA				*Meanings/personal significance*: What aspects of the treatment experience are perceived as having been most important in achieving or maintaining these outcomes since discharge?<br><br>*Program experience:* Do participants report satisfaction with the Takiwasi program and what aspects of the experience are perceived as most meaningful for achieving positive outcomes since discharge?<br><br>*Post-treatment integration*: Do participants report incorporating elements of Takiwasi treatment protocol and their experiences into aspects of their life?
Beck Depression						
Beck Anxiety						
**Substance use and severity**						
ASI - Alcohol		NA				
ASI - Drugs		NA				
GAIN SU Symptom count – past three mon.		NA				
# days used in past 90 days		NA				
# days high or drunk in past 90 days		NA				
# days not meeting responsibilities due to alcohol or drug use		NA				
# consecutive days alcohol or drug use		NA				
**WHO measures**						
WHO- Quality of Life		NA				
WHO- Spirituality		NA				

Table S4. Explanatory modelling of changes over time in treatment outcome – Sample DAG for multivariate analysis and qualitative analysis

Sample DAG	Multivariate Model	Qualitative Research Question
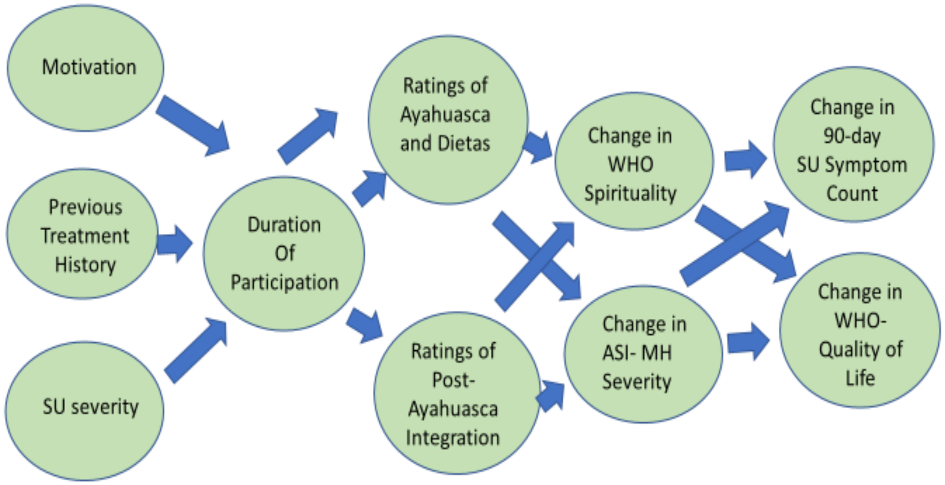		*Thematic analysis by sub-group based on outcomes measured quantitatively?*<br><br>After sub-dividing participants on the basis of "excellent", "good" and "poor" outcomes, what aspects of initial recovery capital, problem severity, the Takiwasi treatment experience, and short-term outcomes appear to be most associated with treatment outcome at 12-months?

Table S5. Context Analysis: Managers, Staff, Therapist and Healer Perspectives

Manager, Staff, Therapist and Healer Interviews	Qualitative Research Questions
	**Primary research question:** How homogenous is the therapeutic culture at Takiwasi? Sub-questions are identified below. Emergent themes will also be identified concerning the convergence and divergence of the therapeutic culture over time.<br><br>**Sub-questions guiding the analysis**<br><br>What are therapists' main theoretical training and has it influenced their work at Takiwasi? (*Aim*: To identify patterns in practical or theoretical background among practitioners who gravitate toward Takiwasi).<br><br>What are therapists' views on religion? How do their personal views relate to practices at Takiwasi or their relationships with clients? (*Aim*: To explore the subject of institutional religiosity, including the role of Christianity within the context of spirituality).<br><br>How has Takiwasi affected therapists' perceptions of care, psychology/psychotherapy, and practice? How did their time at Takiwasi influence their proximate career goals? (*Aim:* To explore the 'training' aspect of Takiwasi and how it influences practitioners).<br><br>What are the prevailing views and subjective understanding of addiction and related challenges? (Aim: to explore the match between practitioners and the treatment provided at Takiwasi).<br><br>Do practitioners link their own experiences with ceremonies and dietas with therapeutic efficacy and/or therapeutic alliance? (*Aim*: To determine the role of ayahuasca in developing a unique kind of therapeutic alliance).<br><br>Are there specific aspects of the ayahuasca experience (e.g., dietas, icaros, purging, cleansing/ limpiezas, visions, death experiences) that managers, staff and healers think are particularly helpful for healing? For alcohol and drug addiction specifically? (*Aim:* To explore potential links between prevailing views on program components and treatment outcomes).

ENTREVISTAS ATOP

ANEXO 5.1 Entrevista inicial de tratamiento con pacientes.

Fecha__________ Nombre del Entrevistador _______________
Lugar de la entrevista ___________________
Número de identificación del paciente en el estudio ___________________

Sólo para entrevista de "Durante el tratamiento":
Tiempo transcurrido desde la admisión________ Días____________
Fecha de la entrevista inicial___________ Nombre del entrevistador _______

Información personal del paciente

Nombre______________________________________ __________________

Fecha de nacimiento ___/___/_____
M D AÑO

Género M [ ] F [ ] Otro [ ]
Lugar de Nacimiento Ciudad __________________País ________________
Ciudad en la que creció __________________ País _____________________
Lugar de residencia Ciudad__________ País ______________
Ocupación/Profesión___________________________________
Empleo actual___________________
Bagaje Étnico/Cultural____
¿Vive su madre? si [ ] no [ ]
¿Vive su padre? si [ ] no [ ]
Usted vive con sus padres [ ] solo [ ] con su pareja [ ] con amigos [ ] otra[ ]
Si no vive con sus padres, en que año dejó la casa familiar? ______________

Guía para la entrevista
Por favor responda de acuerdo a lo que realmente siente y no de la manera que usted piensa que es mejor responder. No hay respuestas correctas o incorrectas.
Su información será tratada confidencialmente.
Su honestidad es importante para mejorar el tratamiento aquí y en otros Centros.

1.- Antecedentes con el uso de alcohol y drogas a lo largo de su vida
¿A qué edad fue su primer consumo de drogas/alcohol?
¿Cuáles fueron las primeras drogas qué ingirió?
¿Con qué frecuencia, cantidad y en qué contexto las consumía?
¿Cuándo surgieron los problemas vinculados al consumo?
¿Qué clase de problemas?
Desde su punto de vista; ¿Por qué surgieron estos problemas?

¿En su historia familiar, existen problemas de adicción a drogas y/o alcohol?

Indique cuáles sustancias ha consumido en los últimos 2 meses.
¿Alcohol/Drogas? ¿Cuáles? ¿Psicodélicos? ¿Marihuana? ¿Tabaco?
¿Qué cantidad usa diariamente?
¿En qué contexto las ha consumido? (lugares de consumo, personas con las que consume, situaciones por las cuales ha consumido, etc.)

Describa los problemas que ha tenido relacionados al consumo de estas sustancias.

2.- ¿Ha tenido intentos previos por reducir o dejar el uso de alcohol y/o drogas, así como de resolver los problemas relacionados con su uso?
¿Ha intentado hacerlo usted mismo sin tratamiento formal?
¿Con Alcohólicos Anónimos? ¿Con Narcóticos Anónimos?
¿Con un programa de tratamiento formal?
Si ha estado en otros tratamientos
¿Cuántos tratamientos anteriores ha hecho? ¿Qué tipo de tratamiento(s)?
¿Cuándo fue el último tratamiento? ¿Qué tipo de tratamiento? Cuál es el nombre del programa? ¿En qué lugar se ubica?
¿Cuáles son sus reflexiones sobre sus tratamientos pasados? ¿Qué lecciones aprendió? ¿Tuvo retos que enfrentar? ¿Qué le gustó? ,¿Que no le gustó? ¿Qué aprendió de esta(s) experiencias(s) que puedan ayudarle en el programa que está por comenzar?

3.- Experiencia personal en relación a la salud en general y a la salud mental
¿Cómo siente su estado de salud actualmente? ¿Padece o ha padecido alguna enfermedad crónica importante? ¿Actualmente recibe tratamiento para alguna enfermedad o un padecimiento mayor? ¿Reconoce alguna relación entre su condición de salud actual o pasada y el uso de drogas y/o alcohol?

¿Cómo siente su estado de salud mental actualmente? ¿Y en el pasado?
¿Se siente satisfecho con su vida actualmente? ¿Y en el pasado?

¿Ha experimentado alguno de los siguientes estados de salud mental en los últimos meses: desorden afectivo, ansiedad, traumas, síntomas psicóticos, ira, déficit de atención, hiperactividad? ¿Ha sido diagnosticado y/o tratado por alguno de estos estados? ¿Está siendo tratado actualmente por alguna de estas condiciones? ¿Cuál/Cuales?
¿Reconoce alguna conexión entre estos estados mentales (actuales o del pasado) y el uso de drogas y/o alcohol?

4.-Motivación y expectativas para el programa que va iniciar
¿Por qué decidió venir a Takiwasi?
¿En general? ¿Vino por recomendación? ¿Quién o en dónde lo recomendaron?
¿Le parece que este tratamiento es más innovador? ¿Le parece que cuenta con un acercamiento más espiritual?
¿Le interesa específicamente el trabajo con la Ayahuasca y otras plantas medicinales?

¿Cuáles son sus expectativas? – ¿Qué aspectos del tratamiento aquí espera que puedan ayudarle a usted?
Tiene preocupaciones y/o temores sobre el tratamiento que está por comenzar? ¿De qué se tratan dichas preocupaciones o temores?

5.-Experiencia previa con Ayahuasca
¿Ha tenido experiencias previas con Ayahuasca?
¿Cuándo? y en qué contexto se dio?
Algunos ejemplos son: En un contexto religioso (Santo Daime, UDV, Barquinha, otra); en la Tradición Amazónica ¿En alguna ceremonia donde mezclaron diferentes plantas o diferentes formas de rituales?, en sesiones terapéuticas individuales, en espacios sin ningún contexto ceremonial, otra.
¿Cuántas veces?
¿En qué lugares?
¿Tuvo alguna experiencia significativa, aprendió alguna lección, o tuvo alguna revelación en este contexto que pueda ser de valor para usted en el programa que está por iniciar?

6.*-Experiencia previa con psicodélicos*
¿Ha tenido experiencias previas con sustancias psicodélicas?
Por ejemplo hongos psilocibina, LSD, DMT, extasy
¿Cuántas veces?
¿En qué contexto? (para uso recreativo solamente, en alguna ceremonia donde mezclaron diferentes sustancias? en sesiones de terapia guiada, sin ningún contexto ceremonial, otra.
¿Tuvo alguna experiencia significativa, aprendió alguna lección, o tuvo alguna revelación en este contexto que pueda ser de valor para usted en el programa que está por iniciar?

7.- Experiencia previa con prácticas de tipo religioso o espiritual
¿Participa en alguna organización religiosa? ¿Qué religión es? (Católica, Pentecostés, Budismo, otra) ¿Cuál es su grado de participación?
¿Participa de otros tipos de prácticas espirituales tales como la meditación, el yoga u otras?
¿Cuál es su grado de participación?
¿Tuvo alguna experiencia significativa, aprendió alguna lección, o tuvo alguna revelación en este contexto que pueda ser de valor para usted en el programa que está por iniciar?

8.-¿Hay algo más que le gustaría añadir que crea que puede ayudarnos a entender su situación en relación al tratamiento que va iniciar?

ANEXO 5.2.1 Entrevista durante y de seguimiento del tratamiento con pacientes.

Fecha__________ Nombre del Entrevistador _______________
Lugar de la entrevista ___________________
Número de identificación del paciente en el estudio ___________________
Tiempo transcurrido desde la admisión________ Días____________
Fecha de la entrevista inicial___________ Nombre del entrevistador _______

Información personal del Paciente

Nombre______________________________________ __________________

Guía para la entrevista
Por favor responda de acuerdo a lo que realmente siente y no de la manera que usted piensa que es mejor responder. No hay respuestas correctas o incorrectas.
Su información será tratada confidencialmente.
Su honestidad es importante para mejorar el tratamiento aquí y en otros Centros.

1.- Apreciaciones generales de su experiencia con el tratamiento en Takiwasi.
¿Cuál es su apreciación general de la experiencia en el tratamiento?
¿Desde cuándo participa (o cuánto participó) en el programa?
¿Cuáles son los s aspectos del tratamiento que le han sido de ayuda hasta el momento?
¿Cuál ha sido el aspecto de mayor ayuda?
¿Cuáles han sido los aspectos de menos ayuda? ¿Qué ha sido lo más difícil de enfrentar?

Preguntar sólo si es entrevista de seguimiento:
¿Terminó el programa? Si la respuesta es no, indique porque.

2.- Apreciaciones generales de la experiencia del tratamiento con Ayahuasca
¿Participó en sesiones con Ayahuasca? Si la respuesta es no, explique por qué.
¿Cómo está siendo (o cómo fue) su experiencia en relación con sus expectativas, temores o preocupaciones al inicio del tratamiento?
¿Qué aspectos del tratamiento en relación con la Ayahuasca que le han sido particularmente de ayuda?
¿Qué aspectos no le han sido de ayuda?
Sintió que estaba preparado para la primera experiencia con Ayahuasca? Si la respuesta es afirmativa, explique de qué manera? Si es negativa, explique porque?
¿Cuántas veces participo de sesiones de Ayahuasca durante el programa?
¿Qué aprendió durante esta(s) experiencia(s) que pueda ayudarle durante su tratamiento, o en el futuro (si es entrevista de seguimiento)?

3.- Descripción de la(s) experiencia(s) más importantes con Ayahuasca Narración abierta de sus experiencias (siempre y cuando sean diferentes de las que ha reportado en entrevistas anteriores).

¿De qué se trataron esta(s) experiencia(s) que fue/ron tan importante/s o de ayuda para usted?
¿Le fue posible hacer una conexión entre esta(s) experiencia(s) y su consumo de alcohol u otras drogas? Explique en qué consistieron estas conexiones ¿Pudo comprender algunas de las razones o motivos que lo llevaron al consumo? ¿Pudo comprender con mayor claridad las consecuencias de su consumo? ¿Ha encontrado alguna orientación sobre cómo cambiar su situación?
Si ha tenido más de una experiencia con Ayahuasca: ¿Ha podido ver o aprender algo en una sesión que podía relacionar o integrar con experiencias de sesiones posteriores?

Si ha hecho tratamientos previos por alcohol o drogas: ¿Ha podido aprovechar alguna de estas experiencias de otros tratamientos y profundizar en ellas gracias a la(s) sesión(es) con Ayahuasca?

4.- Integración de experiencias con Ayahuasca
Apreciación del acompañamiento ofrecido para interpretar e integrar las experiencias durante las sesiones de Ayahuasca.

¿Le ofrecieron acompañamiento para interpretar o integrar su experiencia y lo que pudo haber aprendido durante las sesiones de Ayahuasca?
¿Quién le ofreció acompañamiento ? ¿Miembros del equipo, otros pacientes?
¿Qué tipo de acompañamiento recibió?
¿Le resultó de ayuda? ¿Fue perjudicial o le resulto difícil?

5.- ¿En el aspecto espiritual, cuán significativa ha sido su experiencia con la Ayahuasca?

1 Nada espiritual
2 Poco espiritual
3 Moderadamente espiritual
4 Muy espiritual
5 La espiritual más significativa experiencia en mi vida
0 No lo sé todavía, – es muy pronto para decirlo.

Narre su experiencia al respecto

6.- Cree usted que la experiencia con Ayahuasca, y su apreciación de esa experiencia, han dado lugar a cambios en su sentido de bienestar personal o satisfacción de su vida?

1 Ha disminuido mucho
2 Ha disminuido algo
3 Es la misma

4 Ha aumentado un poco
5 Ha aumentado mucho
0 No se puede decir realmente – es muy pronto para decirlo
Comentarios acerca de su respuesta:

7.- Indicaciones específicas sobre los elementos del tratamiento asistido con Ayahuasca

En relación con su tratamiento para su problema con las drogas o el alcohol, cuán útiles han sido los siguientes elementos terapéuticos?
Entrevistador: si el paciente no ha experimentado ningún aspecto en particular o si los elementos no son parte del programa, marque "no aplicable".

	No del todo útil 1	Algo útil 2	Moderada-mente útil 3	Muy útil 4	No aplicable 0
Terapia grupal					
Terapia individual o consejería					
Actividades de trabajo (jardinería, carpintería, cocina etc.)					
La vida en comunidad					
Las purgas (tratamiento con plantas eméticas)					
La experiencia con Ayahuasca					
Los Ícaros (cantos) en los rituales					
Las "Limpias" o curaciones en los rituales					
Fragancias usadas en los rituales					
Las dietas					
Planta de contención					
Las pláticas posteriores sobre la experiencia con Ayahuasca					
Otra (especifique por favor)					

*Otra: Purgahuasca, ritual de tierra, de agua, temazcal, ritual del niño no nacido, ritual de quema de máscaras, misa de reconciliación, misa del árbol genealógico.

7b).- Hasta el momento, ¿cuáles son los dos elementos terapéuticos de mayor ayuda?
1:
2:
Porque los considera los de mayor ayuda?

7c).- ¿Cuál/es elemento/s durante el tratamiento ha/n sido el/los de menos ayuda?
1:
2:
Porque considera que son los de menos ayuda?

7d.- El tratamiento con Ayahuasca, ha tenido algún efecto sobre su deseo o compulsión por la droga? Por favor describa lo que usted siente al respecto en esta etapa del tratamiento.

7e.- ¿Qué opina sobre de la frecuencia con la que se llevan a cabo las sesiones de Ayahuasca durante el tratamiento? ¿Considera que debería haber más o menos sesiones de Ayahuasca? Explique por qué. Si es entrevista de "durante el tratamiento": ¿El número de sesiones ha sido bueno hasta ahora?. ¿Por qué?, ¿Considera adecuado el espaciamiento entre sesiones/ingestas de Ayahuasca?

7f.- ¿Cree que la Ayahuasca puede ser adictiva?, ¿Cree que puede ser adictiva para la gente en general?, ¿Y para usted específicamente?

7g.- ¿Cree que la Ayahuasca puede ser perjudicial de alguna manera? ¿De qué manera cree que puede serlo? ¿Cree que puede ser perjudicial para la gente en general?, ¿Y para usted específicamente?

8.- Satisfacción con los servicios en el tratamiento asistido con Ayahuasca.

Pregunta 8a, sólo si es entrevista de seguimiento:
8a.-En términos generales ¿Cómo se encuentra actualmente con respecto a su consumo de alcohol y drogas?
¿Ha habido cambios significativos o importantes en su vida? Si la respuesta es sí, narre brevemente cuáles han sido.
¿Cuáles son los factores más importantes que relaciona con estos cambios? ¿Porque sigue usted con dificultades para dejar el alcohol o las drogas?
¿Cuáles considera que han sido los factores más importantes en su recuperación desde que terminó el programa?

8b.- **Satisfacción con el tratamiento para "entrevista de durante" y "de seguimiento"**

	Totalmente en desacuerdo 1	En desacuerdo 2	De acuerdo 3	Totalmente de acuerdo 4

El tratamiento recibido me ha ayudado a enfrentar más efectivamente las dificultades de la vida				
Pienso que los servicios en este lugar son de alta calidad				
Si un amigo necesitara ayuda para rehabilitarse por adicción, le recomendaría el tratamiento en este Centro				

8c.-Por favor compártanos cualquier comentario que nos ayude a mejorar los servicios en el Centro.

9.- Por favor añada cualquier comentario o sugerencia que considere relevante en relación a la superación de su problema de drogas o alcohol con apoyo de ayahuasca y acerca de su estancia en el Centro.

ANEXO 5.2.2 Entrevista reducida de seguimiento con pacientes.

Nombre del participante______________________
Número de identificación del participante___________________
Fecha__________
Tiempo transcurrido desde que finalizó el tratamiento________
Entrevista número _____
Nombre del Entrevistador _______________

----

1. Estado actual y calidad de vida

¿Cómo te encuentras?

¿Ha habido cambios importantes en tu vida en este tiempo? Si es que sí, ¿cuáles? ¿Cómo han impactado en tu calidad de vida?

2. Consumo de drogas

¿Has tenido deseo de consumir? Si es que sí, ¿cómo manejaste ese deseo?

¿Has consumido drogas/alcohol desde la última entrevista?

- Si es que sí, por favor comenta en qué contexto y por qué crees que consumiste. ¿Cómo te sentiste después de consumir?
- Si es que no, ¿qué factores te han ayudado a no consumir?

3. Apreciaciones generales del tratamiento

¿Hasta ahora, cuál es tu apreciación general del tratamiento? ¿Ha cambiado en algo desde la última entrevista? ¿ En qué? Comenta con detalle.

¿ Cuales fueron tus aprendizajes y experiencias más importantes durante el tratamiento? ¿Desde que terminaste el tratamiento, han cambiado tus impresiones sobre cuales fueron las experiencias y aprendizajes más importantes?

¿ Han cambiado tus impresiones sobre las experiencias con ayahuasca desde la última entrevista? ¿ En qué? Comenta con detalle.

4. Sugerencias

Por favor comparta alguna sugerencia para mejorar el tratamiento.

ANEXO5.3.Entrevista con el personal deTakiwasi.

Entrevista semi-estructurada con el personal, dirigentes y curanderos de los centros

Fecha__________ Nombre del Entrevistador _______________
Lugar de la entrevista ___________________

Información personal e historia de vida

Nombre ___________________

Fecha de nacimiento ___/___/_____
M D AÑO

Género M [ ] F [ ] Otro [ ]

Lugar de nacimiento: Ciudad/pueblo __________________País___________________

Domicilio actual __________________________________

Idioma (s) __________________________________

Áreas temáticas

A.-Sobre el papel que desempeña en el Centro
¿Cuál es su cargo en el Centro? ¿Cuáles son sus funciones y responsabilidades? ¿Qué funciones informales también cubre?

B.- Formación profesional (Sobre el entrenamiento para trabajar con Ayahuasca, ver más adelante en la sección E)

1) Educación formal, disciplina y grado de estudios.
2) Referente a la formación para el tratamiento de la adicción al alcohol y otras drogas – ¿Tiene algún tipo de entrenamiento o formación profesional en este ámbito? ¿Dónde se formó? ¿Con quién? ¿Bajo qué enfoque terapéutico? ¿Cuántos años de experiencia tiene al respecto?
3) ¿Cuál es su formación en el ámbito de la salud mental? ¿Cuál es su experiencia de trabajo con problemas de salud mental tales como la depresión, ansiedad, psicosis, entre otros? ¿Cuáles aspectos de las formaciones le resultaron más útiles en su trabajo en el Centro? ¿Cuáles no le resultaron útiles? ¿Cuenta con entrenamiento para hacer intervenciones en el manejo de crisis? ¿Cuenta con entrenamiento para hacer evaluación de riesgo suicida?
4) ¿Cuenta con alguna formación en medicina? (por ejemplo: medicina alopática, enfermería, fisioterapia, otra)
5) ¿Cuenta con alguna formación en sistemas o técnicas de medicina complementaria? (por ejemplo: yoga, reiki, acupuntura, masaje, medicina china, osteopatía, quiropraxia, otra)
6) ¿Cuenta con alguna formación en Medicina tradicional indígena? (por ejemplo: uso de kambó, tabaco, santa maría, peyote, san pedro, hongo psilocibe).
(Si tiene formación como Ayahuasquero/a, por favor, responda en el inciso "F")
Para cada una de estas prácticas de Medicina Indígena, indique:
¿Cuántos años de entrenamiento tiene?
¿Dónde recibió su entrenamiento?
¿Con quién aprendió?

C.- Religión y/o prácticas espirituales personales
1) ¿Participa de alguna organización religiosa? ¿En cuál? (Iglesias Católica, Protestante, Pentecostés, Budista, Santo Daime, otra) ¿Cuál es su grado de participación?
2) ¿Se identifica o pertenece a alguna tradición religiosa? ¿Con cuál? (Católica, Protestante, Pentecostés, Budista, otra). ¿Participa activamente del culto?
3) ¿Practica otros tipos de ejercicios espirituales como la meditación, yoga u otras?. ¿Cuál es su grado de participación?

D.-Consumo personal de sustancias y su opinión acerca del tratamiento de drogas y alcohol.
1) ¿Ha consumido alcohol u otras drogas? ¿Continúa usándolas?
2) ¿Porque comenzó a consumirlas? ¿Cuáles fueron sus razones para usarlas? ¿Ha tenido problemas relacionados a su consumo? ej. problemas personales/familiares/de trabajo o de dependencia
3) ¿Qué droga/s ha consumido? ¿Cuál de estas prefiere? ¿Por qué?
4) ¿Ha estado en tratamiento por drogas y/o alcohol? Si su respuesta es afirmativa, ¿En qué lugar lo ha hecho? ¿Qué tipo de tratamiento ha sido? ¿Qué fue lo más benéfico del tratamiento? ¿Qué fue lo menos benéfico?

E.- Identificación y caracterización de lo que es un uso problemático de substancias o drogas por las personas que conducen las sesiones de ayahuasca.
1) ¿Qué es lo que usted considera un uso problemático de drogas?
2) ¿Cómo define la adicción/dependencia? ¿Cuáles considera que son las causas de esta enfermedad?
3) ¿Cómo define la recuperación de una adicción? ¿Cómo considera que se da este proceso de recuperación? ¿Cuáles considera que son los aspectos más críticos en un proceso de recuperación de una adicción?

F.- Experiencia trabajando con Ayahuasca

1) ¿Tiene experiencia previa con pacientes adictos a alcohol y drogas usando otros métodos de tratamiento? ¿Qué aspectos de estos otros métodos de tratamiento considera útiles? ¿Cuáles no considera útiles? ¿Por qué?

2) ¿Por qué decidió trabajar con Ayahuasca para ayudar a otros a sanar?

3) ¿Cuánto tiempo ha trabajado con Ayahuasca específicamente? ¿Dónde aprendió? ¿Con quién? ¿En qué tradición?

4) ¿En general, cuál es su experiencia trabajando bajo este enfoque en término de riesgos y beneficios, aprendizajes, enseñanzas recibidas a lo largo de los años?) ¿Desde su experiencia, qué aspectos del trabajo con Ayahuasca son los más beneficiosos? ¿Ha observado algún tipo de riesgo para los pacientes?

G. obre su experiencia con Ayahuasca

1) áblenos de su primera experiencia personal con Ayahuasca.

2) áblenos de la experiencia más importante para usted. ¿Por qué fue importante?

3) áblenos acerca de alguna de las más difíciles? ¿Por qué fue difícil?

H) Experiencias durante sesiones de ayahuasca con pacientes que puedan ser conectadas con cambios de hábitos o comportamiento, en relación con el uso de drogas (ícaros, vómito, curaciones/limpia, visiones)

1) ¿Como ayuda la experiencia con Ayahuasca en el proceso de recuperación de adicciones?

2) ¿Considera que la Ayahuasca puede ser perjudicial de alguna manera? ¿Considera que la Ayahuasca puede ser adictiva?

3) ¿Qué aspectos en la experiencia con Ayahuasca considere particularmente útiles para sanar? (ej. dietas, ícaros, purgas, limpias, visiones, experiencias de muerte) ¿Y específicamente en el caso de las adicciones?

4) Sobre la relación que puede haber entre el trabajo con Ayahuasca y otro tipo de rituales (ej., kambó, tabaco, santa María, peyote, san Pedro, hongos psilocibe). ¿Considera de utilidad el uso de otras plantas medicinales en combinación con la Ayahuasca? ¿Cuáles y por qué? ¿Sabe de algunas plantas que no deben ser usadas en combinación con Ayahuasca? ¿Por qué no?

I) Sobre el uso de terapias complementarias en sesiones de Ayahuasca, y su utilidad para generar cambios de hábitos o comportamiento en relación con el uso de alcohol y drogas.

1) ¿Conoce técnicas terapéuticas que se usen en combinación con las sesiones de Ayahuasca, y que considere sean de utilidad? (por ejemplo arte terapia, ergoterapia, prácticas contemplativas, entrenamiento en habilidades sociales, rehabilitación psicosocial (como orientación sobre educación y empleo), dietas, purgas y trabajo con otros plantas medicinales).

2) ¿Cuáles de estas técnicas terapéuticas considera son de mayor utilidad para para la integración de las enseñanzas y/o aprendizajes vividos en la sesión de Ayahuasca en la vida cotidiana? ?

J.- Por favor comente acerca de cualquier tema que considere relevante para el proceso de rehabilitación de adicciones que no haya sido cubierto en esta entrevista.

ANEXO 5.4. Cultural Formulation Interview.

Entendimiento general del problema

1. Podría decirme cómo entiende usted el (/su) problema de adicción a las drogas/alcohol?
2. Qué sabía usted sobre el (/su) problema de adicción a las drogas/alcohol antes de que le afectara?

Estereotipos sobre la enfermedad

3. Ha pasado usted por un problema parecido a la adicción a las drogas/alcohol antes? Por favor, describa.
4. Conoce a alguien o ha oído hablar de alguien con este problema? Si es así, por favor describa el problema de adicción de esta persona y cómo le afectó. Cree que esto podría pasarle a usted?
5. Ha visto en la TV, ha oído en la radio, leído en el periódico, o encontrado en internet algo relacionado con el (/su) problema de adicción a las drogas/alcohol? Por favor describa.

Explicaciones causales

6. Puede decirme que piensa usted sobre que causa el (/su) problema de adicción a las drogas/alcohol? Hay más de una razón que explique su problema?
7. Han cambiado sus ideas respecto a lo que causa el (/su) problema de adicción a las drogas/alcohol? ¿Qué factores han contribuido a cambiar sus ideas respecto a lo que causa el problema?
8.¿Qué piensa su familia, sus amigos u otros en su comunidad sobre que causa el problema de la adicción a las drogas/alcohol?
9. Cómo creé que el (/su) problema de adicción a las drogas/alcohol afecta al (/su) cuerpo, mente y bienestar espiritual?

Curso de la enfermedad

10. Qué suele pasarle a la gente que tiene problemas con las drogas/alcohol? En tu propio caso, que crées que sucederá?
11. Considera que el (/su) problema de adicción a las drogas/el alcohol es serio? Por qué? Qué es lo peor que podría ocurrir?
12. Cuán preocupados están su familia, amigos y otros miembros de su comunidad sobre su (/el) problema de adicción a las drogas/alcohol? Por favor describa.

Búsqueda de tratamiento y expectativas

13. Cuál crees que es la mejor manera de tratar el problema de la adicción a las drogas/alcohol?
14. Cual creen sus familiares/amigos/u otros de su comunidad que es la mejor manera de tratar el problema de la adicción a las drogas/alcohol?

ANEXO 6. Medidas Cuantitativas.

Lista de medidas cuantitativas incluidas en las próximas páginas:

ANEXO 6.1. La entrevista neuropsiquiátrica internacional MINI.

ANEXO 6.2. EL test breve de orientación, memoria y concentración de Katzman.

ANEXO 6.3. Cuestionario de ingreso al tratamiento

ANEXO 6.4. Índice de la Severidad de la Adicción ASI

ANEXO 6.5. GAIN I (VGNI)

ANEXO 6.6. Inventario de ansiedad de Beck

ANEXO 6.7. Inventario de depresión de Beck

ANEXO 6.8. Escala de calidad de vida WHOQOL-BREF

ANEXO 6.9. Escala de espiritualidad WHOQOL-SRPB

ANEXO 6.10. Cuestionario de satisfacción del cliente *(CSQ-8)*

1.1. MINI Entrevista Neuropsiquiátrica Internacional (MINI International Neuropsychiatric Interview, MINI)

MINI

MINI INTERNATIONAL NEUROPSYCHIATRIC INTERVIEW

Versión en Español 5.0.0
DSM-IV

EE.UU.: **D. Sheehan, J. Janavs, R. Baker, K. Harnett-Sheehan, E. Knapp, M. Sheehan**
University of South Florida, Tampa

FRANCIA: **Y. Lecrubier, E. Weiller, T. Hergueta, P. Amorim, L. I. Bonora, J. P. Lépine**
Hôpital de la Salpétrière, Paris

Versión en español:

L. Ferrando, J. Bobes, J. Gibert
Instituto IAP – Madrid – España

M. Soto, O. Soto
University of South Florida, Tampa
Asesores de traducción: L. Franco-Alfonso, L. Franco

© Copyright 1992, 1994, 1998, 1999 Sheehan DV & Lecrubier Y.

Todos los derechos están reservados. Ninguna parte de este documento puede ser reproducida o transmitida en forma alguna, ni por cualquier medio electrónico o mecánico, incluyendo fotocopias y sistemas informáticos, sin previa autorización escrita de los autores. Investigadores y clínicos que trabajen en instituciones públicas o lugares no lucrativos (incluyendo universidades, hospitales no lucrativos e instituciones gubernamentales) pueden hacer copias del MINI para su uso personal.

MINI 5.0.0 (1 de enero de 2000)

Nombre del paciente: ______________________
Fecha de nacimiento: ______________________
Nombre del entrevistador: ______________________
Fecha de la entrevista: ______________________
Número de protocolo: ______________________
Hora en que inició la entrevista: ______________________
Hora en que terminó la entrevista: ______________________
Duración total: ______________________

	Módulos	*Período explorado*	*Cumple los criterios*	*DSM-IV*	*CIE-10*
A	EPISODIO DEPRESIVO MAYOR (EDM)	Actual (2 semanas)	☐	299.20-296.26 episodio único	F32.x
		Recidivante	☐	296.30-296.36 recidivante	F33.x
	EDM CON SÍNTOMAS MELANCÓLICOS (opcional)	Actual (2 semanas)	☐	296.20-296.26 episodio único<br>296.30-296.36 recidivante	F32.x<br>F33.x
B	TRASTORNO DISTÍMICO	Actual (últimos 2 años)	☐		
C	RIESGO DE SUICIDIO	Actual (último mes)	☐	300.4	F34.1
		Riesgo:<br>☐ leve ☐ moderado ☐ alto	☐		
D	EPISODIO MANÍACO	Actual	☐	296.00-296.06	F30.x-F31.9
		Pasado	☐		
	EPISODIO HIPOMANÍACO	Actual	☐	296.80-296.89	F31.8-F31.9/F34.0
		Pasado	☐	300.01/300.21	F40.01-F41.0
E	TRASTORNO DE ANGUSTIA	Actual (último mes)	☐		
		De por vida	☐	300.22	F40.00
F	AGORAFOBIA	Actual	☐		
G	FOBIA SOCIAL *(Trastorno de ansiedad social)*	Actual (último mes)	☐	300.23	F40.1
H	TRASTORNO OBSESIVO-COMPULSIVO	Actual (último mes)	☐	300.3	F42.8
I	ESTADO POR ESTRÉS POSTRAUMÁTICO (opcional)	Actual (último mes)	☐	309.81	F43.1
J	DEPENDENCIA DE ALCOHOL	Últimos 12 meses	☐	303.9	F10.2x
	ABUSO DE ALCOHOL	Últimos 12 meses	☐	305.00	F10.1
K	DEPENDENCIA DE SUSTANCIAS (no alcohol)	Últimos 12 meses	☐	304.00-.90/305.20-.90	F11.1-F19.1
	ABUSO DE SUSTANCIAS (no alcohol)	Últimos 12 meses	☐	304.00-.90/305.20-.90	F11.1-F19.1
L	TRASTORNOS PSICÓTICOS	De por vida	☐	295.10-295.90/297.1	F20.xx-F29
		Actual	☐	297.3/293.81/293.82<br>293.89/298.8/298.9	
	TRASTORNO DEL ESTADO DEL ÁNIMO CON SÍNTOMAS PSICÓTICOS	Actual	☐	296.24	F32.3/F33.3
M	ANOREXIA NERVIOSA	Actual (últimos 3 meses)	☐	307.1	F50.0
N	BULIMIA NERVIOSA	Actual (últimos 3 meses)	☐	307.51	F50.2
	ANOREXIA NERVIOSA TIPO COMPULSIVA/PURGATIVA	Actual	☐	307.1	F50.0
O	TRASTORNO DE ANSIEDAD GENERALIZADA	Actual (últimos 6 meses)	☐	300.02	F41.1
P	TRASTORNO ANTISOCIAL DE LA PERSONALIDAD (opcional)	De por vida	☐	301.7	F60.2

MINI 5.0.0 (1 de enero de 2000)

1.1. MINI Entrevista Neuropsiquiátrica Internacional (MINI International Neuropsychiatric Interview, MINI)

Instrucciones generales

La MINI es una entrevista diagnóstica estructurada de breve duración que explora los principales trastornos psiquiátricos del Eje I del DSM-IV y la CIE-10. Estudios de validez y de confiabilidad se han realizado comparando la MINI con el SCID-P para el DSM-III-R y el CIDI (una entrevista estructurada desarrollada por la Organización Mundial de la Salud para entrevistadores no clínicos para la CIE-10). Los resultados de estos estudios demuestran que la MINI tiene una puntuación de validez y confiabilidad aceptablemente alta, pero puede ser administrada en un período de tiempo mucho más breve (promedio de 18,7 ± 11,6 minutos, media 15 minutos) que los instrumentos mencionados. Puede ser utilizada por clínicos tras una breve sesión de entrenamiento. Entrevistadores no clínicos deben recibir un entrenamiento más intenso.

ENTREVISTA:
Con el fin de hacer la entrevista lo más breve posible, informe al paciente que va a realizar una entrevista clínica que es más estructurada de lo usual, en la cual se le van a hacer unas preguntas precisas sobre sus problemas psicológicos y las cuales requieren unas respuestas de sí o no.

PRESENTACIÓN:
La MINI está dividida en módulos identificados por letras, cada uno corresponde a una categoría diagnóstica.

- Al comienzo de cada módulo (con excepción del módulo de los trastornos psicóticos), se presentan en un **recuadro gris**, una o varias preguntas «**filtro**» correspondientes a los criterios diagnósticos principales del trastorno.
- Al final de cada módulo, una o varias casillas diagnósticas permiten al clínico indicar si se cumplen los criterios diagnósticos.

CONVENIOS:
Las oraciones escritas en «letra normal» deben leerse «palabra por palabra» al paciente con el objetivo de regularizar la evaluación de los criterios diagnósticos.

Las oraciones escritas en «MAYÚSCULAS» no deben de leerse al paciente. Éstas son las instrucciones para asistir al entrevistador a calificar los algoritmos diagnósticos.

Las oraciones escritas en «**negrita**» indican el período de tiempo que se explora. El entrevistador debe leerlas tantas veces como sea necesario. Sólo aquellos síntomas que ocurrieron durante el período de tiempo explorado deben ser considerados al codificar las respuestas.

Respuestas con una flecha encima (➡) indican que no se cumple uno de los criterios necesarios para el diagnóstico. En este caso el entrevistador debe pasar directamente al final del módulo, rodear con un círculo «**NO**» en todas las casillas diagnósticas y continuar con el siguiente módulo.

Cuando los términos están separados por una barra (/) el entrevistador debe leer sólo aquellos síntomas que presenta el paciente (p. ej., la pregunta H6).

Frases entre paréntesis () son ejemplos clínicos de los síntomas evaluados. Pueden leerse para aclarar la pregunta.

INSTRUCCIONES DE ANOTACIÓN:
Todas las preguntas deben ser codificadas. La anotación se hace a la derecha de la pregunta enmarcando SÍ o NO.

El clínico debe asegurarse de que cada dimensión de la pregunta ha sido tomada en cuenta por el paciente (p. ej., período de tiempo, frecuencia, severidad, alternativas y/o).

<u>Los síntomas que son mejor explicados por una causa médica o por el uso de alcohol o drogas no deben codificarse sí en la MINI.</u> La MINI Plus tiene preguntas que explora estos problemas.

Para preguntas, sugerencias, sesiones de entrenamiento o información acerca de los últimos cambios en la MINI se puede comunicar con:

David V Sheehan, M.D., M.B.A.
University of South Florida
Institute for Research in Psychiatry
3515 East Fletcher Avenue
Tampa, FL USA 33613-4788
Tel.: + 1 813 974 4544
Fax: + 1 813 974 4575
e-mail: dsheehan@hsc.usf.edu

Yves Lecrubier, M.D./Thierry Hergueta, M.S.
INSERM U302
Hôpital de la Salpétrière
47, boulevard de l’Hôpital
F. 75651 París, Francia
Tel.: + 33 (0) 1 42 16 16 59
Fax: + 33 (0) 1 45 85 28 00
e-mail: hergueta@ext.jussieu.fr

Laura Ferrando, M.D.
IAP
Velázquez, 156,
28002 Madrid, España
Tel.: + 91 564 47 18
Fax: + 91 411 54 32
e-mail: iap@lander.es

Marelli Soto, M.D.
University of South Florida
3515 East Fletcher Avenue
Tampa, FL USA 33613-4788
Tel.: + 1 813 974 4544
Fax: + 1 813 974 4575
e-mail: mon0619@aol.com

MINI 5.0.0 (1 de enero de 2000)

A. Episodio depresivo mayor

(➡ SIGNIFICA: IR A LAS CASILLAS DIAGNÓSTICAS, RODEAR CON UN CÍRCULO NO EN CADA UNA Y CONTINUAR CON EL SIGUIENTE MÓDULO)

A1		¿En las últimas 2 semanas, se ha sentido deprimido o decaído la mayor parte del día, casi todos los días?	NO	SÍ	1
A2		¿En las últimas 2 semanas, ha perdido el interés en la mayoría de las cosas o ha disfrutado menos de las cosas que usualmente le agradaban?	NO	SÍ	2
		¿CODIFICÓ **SÍ** EN **A1** O EN **A2**?	➡ NO	SÍ	
A3		**En las últimas 2 semanas, cuando se sentía deprimido o sin interés en las cosas:**			
	a	¿Disminuyó o aumentó su apetito casi todos los días? ¿Perdió o ganó peso sin intentarlo (p. ej., variaciones en el último mes de ± 5 % de su peso corporal o ± 8 libras o ± 3,5 kg, para una persona de 160 libras/70 kg)?<br>CODIFICAR **SÍ**, SI CONTESTÓ **SÍ** EN ALGUNA	NO	SÍ	3
	b	¿Tenía dificultad para dormir casi todas las noches (dificultad para quedarse dormido, se despertaba a media noche, se despertaba temprano en la mañana o dormía excesivamente)?	NO	SÍ	4
	c	¿Casi todos los días, hablaba o se movía usted más lento de lo usual, o estaba inquieto o tenía dificultades para permanecer tranquilo?	NO	SÍ	5
	d	¿Casi todos los días, se sentía la mayor parte del tiempo fatigado o sin energía?	NO	SÍ	6
	e	¿Casi todos los días, se sentía culpable o inútil?	NO	SÍ	7
	f	¿Casi todos los días, tenía dificultad para concentrarse o tomar decisiones?	NO	SÍ	8
	g	¿En varias ocasiones, deseó hacerse daño, se sintió suicida, o deseó estar muerto?	NO	SÍ	9

¿CODIFICÓ **SÍ** EN **5** O MÁS RESPUESTAS (**A1-A3**)?

**NO**	**SÍ**
EPISODIO DEPRESIVO MAYOR ACTUAL	

SI EL PACIENTE CODIFICA POSITIVO PARA UN EPISODIO DEPRESIVO MAYOR ACTUAL, CONTINÚE CON A4, DE LO CONTRARIO CONTINÚE CON EL MÓDULO B:

A4	a	¿En el transcurso de su vida, tuvo otros períodos de dos o más semanas, en los que se sintió deprimido o sin interés en la mayoría de las cosas y que tuvo la mayoría de los problemas de los que acabamos de hablar?	➡ NO	SÍ	10
	b	¿Ha tenido alguna vez un período de por lo menos dos meses, sin depresión o sin la falta de interés en la mayoría de las cosas y ocurrió este período entre dos episodios depresivos?	**NO**	**SÍ**	11

**NO**	**SÍ**	11
EPISODIO DEPRESIVO MAYOR RECIDIVANTE		

MINI 5.0.0 (1 de enero de 2000)

Episodio depresivo mayor con síntomas melancólicos (opcional)

(➡ SIGNIFICA: IR A LAS CASILLAS DIAGNÓSTICAS, RODEAR CON UN CÍRCULO NO Y CONTINUAR CON EL SIGUIENTE MÓDULO)

SI EL PACIENTE CODIFICA POSITIVO PARA UN EPISODIO DEPRESIVO MAYOR ACTUAL (**A3** = **SÍ**), EXPLORAR LO SIGUIENTE:

A5	a	¿CODIFICÓ **SÍ** EN **A2**?	NO	SÍ	
	b	¿Durante el período más grave del episodio depresivo actual, perdió la capacidad de reaccionar a las cosas que previamente le daban placer o le animaban?<br><br>**Si NO:** ¿Cuando algo bueno le sucede, no logra hacerle sentirse mejor aunque sea temporalmente?	NO	SÍ	12
		¿CODIFICÓ **SÍ** EN **A5a** O **A5b**?	➡ NO	SÍ	
A6		**Durante las últimas 2 semanas, cuando se sintió deprimido o sin interés en la mayoría de las cosas:**			
	a	¿Se sentía deprimido de una manera diferente al tipo de sentimiento que ha experimentado cuando alguien cercano a usted se ha muerto?	NO	SÍ	13
	b	¿Casi todos los días, por lo regular se sentía peor en las mañanas?	NO	SÍ	14
	c	¿Casi todos los días, se despertaba por lo menos dos horas antes de su hora habitual, y tenía dificultades para volver a dormirse?	NO	SÍ	15
	d	¿CODIFICÓ **SÍ** EN **A3c** (ENLENTECIMIENTO O AGITACIÓN PSICOMOTORA)?	NO	SÍ	
	e	¿CODIFICÓ **SÍ** EN **A3a** (ANOREXIA O PÉRDIDA DE PESO)?	NO	SÍ	
	f	¿Se sentía excesivamente culpable o era su sentimiento de culpa desproporcionado con la realidad de la situación?	NO	SÍ	16

¿CODIFICÓ **SÍ** EN **3** O MÁS RESPUESTAS DE **A6**?

**NO**	**SÍ**
EPISODIO DEPRESIVO MAYOR CON SÍNTOMAS MELANCÓLICOS ACTUAL	

MINI 5.0.0 (1 de enero de 2000)

B. Trastorno distímico

(➡ SIGNIFICA: IR A LAS CASILLAS DIAGNÓSTICAS, RODEAR CON UN CÍRCULO NO Y CONTINUAR CON EL SIGUIENTE MÓDULO)

SI LOS SÍNTOMAS DEL PACIENTE ACTUALMENTE CUMPLEN CON LOS CRITERIOS DE UN EPISODIO DEPRESIVO MAYOR, NO EXPLORE ESTE MÓDULO

B1		¿En los últimos 2 años, se ha sentido triste, desanimado o deprimido la mayor parte del tiempo?	➡ NO	SÍ	17
B2		¿Durante este tiempo, ha habido algún período de 2 meses o más, en el que se haya sentido bien?	NO	➡ SÍ	18
B3		**Durante este período en el que se sintió deprimido la mayor parte del tiempo:**			
	a	¿Cambió su apetito notablemente?	NO	SÍ	19
	b	¿Tuvo dificultad para dormir o durmió en exceso?	NO	SÍ	20
	c	¿Se sintió cansado o sin energía?	NO	SÍ	21
	d	¿Perdió la confianza en sí mismo?	NO	SÍ	22
	e	¿Tuvo dificultades para concentrarse o para tomar decisiones?	NO	SÍ	23
	f	¿Tuvo sentimientos de desesperanza?	NO	SÍ	24
		¿CODIFICÓ **SÍ** EN **2** O MÁS RESPUESTAS DE **B3**?	➡ NO	SÍ	
B4		¿Estos síntomas de depresión, le causaron gran angustia o han interferido con su función en el trabajo, socialmente o de otra manera importante?	➡ NO	SÍ	25

¿CODIFICÓ **SÍ** EN **B4**?

**NO**	**SÍ**
TRASTORNO DISTÍMICO ACTUAL	

MINI 5.0.0 (1 de enero de 2000)

C. Riesgo de suicidio

	**Durante este último mes:**			**Puntos:**
C1	¿Ha pensado que estaría mejor muerto, o ha deseado estar muerto?	NO	SÍ	1
C2	¿Ha querido hacerse daño?	NO	SÍ	2
C3	¿Ha pensado en el suicidio?	NO	SÍ	6
C4	¿Ha planeado cómo suicidarse?	NO	SÍ	10
C5	¿Ha intentado suicidarse?	NO	SÍ	10
	**A lo largo de su vida:**			
C6	¿Alguna vez ha intentado suicidarse?	NO	SÍ	4

¿CODIFICÓ **SÍ** EN POR LO MENOS **1** RESPUESTA?

NO **SÍ**

RIESGO DE SUICIDIO

1-5 puntos	Leve	☐
6-9 puntos	Moderado	☐
≥ 10 puntos	Alto	☐

SI **SÍ**, SUME EL NÚMERO TOTAL DE PUNTOS DE LAS RESPUESTAS (C1-C6) RODEAR CON UN CÍRCULO «SÍ» Y ESPECIFICAR EL NIVEL DE RIESGO DE SUICIDIO

MINI 5.0.0 (1 de enero de 2000)

D. Episodio (hipo)maníaco

(➡ SIGNIFICA: IR A LAS CASILLAS DIAGNÓSTICAS, RODEAR CON UN CÍRCULO NO EN CADA UNA Y CONTINUAR CON EL SIGUIENTE MÓDULO)

D1	a	¿Alguna vez ha tenido un período de tiempo en el que se ha sentido exaltado, eufórico, o tan lleno de energía, o seguro de sí mismo, que esto le ha ocasionado problemas u otras personas han pensado que usted no estaba en su estado habitual? (No considere períodos en el que estaba intoxicado con drogas o alcohol.)	NO	SÍ	1
		SI EL PACIENTE PARECE CONFUNDIDO O NO ENTIENDE A LO QUE SE REFIERE CON «EXALTADO» O «EUFÓRICO», CLARIFÍQUESELO DE LA SIGUIENTE MANERA: Lo que queremos decir con «exaltado o «eufórico» es un estado de satisfacción alto, lleno de energía, en el que se necesita dormir menos, en el que los pensamientos se aceleran, en el que se tienen muchas ideas, en el que aumenta la productividad, la creatividad, la motivación o el comportamiento impulsivo.			
		SI **SÍ**:			
	b	¿En este momento se siente «exaltado», «eufórico», o lleno de energía?	NO	SÍ	2
D2	a	¿Ha estado usted alguna vez persistentemente irritado durante varios días, de tal manera que tenía discusiones, peleaba o le gritaba a personas fuera de su familia? ¿Ha notado usted o los demás, que ha estado más irritable o que reacciona de una manera exagerada, comparado a otras personas, en situaciones que incluso usted creía justificadas?	NO	SÍ	3
		SI **SÍ:**			
	b	¿En este momento se siente excesivamente irritable?	NO	SÍ	4
		¿CODIFICÓ **SÍ** EN **D1a** O EN **D2a**?	➡ NO	SÍ	

D3		SI **D1b** O **D2b** = **SÍ:** EXPLORAR SOLAMENTE EL EPISODIO **ACTUAL**<br>SI **D1b** Y **D2b** = **NO:** EXPLORAR EL EPISODIO **PASADO** MÁS SINTOMÁTICO			
		**Durante el tiempo en el que se sentía exaltado, lleno de energía, o irritable notó que:**			
	a	¿Sentía que podía hacer cosas que otros no podían hacer, o que usted era una persona especialmente importante?	NO	SÍ	5
	b	¿Necesitaba dormir menos (p. ej., se sentía descansado con pocas horas de sueño)?	NO	SÍ	6
	c	¿Hablaba usted sin parar o tan deprisa que los demás tenían dificultad para entenderle?	NO	SÍ	7
	d	¿Sus pensamientos pasaban tan deprisa por su cabeza que tenía dificultades para seguirlos?	NO	SÍ	8
	e	¿Se distraía tan fácilmente, que la menor interrupción le hacía perder el hilo de lo que estaba haciendo o pensando?	NO	SÍ	9
	f	¿Estaba tan activo, tan inquieto físicamente que los demás se preocupaban por usted?	NO	SÍ	10
	g	¿Quería involucrarse en actividades tan placenteras, que ignoró los riesgos o consecuencias (p. ej., se embarcó en gastos descontrolados, condujo imprudentemente o mantuvo actividades sexuales indiscretas)?	NO	SÍ	11
		¿CODIFICÓ **SÍ** EN **3** O MÁS RESPUESTAS DE **D3**<br>(O **4** O MÁS RESPUESTAS SI **D1a** ES **NO** [EPISODIO PASADO]<br>O SI **D1b** ES **NO** [EPISODIO ACTUAL])?	➡ NO	SÍ	

MINI 5.0.0 (1 de enero de 2000)

		NO	SÍ	
D4	¿Duraron estos síntomas al menos 1 semana y le causaron problemas que estaban fuera de su control, en la casa, en el trabajo, en la escuela, o fue usted hospitalizado a causa de estos problemas?	↓	↓	12
	EL EPISODIO EXPLORADO ERA:	☐ *HIPOMANÍACO*	☐ *MANÍACO*	

¿CODIFICÓ **NO** EN **D4**?

ESPECIFICAR SI EL EPISODIO ES ACTUAL O PASADO.

**NO**	**SÍ**
EPISODIO HIPOMANÍACO	
ACTUAL	☐
PASADO	☐

¿CODIFICÓ **SÍ** EN **D4**?

ESPECIFICAR SI EL EPISODIO ES ACTUAL O PASADO.

**NO**	**SÍ**
EPISODIO MANÍACO	
ACTUAL	☐
PASADO	☐

MINI 5.0.0 (1 de enero de 2000)

E. Trastorno de angustia

(➡ SIGNIFICA: RODEAR CON UN CÍRCULO NO EN E5 Y PASAR DIRECTAMENTE A F1)

E1	a	¿En más de una ocasión, tuvo una crisis o ataques en los cuales se sintió **súbitamente** ansioso, asustado, incómodo o inquieto, incluso en situaciones en la cual la mayoría de las personas no se sentirían así?	➡ NO	SÍ	1
	b	¿Estas crisis o ataques alcanzan su máxima expresión en los primeros 10 minutos?	➡ NO	SÍ	2
E2		¿Alguna vez estas crisis o ataques o ocurrieron de una manera inesperada o espontánea u ocurrieron de forma impredecible o sin provocación?	NO	SÍ	3
E3		¿Ha tenido una de estas crisis seguida por un período de un mes o más en el que temía que otro episodio recurriera o se preocupaba por las consecuencias de la crisis?	NO	SÍ	4
E4		**Durante la peor crisis que usted puede recordar:**			
	a	¿Sentía que su corazón le daba un vuelco, latía más fuerte o más rápido?	NO	SÍ	5
	b	¿Sudaba o tenía las manos húmedas?	NO	SÍ	6
	c	¿Tenía temblores o sacudidas musculares?	NO	SÍ	7
	d	¿Sentía la falta de aliento o dificultad para respirar?	NO	SÍ	8
	e	¿Tenía sensación de ahogo o un nudo en la garganta?	NO	SÍ	9
	f	¿Notaba dolor o molestia en el pecho?	NO	SÍ	10
	g	¿Tenía náuseas, molestias en el estómago o diarreas repentinas?	NO	SÍ	11
	h	¿Se sentía mareado, inestable, aturdido o a punto de desvanecerse?	NO	SÍ	12
	i	¿Le parecía que las cosas a su alrededor eran irreales, extrañas, indiferentes, o no le parecían familiares, o se sintió fuera o separado de su cuerpo o de partes de su cuerpo?	NO	SÍ	13
	j	¿Tenía miedo de perder el control o de volverse loco?	NO	SÍ	14
	k	¿Tenía miedo de que se estuviera muriendo?	NO	SÍ	15
	l	¿Tenía alguna parte de su cuerpo adormecida o con hormigueos?	NO	SÍ	16
	m	¿Tenía sofocaciones o escalofríos?	NO	SÍ	17
E5		¿CODIFICÓ **SÍ** EN **E3** Y EN POR LO MENOS **4** DE **E4**?	NO	SÍ<br>***Trastorno de angustia de por vida***	
E6		SI **E5** = **NO**, ¿CODIFICÓ **SÍ** EN ALGUNA RESPUESTA DE **E4**?<br>SI **E6** = **SÍ**, PASAR A **F1**.	NO	SÍ<br>***Crisis actual con síntomas limitados***	
E7		¿En el pasado mes, tuvo estas crisis en varias ocasiones (2 o más), seguidas de miedo persistente a tener otra?	NO	SÍ<br>***Trastorno de angustia actual***	18

MINI 5.0.0 (1 de enero de 2000)

F. Agorafobia

F1	¿Se ha sentido particularmente incómodo o ansioso en lugares o situaciones donde podría tener una crisis o ataque, o síntomas de una crisis como los que acabamos de discutir, o situaciones donde no dispondría de ayuda o escapar pudiera resultar un tanto difícil: como estar en una multitud, permanecer en fila, estar solo fuera de casa, permanecer solo en casa, viajar en autobús, tren o automóvil?	NO	SÍ	19
	SI **F1** = **NO**, RODEE CON UN CÍRCULO **NO** en **F2**.			
F2	¿Teme tanto estas situaciones que las evita, sufre en ellas o necesita estar acompañado para enfrentarlas?	NO	SÍ<br>***Agorafobia actual***	20

¿CODIFICÓ **NO** EN **F2** (AGORAFOBIA ACTUAL)

Y

CODIFICÓ **SÍ** EN **E7** (TRASTORNO DE ANGUSTIA ACTUAL)?

NO **SÍ**
***TRASTORNO DE ANGUSTIA sin agorafobia* ACTUAL**

¿CODIFICÓ **SÍ** EN **F2** (AGORAFOBIA ACTUAL)

Y

CODIFICÓ **SÍ** EN **E7** (TRASTORNO DE ANGUSTIA ACTUAL)?

NO **SÍ**
***TRASTORNO DE ANGUSTIA con agorafobia* ACTUAL**

¿CODIFICÓ **SÍ** EN **F2** (AGORAFOBIA ACTUAL)

Y

CODIFICÓ **NO** EN **E5** (TRASTORNO DE ANGUSTIA DE POR VIDA)?

NO **SÍ**
AGORAFOBIA* ACTUAL *sin historial de trastorno de angustia

MINI 5.0.0 (1 de enero de 2000)

G. Fobia social (trastorno de ansiedad social)

(➡ SIGNIFICA: IR A LAS CASILLAS DIAGNÓSTICAS, RODEAR CON UN CÍRCULO NO EN CADA UNA Y CONTINUAR CON EL SIGUIENTE MÓDULO)

G1	¿En el pasado mes, tuvo miedo o sintió vergüenza de que lo estén observando, de ser el centro de atención o temió una humillación? Incluyendo cosas como el hablar en público, comer en público o con otros, el escribir mientras alguien le mira o el estar en situaciones sociales.	➡ NO	SÍ	1
G2	¿Piensa usted que este miedo es excesivo o irracional?	➡ NO	SÍ	2
G3	¿Teme tanto estas situaciones sociales que las evita, o sufre en ellas?	➡ NO	SÍ	3
G4	¿Este miedo interfiere en su trabajo normal o en el desempeño de sus actividades sociales o es la causa de intensa molestia?	**NO**	**SÍ**	**4**
		FOBIA SOCIAL (trastorno de ansiedad social) **ACTUAL**		

MINI 5.0.0 (1 de enero de 2000)

H. Trastorno obsesivo-compulsivo

(➡ SIGNIFICA: IR A LAS CASILLAS DIAGNÓSTICAS, MARCAR CON UN CÍRCULO NO Y CONTINUAR CON EL SIGUIENTE MÓDULO)

H1	¿Este último mes, ha estado usted molesto con pensamientos recurrentes, impulsos o imágenes no deseadas, desagradables, inapropiadas, intrusas o angustiosas? (p. ej., la idea de estar sucio, contaminado o tener gérmenes, **o** miedo de contaminar a otros, **o** temor de hacerle daño a alguien sin querer, **o** temor que actuaría en función de algún impulso, **o** tiene temores o supersticiones de ser el responsable de que las cosas vayan mal, **o** se obsesiona con pensamientos, imágenes o impulsos sexuales; **o** acumula o colecciona sin control, **o** tiene obsesiones religiosas)<br><br>(NO INCLUIR PREOCUPACIONES EXCESIVAS POR PROBLEMAS DE LA VIDA COTIDIANA. NO INCLUIR OBSESIONES DIRECTAMENTE RELACIONADAS CON TRASTORNOS DE LA ALIMENTACIÓN, CONDUCTAS SEXUALES, PROBLEMAS PATOLÓGICOS RELACIONADOS CON EL JUEGO, ALCOHOL O ABUSO DE DROGAS, PORQUE EL PACIENTE PUDIERA DERIVAR PLACER DE LA ACTIVIDAD Y PUDIERA QUERER EVITARLA SIMPLEMENTE POR LAS CONSECUENCIAS NEGATIVAS)	NO<br>➡ H4	SÍ	1
H2	¿Estos pensamientos volvían a su mente aun cuando trataba de ignorarlos o de librarse de ellos?	NO<br>➡ H4	SÍ	2
H3	¿Cree usted que estos pensamientos son producto de su propia mente y que no le son impuestos desde el exterior?	NO	SÍ<br>obsesiones	3
H4	¿En el pasado mes, ha hecho usted algo repetidamente, sin ser capaz de evitarlo, como lavar o limpiar en exceso, contar y verificar las cosas una y otra vez o repetir, coleccionar, ordenar las cosas o realizar otros rituales supersticiosos?	NO	SÍ<br>compulsiones	4
	¿CODIFICÓ **SÍ** EN **H3** O EN **H4**?	➡<br>NO	SÍ	
H5	¿Reconoce usted que estas ideas obsesivas o actos compulsivos son irracionales, absurdos o excesivos?	➡<br>NO	SÍ	5
H6	¿Estas obsesiones o actos compulsivos interfieren de manera significativa con sus actividades cotidianas, con su trabajo, con sus relaciones sociales, o le ocupan más de una hora diaria?	**NO**	**SÍ**<br>***TRASTORNO OBSESIVO/ COMPULSIVO*** **ACTUAL**	**6**

MINI 5.0.0 (1 de enero de 2000)

I. Estado por estrés postraumático (opcional)

(➡ SIGNIFICA: IR A LAS CASILLAS DIAGNÓSTICAS, RODEAR CON UN CÍRCULO NO Y CONTINUAR CON EL SIGUIENTE MÓDULO)

I1		¿Ha vivido o ha sido testigo de un acontecimiento extremadamente traumático, en el cual otras personas han muerto y/u otras personas o usted mismo han estado amenazadas de muerte o en su integridad física?<br>EJEMPLOS DE ACONTECIMIENTOS TRAUMÁTICOS: ACCIDENTES GRAVES, ATRACO, VIOLACIÓN, ATENTADO TERRORISTA, SER TOMADO DE REHÉN, SECUESTRO, INCENDIO, DESCUBRIR UN CADÁVER, MUERTE SÚBITA DE ALGUIEN CERCANO A USTED, GUERRA O CATÁSTROFE NATURAL	➡<br>NO	SÍ	1
I2		¿Durante el pasado mes, ha revivido el evento de una manera angustiosa (p. ej., lo ha soñado, ha tenido imágenes vívidas, ha reaccionado físicamente o ha tenido memorias intensas)?	➡<br>NO	SÍ	2
I3		**En el último mes:**			
	a	¿Ha evitado usted pensar en este acontecimiento, o en todo aquello que se lo pudiese recordar?	NO	SÍ	3
	b	¿Ha tenido dificultad recordando alguna parte del evento?	NO	SÍ	4
	c	¿Ha disminuido su interés en las cosas que le agradaban o en las actividades sociales?	NO	SÍ	5
	d	¿Se ha sentido usted alejado o distante de otros?	NO	SÍ	6
	e	¿Ha notado que sus sentimientos están adormecidos?	NO	SÍ	7
	f	¿Ha tenido la impresión de que su vida se va a acortar debido a este trauma o que va a morir antes que otras personas?	NO	SÍ	8
		¿CODIFICÓ **SÍ** EN **3** O MÁS RESPUESTAS DE **I3**?	➡<br>NO	SÍ	
I4		**Durante el último mes:**			
	a	¿Ha tenido usted dificultades para dormir?	NO	SÍ	9
	b	¿Ha estado particularmente irritable o le daban arranques de coraje?	NO	SÍ	10
	c	¿Ha tenido dificultad para concentrarse?	NO	SÍ	11
	d	¿Ha estado nervioso o constantemente en alerta?	NO	SÍ	12
	e	¿Se ha sobresaltado fácilmente por cualquier cosa?	NO	SÍ	13
		¿CODIFICÓ **SÍ** EN **2** O MÁS RESPUESTAS DE **I3**?	➡<br>NO	SÍ	
I5		¿En el transcurso de este mes, han interferido estos problemas en su trabajo, en sus actividades sociales o han sido causa de gran ansiedad?	**NO**	**SÍ**	**14**
			ESTADO POR ESTRÉS POSTRAUMÁTICO **ACTUAL**		

MINI 5.0.0 (1 de enero de 2000)

J. Abuso y dependencia de alcohol

(➡ SIGNIFICA: IR A LAS CASILLAS DIAGNÓSTICAS, RODEAR CON UN CÍRCULO NO EN CADA UNA Y CONTINUAR CON EL SIGUIENTE MÓDULO)

J1	¿En los últimos 12 meses, ha tomado 3 o más bebidas alcohólicas en un período de 3 horas en tres o más ocasiones?	➡ NO	SÍ	1

J2 En los últimos 12 meses:

a	¿Necesitaba beber más para conseguir los mismos efectos que cuando usted comenzó a beber?	NO	SÍ	2
b	¿Cuando reducía la cantidad de alcohol, temblaban sus manos, sudaba, o se sentía agitado? ¿Bebía para evitar estos síntomas o para evitar la resaca (p. ej., temblores, sudoraciones o agitación)? CODIFICAR **SÍ**, SI CONTESTÓ **SÍ** EN ALGUNA.	NO	SÍ	3
c	¿Durante el tiempo en el que bebía alcohol, acababa bebiendo más de lo que en un principio había planeado?	NO	SÍ	4
d	¿Ha tratado de reducir o dejar de beber alcohol pero ha fracasado?	NO	SÍ	5
e	¿Los días en los que bebía, empleaba mucho tiempo en procurarse alcohol, en beber y en recuperarse de sus efectos?	NO	SÍ	6
f	¿Pasó menos tiempo trabajando, disfrutando de sus pasatiempos, o estando con otros, debido a su consumo de alcohol?	NO	SÍ	7
g	¿Continuó bebiendo a pesar de saber que esto le causaba problemas de salud, físicos o mentales?	NO	SÍ	8

¿CODIFICÓ **SÍ** EN **3** O MÁS RESPUESTAS DE **J2**?

**NO**	➡ **SÍ**
DEPENDENCIA DE ALCOHOL **ACTUAL**	

J3 En los últimos 12 meses:

a	¿Ha estado usted varias veces intoxicado, embriagado, o con resaca en más de una ocasión, cuando tenía otras responsabilidades en la escuela, el trabajo o la casa? ¿Esto le ocasionó algún problema? CODIFIQUE **SÍ** SÓLO SI ESTO LE HA OCASIONADO PROBLEMAS.	NO	SÍ	9
b	¿Ha estado intoxicado en alguna situación en la que corría un riesgo físico, por ejemplo conducir un automóvil, una motocicleta, una embarcación, utilizar una máquina, etc.)?	NO	SÍ	10
c	¿Ha tenido problemas legales debido a su uso de alcohol, por ejemplo un arresto, perturbación del orden público?	NO	SÍ	11
d	¿Ha continuado usted bebiendo a pesar de saber que esto le ocasionaba problemas con su familia u otras personas?	NO	SÍ	12

¿CODIFICÓ **SÍ** EN **1** O MÁS RESPUESTAS DE **J3**?

**NO**	**SÍ**
ABUSO DE ALCOHOL **ACTUAL**	

MINI 5.0.0 (1 de enero de 2000)

K. Trastornos asociados al uso de sustancias psicoactivas no alcohólicas

(➡ SIGNIFICA: IR A LAS CASILLAS DIAGNÓSTICAS, RODEAR CON UN CÍRCULO NO EN CADA UNA Y CONTINUAR CON EL SIGUIENTE MÓDULO)

Ahora le voy a enseñar/leer una lista de sustancias ilícitas o medicinas.

			➡		
K1	a	**¿En los últimos 12 meses,** tomó alguna de estas sustancias, en más de una ocasión, para sentirse mejor o para cambiar su estado de ánimo?	NO	SÍ	

RODEE CON UN CÍRCULO TODAS LAS SUSTANCIAS QUE HAYA USADO:

Estimulantes: anfetaminas, *speed*, cristal, dexedrina, ritalina, píldoras adelgazantes.
Cocaína: inhalada, intravenosa, crack, *speedball.*
Narcóticos: heroína, morfina, Dilaudid, opio, Demerol, metadona, codeína, Percodan, Darvon.
Alucinógenos: LSD (ácido), mescalina, peyote, PCP (polvo de ángel, *peace pill*), *psilocybin*, STP, hongos, éxtasis, MDA, MDMA.
Inhalantes: pegamento, éter, óxido nitroso (*laughing gas*), *amyl* o *butyl nitrate (poppers).*
Marihuana: hachís, THC, pasto, hierba, mota, *reefer.*
Tranquilizantes: Qualude, Seconal («reds»), Valium, Xanax, Librium, Ativan, Dalmane, Halción, barbitúricos, «Miltown», Tranquimazin, Lexatin, Orfidal.
Otras sustancias: esteroides, pastillas dietéticas o para dormir sin receta. ¿Cualquier otra sustancia?
ESPECIFIQUE LA/S SUSTANCIA/S MÁS USADA/S: ______________________

b. SI EXISTE USO CONCURRENTE O SUCESIVO DE VARIAS SUSTANCIAS O DROGAS, ESPECIFIQUE QUÉ DROGA/CLASE DE DROGA VA A SER EXPLORADA EN LA ENTREVISTA A CONTINUACIÓN:

SÓLO UNA DROGA/CLASE DE DROGA HA SIDO UTILIZADA. ☐

SÓLO LA CLASE DE DROGA MÁS UTILIZADA ES EXPLORADA. ☐

CADA DROGA ES EXAMINADA INDIVIDUALMENTE. (FOTOCOPIAR K2 Y K3 SEGÚN SEA NECESARIO.) ☐

K2 **Considerando su uso de** (NOMBRE DE LA DROGA/CLASE DE DROGAS SELECCIONADA)**, en los últimos 12 meses:**

a	¿Ha notado que usted necesitaba utilizar una mayor cantidad de (NOMBRE DE LA DROGA/CLASE DE DROGA SELECCIONADA) para obtener los mismos efectos que cuando comenzó a usarla?	NO	SÍ	1
b	¿Cuándo redujo la cantidad o dejó de utilizar (NOMBRE DE LA DROGA/CLASE DE DROGA SELECCIONADA) tuvo síntomas de abstinencia? (dolores, temblores, fiebre, debilidad, diarreas, náuseas, sudaciones, palpitaciones, dificultad para dormir, o se sentía agitado, ansioso, irritable o deprimido)? Utilizó alguna/s droga/s para evitar enfermarse (síntomas de abstinencia) o para sentirse mejor?<br><br>CODIFICAR **SÍ**, SI CONTESTÓ **SÍ** EN ALGUNA	NO	SÍ	2
c	¿Ha notado que cuando usted usaba (NOMBRE DE LA DROGA/CLASE DE DROGA SELECCIONADA) terminaba utilizando más de lo que en un principio había planeado?	NO	SÍ	3
d	¿Ha tratado de reducir o dejar de tomar (NOMBRE DE LA DROGA/CLASE DE DROGA SELECCIONADA) pero ha fracasado?	NO	SÍ	4

MINI 5.0.0 (1 de enero de 2000)

	e	¿Los días que utilizaba (NOMBRE DE LA DROGA/CLASE DE DROGA SELECCIONADA) empleaba mucho tiempo (> 2 horas) en obtener, consumir, recuperarse de sus efectos, o pensando en drogas?	NO	SÍ	5
	f	¿Pasó menos tiempo trabajando, disfrutando de pasatiempos, estando con la familia o amigos debido a su uso de drogas?	NO	SÍ	6
	g	¿Ha continuado usando (NOMBRE DE LA DROGA/CLASE DE DROGA SELECCIONADA) a pesar de saber que esto le causaba problemas mentales o de salud?	NO	SÍ	7

¿CODIFICÓ **SÍ** EN **3** O MÁS RESPUESTAS DE **K2**?
ESPECIFICAR LA/S DROGA/S: ______________________

NO ➡ **SÍ**

DEPENDENCIA DE SUSTANCIAS **ACTUAL**

Considerando su uso de (NOMBRE DE LA CLASE DE DROGA SELECCIONADA)**, en los últimos 12 meses:**

K3	a	¿Ha estado intoxicado o con resaca a causa de (NOMBRE DE LA DROGA/CLASE DE DROGA SELECCIONADA), en más de una ocasión, cuando tenía otras responsabilidades en la escuela, en el trabajo o en el hogar? ¿Esto le ocasionó algún problema? (CODIFIQUE **SÍ**, SÓLO SI LE OCASIONÓ PROBLEMAS)	NO	SÍ	8
	b	¿Ha estado intoxicado con (NOMBRE DE LA DROGA/CLASE DE DROGA SELECCIONADA) en alguna situación en la que corriese un riesgo físico (p. ej., conducir un automóvil, una motocicleta, una embarcación, o utilizar una máquina, etc.)?	NO	SÍ	9
	c	¿Ha tenido algún problema legal debido a su uso de drogas, por ejemplo, un arresto o perturbación del orden público?	NO	SÍ	10
	d	¿Ha continuado usando (NOMBRE DE LA DROGA/CLASE DE DROGA SELECCIONADA) a pesar de saber que esto le causaba problemas con su familia u otras personas?	NO	SÍ	11

¿CODIFICÓ **SÍ** EN **1** O MÁS RESPUESTAS DE **K3**?
ESPECIFICAR LA/S DROGA/S: ______________________

NO **SÍ**

ABUSO DE SUSTANCIAS **ACTUAL**

MINI 5.0.0 (1 de enero de 2000)

L. Trastornos psicóticos

(➡ SIGNIFICA: IR A LAS CASILLAS DIAGNÓSTICAS, RODEAR CON UN CÍRCULO NO EN CADA UNA Y CONTINUAR CON EL SIGUIENTE MÓDULO)

PIDA UN EJEMPLO PARA CADA PREGUNTA CONTESTADA AFIRMATIVAMENTE. CODIFIQUE **SÍ** SOLAMENTE PARA AQUELLOS EJEMPLOS QUE MUESTRAN CLARAMENTE UNA DISTORSIÓN DEL PENSAMIENTO O DE LA PERCEPCIÓN O SI NO SON CULTURALMENTE APROPIADOS. ANTES DE CODIFICAR, INVESTIGUE SI LAS IDEAS DELIRANTES CALIFICAN COMO «EXTRAÑAS» O RARAS.

LAS IDEAS DELIRANTES SON «EXTRAÑAS» O RARAS SI: SON CLARAMENTE ABSURDAS, IMPROBABLES, INCOMPRENSIBLES, Y NO PUEDEN DERIVARSE DE EXPERIENCIAS DE LA VIDA COTIDIANA.

LAS ALUCINACIONES SON «EXTRAÑAS» O RARAS SI: UNA VOZ HACE COMENTARIOS SOBRE LOS PENSAMIENTOS O LOS ACTOS DE LA PERSONA, O DOS O MÁS VOCES CONVERSAN ENTRE SÍ.

		Ahora le voy a preguntar acerca de experiencias poco usuales que algunas personas pueden tener.			EXTRAÑOS	
L1	a	¿Alguna vez ha tenido la impresión de que alguien le espiaba, o conspiraba contra usted, o que trataban de hacerle daño?<br>**NOTA:** PIDA EJEMPLOS PARA DESCARTAR UN VERDADERO ACECHO.	NO	SÍ	SÍ	1
	b	**Si SÍ:** ¿Actualmente cree usted esto?	NO	SÍ	SÍ<br>➡ L6	2
L2	a	¿Ha tenido usted la impresión de que alguien podía leer o escuchar sus pensamientos, o que usted podía leer o escuchar los pensamientos de otros?	NO	SÍ	SÍ	3
	b	**Si SÍ:** ¿Actualmente cree usted esto?	NO	SÍ	SÍ<br>➡ L6	4
L3	a	¿Alguna vez ha creído que alguien o que una fuerza externa haya metido pensamientos ajenos en su mente o le hicieron actuar de una manera no usual en usted? Alguna vez ha tenido la impresión de que está poseído?<br>ENTREVISTADOR/A: PIDA EJEMPLOS Y DESCARTE CUALQUIERA QUE NO SEA PSICÓTICO.	NO	SÍ	SÍ	5
	b	**Si SÍ:** ¿Actualmente cree usted esto?	NO	SÍ	SÍ<br>➡ L6	6
L4	a	¿Alguna vez ha creído que le envían mensajes especiales a través de la radio, el televisor o el periódico, o que una persona que no conocía personalmente se interesaba particularmente por usted?	NO	SÍ	SÍ	7
	b	**Si SÍ:** ¿Actualmente cree usted esto?	NO	SÍ	SÍ<br>➡ L6	8
L5	a	¿Consideran sus familiares o amigos que algunas de sus creencias son extrañas o poco usuales?<br>ENTREVISTADOR/A: PIDA EJEMPLOS. CODIFIQUE **SÍ** SÓLO SI LOS EJEMPLOS SON **CLARAMENTE** IDEAS DELIRANTES NO EXPLORADAS EN LAS PREGUNTAS L1 A L4, POR EJEMPLO, DELIRIOS SOMÁTICOS, RELIGIOSOS O DE GRANDEZA, CELOS, CULPA, RUINA O DESTITUCIÓN, ETC.	NO	SÍ	SÍ	9
	b	**Si SÍ:** ¿Actualmente, consideran los demás sus ideas como extrañas?	NO	SÍ	SÍ	10
L6	a	¿Alguna vez ha escuchado cosas que otras personas no podían escuchar, como voces?	NO	SÍ		11
		LAS ALUCINACIONES SON CODIFICADAS COMO «EXTRAÑAS» SOLAMENTE SI EL PACIENTE CONTESTA **SÍ** A LO SIGUIENTE:				
		**Si SÍ:** ¿Escuchó una voz que comentaba acerca de sus pensamientos o sus actos, o escuchó dos o más voces conversando entre sí?			SÍ	
	b	**Si SÍ:** ¿Ha escuchado estas cosas en el pasado mes?	NO	SÍ	SÍ<br>➡ L8b	12

MINI 5.0.0 (1 de enero de 2000)

L7	a	¿Alguna vez, estando despierto, ha tenido visiones o ha visto cosas que otros no podían ver?<br>ENTREVISTADOR/A: INVESTIGUE SI ESTAS VISIONES SON CULTURALMENTE INAPROPIADAS.	NO	SÍ	13
	b	**Si SÍ:** ¿Ha visto estas cosas el pasado mes?	NO	SÍ	14
		**BAJO EL PUNTO DE VISTA DEL ENTREVISTADOR/a:**			
L8	b	¿PRESENTA EL PACIENTE ACTUALMENTE UN LENGUAJE DESORGANIZADO, INCOHERENTE O CON MARCADA PÉRDIDA DE LAS ASOCIACIONES?	NO	SÍ	15
L9	b	¿PRESENTA EL PACIENTE ACTUALMENTE UN COMPORTAMIENTO DESORGANIZADO O CATATÓNICO?	NO	SÍ	16
L10	b	¿HAY SÍNTOMAS NEGATIVOS DE ESQUIZOFRENIA PROMINENTES DURANTE LA ENTREVISTA (UN APLANAMIENTO AFECTIVO SIGNIFICATIVO, POBREZA DEL LENGUAJE [ALOGIA] O INCAPACIDAD PARA INICIAR O PERSISTIR EN ACTIVIDADES CON UNA FINALIDAD DETERMINADA)?	NO	SÍ	17

L11 ¿CODIFICÓ **SÍ EXTRAÑO** EN 1 O MÁS PREGUNTAS «b»?

o

¿CODIFICÓ **SÍ** (EN VEZ DE **SÍ EXTRAÑO**) EN 2 O MÁS PREGUNTAS «b»?

> **NO** **SÍ**
>
> ***TRASTORNO PSICÓTICO***
> **ACTUAL**

L12 ¿CODIFICÓ **SÍ EXTRAÑO** EN 1 O MÁS PREGUNTAS «a»?

o

¿CODIFICÓ **SÍ** (EN VEZ DE **SÍ EXTRAÑO**) EN 2 O MÁS PREGUNTAS «a»?

VERIFIQUE QUE LOS DOS SÍNTOMAS OCURRIERAN DURANTE EL MISMO PERÍODO DE TIEMPO

o ¿CODIFICÓ **SÍ** EN **L11**?

> **NO** **SÍ** **18**
>
> ***TRASTORNO PSICÓTICO***
> **DE POR VIDA**

L13 a ¿CODIFICÓ **SÍ** EN 1 O MÁS PREGUNTAS DE **L1b** A **L7b** Y CODIFICÓ **SÍ** EN
EPISODIO DEPRESIVO MAYOR (ACTUAL)
o
EPISODIO MANÍACO (ACTUAL O PASADO)? ➡ NO SÍ

b SI CODIFICÓ SÍ EN L1EA:

Anteriormente me dijo que usted tuvo un período/s en el que se sintió (deprimido[a]/exaltado[a]/particularmente irritable).
Estas creencias o experiencias que me acaba de describir (SÍNTOMAS CODIFICADOS SÍ DE **L1b** a **L7b**) ¿Se limitaban exclusivamente a los períodos en los que se sintió deprimido(a)/exaltado(a)/irritable?

> **NO** **SÍ** **19**
>
> **TRASTORNO**
> **DEL ESTADO DE ÁNIMO**
> *CON*
> *SÍNTOMAS PSICÓTICOS*
> **ACTUAL**

MINI 5.0.0 (1 de enero de 2000)

M. Anorexia nerviosa

(➡ SIGNIFICA: IR A LAS CASILLAS DIAGNÓSTICAS, RODEAR CON UN CÍRCULO NO EN CADA UNA Y CONTINUAR CON EL SIGUIENTE MÓDULO)

M1	a	¿Cuál es su estatura?	☐ pies ☐ ☐ pulgadas<br>☐ ☐ ☐ cm	
	b	¿En los últimos 3 meses, cuál ha sido su peso más bajo?	☐ ☐ ☐ libras<br>☐ ☐ ☐ kg	
	c	¿ES EL PESO DEL PACIENTE INFERIOR AL PESO MÍNIMO CORRESPONDIENTE A SU ESTATURA (VER TABLA A CONTINUACIÓN)?	➡ NO	SÍ

En los últimos 3 meses:

M2		¿A pesar de su bajo peso, evitaba engordar?	➡ NO	SÍ	1
M3		¿A pesar de estar bajo peso, temía ganar peso o ponerse gordo/a?	➡ NO	SÍ	2
M4	a	¿Se consideraba gordo, o que una parte de su cuerpo era demasiado gorda?	NO	SÍ	3
	b	¿Influyó mucho su peso o su figura en la opinión que usted tenía de sí mismo?	NO	SÍ	4
	c	¿Pensaba usted que su bajo peso era normal o excesivo?	NO	SÍ	5
M5		¿CODIFICÓ **SÍ** EN UNA O MÁS RESPUESTAS DE **M4**?	➡ NO	SÍ	
M6		SÓLO PARA MUJERES: ¿En los últimos 3 meses, dejó de tener todos sus períodos menstruales, aunque debió tenerlos (cuando no estaba embarazada)?	➡ NO	SÍ	6

PARA MUJERES: ¿CODIFICÓ **SÍ** EN **M5** Y **M6**?

PARA HOMBRES: ¿CODIFICÓ **SÍ** EN **M5**?

NO **SÍ**

ANOREXIA NERVIOSA **ACTUAL**

TABLA UMBRAL DE ESTATURA/PESO MÍNIMO (estatura sin zapatos; peso sin ropa)

**Mujer estatura/peso**														
Pies/pulgadas	4,9	4,10	4,11	5,0	5,1	5,2	5,3	5,4	5,5	5,6	5,7	5,8	5,9	5,10
Libras	84	85	86	87	89	92	94	97	99	102	104	107	110	112
cm	144,8	147,3	149,9	152,4	154,9	157,5	160,0	162,6	165,1	167,6	170,2	172,7	175,3	177,8
kg	38	39	39	40	41	42	43	44	45	46	47	49	50	51

**Hombre estatura/peso**														
Pies/pulgadas	5,1	5,2	5,3	5,4	5,5	5,6	5,7	5,8	5,9	5,10	5,11	6,0	6,1	6,2	6,3
Libras	105	106	108	110	111	113	115	116	118	120	122	125	127	130	133
cm	154,9	157,5	160,0	162,6	165,1	167,6	170,2	172,7	175,3	177,8	180,3	182,9	185,4	188,0	190,5
kg	47	48	49	50	51	51	52	53	54	55	56	57	58	59	61

Los umbrales de pesos anteriormente mencionados son calculados con un 15 % por debajo de la escala normal de la estatura y sexo del paciente como es requerido por el DSM-IV. Esta tabla refleja los pesos con un 15 % por debajo del límite inferior de la escala de distribución normal de la *Metropolitan Life Insurance Table of Weights.*

MINI 5.0.0 (1 de enero de 2000)

N. Bulimia nerviosa

(➡ SIGNIFICA: IR A LAS CASILLAS DIAGNÓSTICAS, RODEAR CON UN CÍRCULO NO EN CADA UNA Y CONTINUAR CON EL SIGUIENTE MÓDULO)

N1	¿En los últimos 3 meses, se ha dado usted atracones, en los cuales comía grandes cantidades de alimentos en un período de 2 horas?	➡ NO	SÍ	7
N2	¿En los últimos 3 meses, se ha dado usted al menos 2 atracones por semana?	➡ NO	SÍ	8
N3	¿Durante estos atracones, se siente descontrolado comiendo?	➡ NO	SÍ	9
N4	¿Hace usted algo para compensar o evitar ganar peso como consecuencia de estos atracones, como vomitar, ayunar, practicar ejercicio, tomar laxantes, enemas, diuréticos (pastillas de agua) u otros medicamentos?	➡ NO	SÍ	10
N5	¿Influye grandemente en la opinión que usted tiene de sí mismo su peso o la figura de su cuerpo?	➡ NO	SÍ	11
N6	¿CUMPLEN LOS SÍNTOMAS DEL PACIENTE CON LOS CRITERIOS DE ANOREXIA NERVIOSA?	NO ↓ Ir a N8	SÍ	
N7	¿Ocurren estos atracones solamente cuando está por debajo de (_____libras/kg)? (ENTREVISTADOR/A: ESCRIBA EN EL PARÉNTESIS EL PESO MÍNIMO DE ESTE PACIENTE EN RELACIÓN A SU ESTATURA, BASADO EN LA TABLA DE ESTATURA/PESO QUE SE ENCUENTRA EN EL MÓDULO DE ANOREXIA NERVIOSA.)	NO	SÍ	12

N8 ¿CODIFICÓ **SÍ** EN **N5** O CODIFICÓ **NO** EN **N7** O SALTÓ A **N8**?

NO **SÍ**
***BULIMIA NERVIOSA* ACTUAL**

¿CODIFICÓ **SÍ** EN **N7**?

NO **SÍ**
ANOREXIA NERVIOSA *TIPO COMPULSIVO/PURGATIVO* **ACTUAL**

MINI 5.0.0 (1 de enero de 2000)

O. Trastorno de ansiedad generalizada

(➡ SIGNIFICA: IR A LAS CASILLAS DIAGNÓSTICAS, RODEAR CON UN CÍRCULO NO EN CADA UNA Y CONTINUAR CON EL SIGUIENTE MÓDULO)

O1	a	¿Se ha sentido excesivamente preocupado o ansioso debido a varias cosas durante los últimos 6 meses?	➡ NO	SÍ	1
	b	¿Se presentan estas preocupaciones casi todos los días?	➡ NO	SÍ	2
		CODIFICAR SÍ, SI LA ANSIEDAD DEL PACIENTE ES RESTRINGIDA EXCLUSIVAMENTE, O MEJOR EXPLICADA POR CUALQUIERA DE LOS TRASTORNOS PREVIAMENTE DISCUTIDOS.	NO	➡ SÍ	3
O2		¿Le resulta difícil controlar estas preocupaciones o interfieren para concentrarse en lo que hace?	➡ NO	SÍ	4
O3		CODIFIQUE **NO** SI LOS SÍNTOMAS SE LIMITAN A RASGOS DE CUALQUIERA DE LOS TRASTORNOS PREVIAMENTE EXPLORADOS.			
		**En los últimos 6 meses cuando estaba ansioso, casi todo el tiempo:**			
	a	¿Se sentía inquieto, intranquilo o agitado?	NO	SÍ	5
	b	¿Se sentía tenso?	NO	SÍ	6
	c	¿Se sentía cansado, flojo o se agotaba fácilmente?	NO	SÍ	7
	d	¿Tenía dificultad para concentrarse, o notaba que la mente se le quedaba en blanco?	NO	SÍ	8
	e	¿Se sentía irritable?	NO	SÍ	9
	f	¿Tenía dificultad durmiendo (dificultad para quedarse dormido, se despertaba a media noche o demasiado temprano, o dormía en exceso)?	NO	SÍ	10

¿CODIFICÓ **SÍ** EN **3** O MÁS RESPUESTAS DE **O3**?

**NO**	**SÍ**
TRASTORNO DE ANSIEDAD GENERALIZADA **ACTUAL**	

MINI 5.0.0 (1 de enero de 2000)

P. Trastorno antisocial de la personalidad (opcional)

(➡ SIGNIFICA: IR A LAS CASILLAS DIAGNÓSTICAS Y RODEAR CON UN CÍRCULO NO)

P1		**Antes de cumplir los 15 años:**			
	a	¿Faltaba a la escuela o se escapaba y dormía fuera de casa con frecuencia?	NO	SÍ	1
	b	¿Mentía, hacía trampa, estafaba o robaba con frecuencia?	NO	SÍ	2
	c	¿Iniciaba peleas o incitaba a otros, los amenazaba o los intimidaba?	NO	SÍ	3
	d	¿Destruía cosas deliberadamente o empezaba fuegos?	NO	SÍ	4
	e	¿Maltrataba a los animales o a las personas deliberadamente?	NO	SÍ	5
	f	¿Forzó a alguien a tener relaciones sexuales con usted?	NO	SÍ	6
		¿CODIFICÓ **SÍ** EN **2** O MÁS RESPUESTAS DE **P1**?	➡ NO	SÍ	
		NO CODIFIQUE **SÍ**, SI LA CONDUCTA ES SÓLO POR MOTIVOS POLÍTICOS O RELIGIOSOS.			
P2		**Después de cumplir los 15 años:**			
	a	¿Se ha comportado repetidamente de una forma que otros considerarían irresponsable, como no pagar sus deudas, ser deliberadamente impulsivo o deliberadamente no trabajar para mantenerse?	NO	SÍ	7
	b	¿Ha hecho cosas que son ilegales incluso si no ha sido descubierto (p. ej., destruir la propiedad, robar artículos en las tiendas, hurtar, vender drogas o cometer algún tipo de delito)?	NO	SÍ	8
	c	¿Ha participado repetidamente en peleas físicas (incluyendo las peleas que tuviera con su cónyuge o con sus hijos)?	NO	SÍ	9
	d	¿Ha mentido o estafado a otros con el objetivo de conseguir dinero o por placer, o mintió para divertirse?	NO	SÍ	10
	e	¿Ha expuesto a otros a peligros sin que le importara?	NO	SÍ	11
	f	¿No ha sentido culpabilidad después de hacerle daño a otros, maltratarlos, mentirles o robarles, o después de dañar la propiedad de otros?	NO	SÍ	12

¿CODIFICÓ **SÍ** EN **3** O MÁS RESPUESTAS DE **P2**?

NO **SÍ**

TRASTORNO ANTISOCIAL DE LA PERSONALIDAD
DE POR VIDA

MINI 5.0.0 (1 de enero de 2000)

ESTO CONCLUYE LA ENTREVISTA

Referencias

Amorim P, Lecrubier Y, Weiller E, Hergueta T, Sheehan D. DSM-III-R Psychotic Disorders: procedural validity of the MINI International Neuropsychiatric Interview (MINI). Concordance and causes for discordance with the CIDI. European Psychiatry 1998; 13: 26-34.

Lecrubier Y, Sheehan D, Weiller E, Amorim P, Bonora LI, Sheehan K, Janavs J, Dunbar G. The MINI International Neuropsychiatric Interview (MINI). A Short Diagnostic Structured Interview: Reliability and Validity According to the CIDI. European Psychiatry 1997; 12: 224-231.

Sheehan DV, Lecrubier Y, Harnett-Sheehan K, Janavs J, Weiller E, Bonora LI, Keskiner A, Schinka J, Knapp E, Sheehan MF, Dunbar GC. Reliability and Validity of the MINI International Neuropsychiatric Interview (MINI): According to the SCID-P. European Psychiatry 1997; 12: 232-241.

Sheehan DV, Lecrubier Y, Harnett-Sheehan K, Amorim P, Janavs J, Weiller E, Hergueta T, Baker R, Dunbar G. The MINI International Neuropsychiatric Interview (MINI): The Development and Validation of a Structured Diagnostic Psychiatric Interview. J Clin Psychiatry 1998; 59 (Suppl 20): 22-23.

MINI 5.0.0 (1 de enero de 2000)

Traducciones	*MINI 4.4 o versiones previas*	*MINI 4.6/5.0, MINI Plus 4.6/5.0 y MINI Screen 5.0*
Africano		R. Emsley
Alemán	I. v. Denffer, M. Ackenheil, R. Dietz-Bauer	G. Stotz, R. Dietz-Bauer, M. Ackenheil
Árabe		O. Osman, E. Al-Radi
Bengalí		H. Banerjee, A. Banerjee
Búlgaro		L.G. Hranov
Checo		P. Zvlosky
Chino		L. Carroll, K-d Juang
Coreano		En preparación
Croata		En preparación
Danés	P. Bech	P. Bech, T. Schütze
Esloveno	M. Kocmur	M. Kocmur
Español	L. Ferrando, J. Bobes-García, J. Gilbert-Rahola, Y. Lecrubier	L. Ferrando, L. Franco-Alfonso, M. Soto, J. Bobes-García, O. Soto, L. Franco, G. Heinze
Estonio		J. Shlik, A. Aluoja, E. Khil
Farsi/Persa		K. Khooshabi, A. Zomorodi
Finés	M. Heikkinen, M. Lijeström, O. Tuominen	M. Heikkinen, M. Lijeström, O. Tuominen
Francés	Y. Lecrubier, E. Weiller, L. Bonora, P. Amorim, J.P. Lepine	Y. Lecrubier, E. Weiller, P. Amorim, T. Hergueta
Griego	S. Beratis	T. Calligas, S. Beratis
Gujarati		M. Patel, B. Patel
Hebreo	J. Zohar, Y. Sasson	R. Barda, I. Levinson
Hindi		C. Mittal, K. Batra, S. Gambhir
Holandés/Flamenco	E. Griez, K. Shruers, T. Overbeek, K. Demyttenaere	I. Van Vliet, H. Leroy, H. van Megen
Húngaro	I. Bitter, J. Balazs	I. Bitter, J. Balazs
Inglés	D. Sheehan, J. Janavs, R. Baker, K. Harnett-Sheehan, E. Knapp, M. Sheehan	D. Sheehan, R. Baker, J. Janavs, K. Harnett-Sheehan, M. Sheehan
Islandés		J.G. Stefansson
Italiano	L. Bonora, L. Conti, M. Piccinelli, M. Tansella, G. Cassano, Y. Lecrubier, P. Donda, E. Weiller	L. Conti, A. Rossi, P. Donda
Japonés		T. Otsubo, H. Watanabe, H. Miyaoka, K. Kamijima, J. Shinoda, K. Tanaka, Y. Okajima
Letón	V. Janavs, J. Janavs, I. Nagobads	V. Janavs, J. Janavs
Noruego	G. Pedersen, S. Blomhoff	K.A. Leiknes, U. Malt, E. Malt, S. Leganger
Polaco	M. Masiak, E. Jasiak	M. Masiak, E. Jasiak
Portugués	P. Amorim, T. Guterres	
Portugués-brasileño	P. Amorim	P. Amorim
Punjabi		A. Gahunia, S. Gambhir
Rumano		O. Driga
Ruso		A. Bystritsky, E. Selivra, M. Bystritsky
Serbio	I. Timotijevic	I. Timotijevic
Setswana		K. Ketlogetswe
Sueco	M. Waern, S. Andersch, M. Humble	C. Allgulander, M. Waern, A. Brimse, M. Humble, H. Agren
Turco	T. Örnek, A. Keskiner, I. Vahip	T. Örnek, A. Keskiner
Urdú		A. Taj, S. Gambhir

Un estudio de validez de este instrumento fue posible, en parte, por una beca del SmithKline Beecham y la European Commission. Los autores dan su agradecimiento a la Dra. Pauline Powers por sus recomendaciones en las secciones sobre anorexia nerviosa y bulimia.

MINI 5.0.0 (1 de enero de 2000)

**TEST CORTO DE ORIENTACIÓN, MEMORIA Y CONCENTRACÓN DE 6 ITEMS (KATZMAN)**	Nombre del paciente:<br>Nombre del evaluador:<br>Fecha:

INSTRUCCIONES:

1) Contabilice 01 error por cada respuesta incorrecta y anótelo en la columna "B".
2) El número máximo de errores permitidos en cada ítem está indicado en la columna "A"
3) Multiplique el NÚMERO DE ERRORES por el valor de cada ítem señalado en la columna "D"
4) Registre el resultado de la multiplicación en la columna "F"
5) Sume los puntajes calculados de cada Ítem de la columna "F" para obtener el "PUNTAJE TOTAL CALCULADO" y anótelo en la celda correspondiente.
6) Un PUNTAJE TOTAL CALCULADO igual a 0 significa que todos los ítems fueron respondidos correctamente. Un PUNTAJE TOTAL CALCULADO igual a 28 significa que se alcanzó el MÁXIMO DE ERRORES PERMITIDOS para la prueba y que todos los ítems fueron respondidos incorrectamente.
7) Cualquier error de puntuación que sumado dé entre 0 a 6, se encuentra dentro de los límites normales. Puntuaciones de error mayores que 10 son consistentes con Demencia, de acuerdo con Katzman *et al*.

N° de Ítem	ID Columnas	A	B	C	D	E	F
	ÍTEM	MÁXIMO DE ERRORES PERMITIDOS	NÚMERO DE ERRORES	Operación	VALOR del ITEM		PUNTAJE CALCULADO
1	¿En qué año estamos ahora?	1		x	4	=	
2	¿En qué mes estamos ahora?	1		x	3	=	
FRASE DE MEMORIA	Repita esta frase después de mí:<br>*Calle Circunvalación #42, Barrio Miraflores, Lima.*						
3	¿Qué hora es aproximadamente? (dentro de la hora)	1		x	3	=	
4	Cuente hacia atrás del 20 al 1	2		x	2	=	
5	Diga los meses del año en orden inverso (del último al primer mes)	2		x	2	=	
6	Repita la Frase de Memoria	5		x	5	=	
PUNTAJE TOTAL CALCULADO							

La puntuación máxima de error para la prueba=28

Referencia:
Katzman R, Brown T, Fuld P, Peck A, Schechter R, Schimmel H. "Validation of a short Orientation-Memory-Concentration Test of cognitive impairment." Am J Psychiatry 1983;140:734-9. Copyright 1983, American Psyciatric Association.

Comentario
La prueba Blessed Orientación-Memoria-Concentración de seis puntos (BOMC) fue desarrollada por Katzman, Brown, Fuld, *et al*. (1983) de la prueba más larga Blessed Información-Memoria-Concentración de 29 ítems (BIMC), (Blessed, Tomlinson, y Roth, 1968). Katzman y sus colegas seleccionaron 6 de los 29 ítems BIMC originales, basados en una serie de análisis estadísticos. La puntuación correlaciona altamente (r = 0,92) con la escala completa y fue casi tan sensible como la prueba larga.

Compilado de www.strokecenter.org y www.stanford.edu/search/

CUESTIONARIO DE INGRESO AL TRATAMIENTO

INSTRUCCIONES: Por favor indique el grado de conformidad o informidad que tenga con respecto a las siguientes afirmaciones, indicando en el espacio en blanco el número que mejor refleje su opinión. Recuerde que no hay respuestas correctas o incorrectas y que sus respuestas son confidenciales.

Utilice la siguiente escala para hacer su evaluación						
1	2	3	4	5	6	7
Totalmente en desacuerdo			Me es indiferente			Totalmente de acuerdo

Afirmaciones		Sub-escala
1	Decidí entrar en un programa de tratamiento porque estaba interesado en obtener ayuda.	Regulación Autónomo / Regulación identificada
2	Decidí entrar en un programa de tratamiento porque realmente quiero hacer cambios en mi vida.	Regulación introyectada
3	Decidí entrar en un programa de tratamiento porque es muy importante para mí lidiar con mi problema de abuso a sustancias.	Regulación externa
4	Planeo pasar por un programa de tratamiento porque realmente quiero dejar de abusar del alcohol y las drogas	Regulación Autónomo / Regulación identificada
5	No estaré satisfecho conmigo a menos que mi problema de abuso a sustancias esté bajo control. Es por esto que decidí entrar en un programa de tratamiento.	Regulación introyectada
6	Tengo la intención de seguir adelante con el tratamiento porque voy a estar avergonzado de mí mismo si no lo hago.	Regulación externa
7	Si permanezco en el tratamiento tal vez sea porque, si no continúo, podría sentirme como un fracasado.	Regulación introyectada
8	Planeo pasar por un programa de tratamiento porque realmente quiero dejar de abusar del acohol y las drogas.	Regulación Autónomo / Regulación identificada
9	Tal vez permanezco en el tratamiento porque otros pueden enojarse conmigo si no lo hago	Regulación externa
10	Estoy en tratamiento porque otras personas me han presionado para estar aquí.	Regulación Autónomo / Regulación identificada
11	Acepté seguir un programa de tratamiento porque, si no sigo todas las reglas, me meteré en problemas con mis amigos y mi familia	Regulación introyectada
12	Acepté seguir un programa de tratamiento porque me presionaron a venir.	Regulación externa

Indice De Severidad De Adicción 5ta Edición

Versión Compatible Al Censo Clínica/De Entrenamiento

(Patrocinado por: Sistemas Quickstart, Inc.)

Harold C. Urshel, III, M.D.

Jacqueline Blair

A. Thomas McLellan, Ph.D.

Traducción: José D. Morales

<u>Introducción al ISA:</u>
Siete áreas de problemas potenciales: Médico, Empleo/Sustento, Alcohol, Drogas, Legal, Familiar/Social y Psicológico. Todos los clientes reciben esta misma entrevista <u>estándar.</u> Toda la información adquirida será <u>confidencial.</u>
Discutiremos dos períodos de tiempo:
1. Durante los últimos 30 días
2. Durante toda su vida

<u>Escala de Evaluación del Paciente:</u> La participación del paciente es esencial. En cada área utilize la escala a continuación para describir cuánto le ha afectado cualquiera de los problemas en cada sección, también se le preguntará, cuán importante le es recibir tratamiento en el área discutida.

La Escala es: 0-Nada en absoluto
1-Poco o levemente
2-Moderadamente
3-Considerablemente
4-Extremadamante

Si se siente incómodo contestando, no tiene que contestar.
!Favor de proveer información correcta!
Recuerde: Esto es una entrevista, no un examen.

<u>Instrucciones al Entrevistador:</u>
1. No deje espacios en blanco.
2. Anote todos los comentarios (si otra persona lee este ISA, ella debe de tener una idea, relativamente completa de la percepción del cliente y sus problemas).
3. X=La pregunta no se contestó.
   N=La pregunta no se aplica.
4. Descontinue la entrevista si el cliente miente en dos secciones o más.
5. Cuando anote comentarios, por favor anote el número de la pregunta .
6. Instrucciones y notas de codificación son precedidas con ">"

<u>Escala del Entrevistador:</u>

0-1	=	No hay problema
2-3	=	Un problema mínimo
4-5	=	Un problema moderado
6-7	=	Un problema severo
8-9	=	Un problema extremo

<u>REGLA DEL MEDIO TIEMPO</u>**:** Si una pregunta se refiere al número de meses, redondee períodos de 14 días o más a un mes. Redondee períodos de 6 meses o más a un año.

<u>EVALUACION DE VERACIDAD</u>:
>Los últimos dos artículos en cada sección
>No exagere
>La negación no constituye falsedad
>Falsedad = contradicción de información obvia .

<u>SONDEE Y ANOTE MUCHOS COMENTARIOS!</u>

<u>CATEGORIAS DE HOLLINGSHEAD</u>:
1. Altos ejecutivos, profesionales, dueños de grandes empresas.
2. Gerentes, profesionales tales como: enfermeras, optómetras, farmacéuticos, trabajadores sociales, profesores.
3. Personal asministrativo, gerentes, profesionales, dueños de pequeños negocios, e.j., negocios de panadería, automóviles, grabación, plomería y floristería. Decoradores, actores, periodistas, agentes de viaje.
4. Trabajadores de oficina y vendedores, técnicos, trabajadores de comercios pequeños (cajeros, contadores, oficinistas, delineantes, cronometradores, secretarios.)
5. Trabajadores diestros que normalmente reciben entrenamiento previo (panadero, barbero, jefe de cocina, electricista, bombero, operario, mecácnico, pintor, reparador, sastre, soldador, policía, plomero).
6. Semi-diestro (ayudante de hospital, camarero de cantina, conductor de autobús, cocinero, guardia, camarero, soldador, operador de máquinas).
7. Trabajadores no diestros (trabajadores de servicios doméstico, ayudante de construcción, obrero sin especialización, maletero, desempleados.
8. Amas de casa
9. Estudiantes, personas con incapacidades, personas sin profession.

<u>LISTA DE DROGAS COMUNMENTE USADAS</u>:

Alcohol:	Cerveza, vino, licor
Opiáceos:	Analgésicos = Morfina, Dilaudid, Demerol, Percocet, Darvon, Talwin, Codeína, Tylenol 2,3,4, Jarabes = Robitussin, Fentanyl
Barbitúricos:	Nebutal, Seconal, Tuinol, Amytal, Pentobarbital, Secobarbital, Phenobarbital, Fiorinol
Sedativos:	Benzodiazepines = Valium, Librium, Ativan, *(Hipnosedantes,* Serax, Tranxene, Dalmane, Halcion, Xanax, *Tranquilizantes)* Miltown. Otros = Chloral Hydrate (Noctex), Quaaludes
Cocaína:	Cocaína en cristal, "Crack" y Cocaína en forma de "Piedras"
Anfetaminas:	Monstro, Crank, Benzadrine, Dexedrine, Ritalin, Preludin, Methanfetamina
Cannabis:	Marijuana, Hashish
Alucinógenicos:	LSD, "Hongos o Mushrooms" (Psílocybin), Mescaline, Peyote, PCP (Phencyclidine) "Angel Dust", "Ecstacy".
Inhalables:	Nitrous Oxide, Amyl Nitrate (Whippits, Poppers), Goma de pegar/Cola, Solventes (Gasolina, etc.)
Solamente anote el uso de:	Antidepresivos<br>Medicamentos para las úlseras = Zantac, Tagament<br>Medicamentos del asma = Inhalador de Ventoline, Theodur<br>Otros Medicamentos: Antipsicóticos, Lithium

<u>INSTRUCCIONES RELACIONADAS AL USO DE DROGAS Y ALCOHOL:</u>
Las siguientes preguntas se refieren a dos períodos de tiempo: durante los últimos 30 días y durante toda su vida. Durante toda su vida se refiere al período antes de los últimos 30 días.
> Preguntas de 30 días sólo requieren el número de días de uso.
> Uso durante toda la vida se refiere a uso durante períodos prolongados.
> Uso regular = 3 veces o más por semana, borracheras o uso irregular y problemático donde las actividades diarias son comprometidas.
> Uso de alcohol hasta la embriaguez no necesariamente significa "borracheras", use la frase "sentir los efectos", etc.
  Por regla general, 5 o más bebidas en un día o 3 bebidas seguidas se define como embriaguez.
> Cómo preguntar:
  > ¿Cuántos días de los últimos 30 ha usado...?
  > ¿Cuántos años en su vida ha usado...con regularidad?

Indice De Severidad De Adicción, 5ta Edición

INFORMACION GENERAL

Identificación: ☐☐☐☐☐☐☐☐☐☐☐

Número de Seguro Social: ☐☐☐☐☐☐☐☐☐

Fecha de Admisión: ☐☐ / ☐☐ / ☐☐

Fecha de la Entrevista: ☐☐ / ☐☐ / ☐☐

Hora comenzada: HORA:MINUTOS ☐☐ : ☐☐

Hora terminada: HORA:MINUTOS ☐☐ : ☐☐

Clase: 1. Admisión 2. Seguimiento ☐

Código de Contacto: 1. En persona 3. Por correo 2. Por teléfono (Es necesario que el ISA de admisión sea administrado personalmente.) ☐

Sexo: 1. Masculino 2. Femenino ☐

Número del Episodio de Tratamiento: ☐☐

Número Código del Entrevistador: ☐☐☐☐

Nobre del Entrevistador: ______________________

NOMBRE Y DIRECCION DEL PACIENTE:

____
Nombre

____
Dirección 1

____
Dirección 2

____
Ciudad Estado Zona Postal

____
Número telefónico corriente

Indice De Severidad										
Problemas	0	1	2	3	4	5	6	7	8	9
Médico										
Empleo										
Alcohol										
Drogas										
Legal										
Familiar										
Psicológico										

PREGUNTAS GENERALES:

14. ¿Cuánto tiempo lleva viviendo en esta dirección? ☐☐ / ☐☐ (Años/Meses)

15. ¿Es propiedad suya o de un familiar? ☐ 0-No 1-Sí

16. Fecha de nacimiento (mes/día/año) ☐☐ / ☐☐ / ☐☐

17. ¿De qué raza se considera? ☐
    1. Blanco (no Hispano) 6. Hispano- Mexican
    2. Negro (no Hispano) 7. Hispano- Puerto Rican
    3. Indio/Nativo de America 8. Hispano- Cuban
    4. Indio/Nativo de Alaska 9. Hispano Otro
    5. Asiático/Isleño del Pacífico

18. ¿Tiene alguna preferencia religiosa? ☐
    1. Protestante 3. Judía 5. Otra
    2. Católica 4. Islámica 6. Ninguna

19. ¿Ha estado en un ambiente controlado durante los últimos 30 días? ☐
    1. No 4. Tratamiento Médico
    2. Encarcelado 5. Tratamiento Psiquiátrico
    3. Tratamiento de Drogas/Alcohol 6. Otro:______________

    >Un lugar sin acceso a drogas/alcohol

20. ¿Cuántos días? ☐☐
    > "NN" si la pregunta 6 es No. Se refiere al número total de días detenido durante los últimos 30 días.

RESULTADOS DE EXAMENES ADICIONALES

____

____

____

____

____

COMENTARIOS SOBRE INFORMACION EN GENERAL (Anote el número de la pregunta con sus notas)

____

____

____

____

____

Número de RMA _______________

Indice De Severidad De Adicción, 5ta Edición

ESTADO MEDICO

1. ¿Cuántas veces ha estado hospitalizado por problemas médicos? ☐☐
   >Incluya sobredosis y D.T. (Delirio Tremens). Excluya desintoxicaciones y tratamiento psiquiátrico, de drogas/alcohol y partos (sin complicaciones). Apunte la cantidad de hospitalizaciones de la noche a la mañana por problemas médicos.

2. ¿Cuánto tiempo hace que estuvo hospitalizado por un problema físico: ☐☐ / ☐☐
   >Si no huvo hospitalizaciones en el #1, marque "NN".

3. ¿Tiene algún problema médico crónico que continua interfiriendo con su vida? 0-No 1-Sí ☐
   Sí la contestación es "Sí", especifique en los comentarios
   >Una condición médica crónica es una condición física seria o médica que exije tratamiento constante (e.j., medicamento, régimen de dieta) impidiendo el completo aprovechamiento de sus habilidades.

4. ¿Toma algún medicamento con regularidad, recetado para un problema físico? ☐
   Sí la contestación es "Sí", especifique en los comentarios. 0-No 1-Sí
   >Medicamento recetado por un doctor en medicina para una condición médica, excluyendo medicinas psiquiátricas. Incluya medicamentos recetados, aunque no se lo esté tomando. El intento es de verificar problemas médicos crónicos.

5. ¿Recibe una pensión por alguna incapacidad física? 0-No 1-Sí ☐
   >Incluya la compensación por desempleo, excluya incapacidad psiquiátrica.
   Sí la contestación es "Sí", especifique en los comentarios.

6. ¿En los últimos 30 días, cuántos días ha tenido problemas médicos? ☐☐
   >No incluya enfermedades directamente causadas por drogas/alcohol. Incluya la gripe, resfriados, etc. y enfermedades serias, relacionadas con el uso de drogas/alcohol las cuales continuarían aunque estuviera abstemio (e.j., cirrosis del hígado, abscesos causados por jeringillas, etc.).

Para las preguntas #7 y #8 solicite al paciente que utilize la escala de Evaluación del Paciente.

7. ¿En los últimos 30 días, cuánto le ha preocupado o molestado estos problemas médicos? ☐
   >Limite la respuesta a los días problemáticos del #6.

8. ¿Cuán importante le es recibir tratamiento para estos problemas médicos? ☐
   >Se refiere a la necesidad de tratamiento médico **adicional** por parte del paciente.

Evaluación de Severidad del Entrevistador

9. ¿Cómo eválua la necesidad del paciente de recibir tratamiento médico? ☐
   >Refiérase a la necesidad del paciente de recibir tratamiento médico adicional.

Evaluación de Veracidad del Paciente

La información anterior está significativamente mal representada por:

10. ¿Intención de parte del paciente? 0-No 1-Sí ☐

11. ¿Incapacidad del paciente para comprender? 0-No 1-Si ☐

(Versión Compatible Al Censo Clínica/De Entrenamiento)

COMENTARIOS MEDICOS

(Anote el número de la pregunta con sus notas)

____
____
____
____
____
____
____
____
____
____
____
____
____
____
____
____
____
____
____
____
____
____
____
____
____

Número De RMA _______________

ESTADO DE EMPLEO / SUSTENTO

1. Nivel de educación completada: ☐☐ / ☐☐ Años Meses
>Diploma de equivalencia general = cuarto año de escuela superior, anote en los comentarios.
>Solamente incluya educación oficial.

2. Educación técnica o adiestramiento completado: ☐☐ Meses
>Solamente adiestramiento oficial.
Para adiestramiento militar, solamente incluya adiestramiento que se pueda utilizar en la vida civil, e.j., electrónica versus artillería.

3. ¿Tiene una profesión, oficio o destreza? ☐ 0-No 1-Sí
>Una destreza transferible/empleable adquirida por adiestramiento.
Si la contestación es "Sí" (especifique)______________

4. ¿Tiene una licencia válida de conducir? ☐ 0-No 1-Sí
>Licencia válida; no suspendida o revocada.

5. ¿Tiene un automóvil disponible? ☐ 0-No 1-Sí
>Si la contestación del #4 es "No", marque "NN" .
No requiere posesión, solamente disponibilidad con regularidad.

6. ¿Cuál es la cantidad de tiempo más largo que ha estado empleado en un trabajo de tiempo completo, durante todasu vida? ☐☐ / ☐☐ Años Meses
>Tiempo completo = 35 o más horas semanalmente; no se refiere necesariamente al empleo más reciente.

7. ¿Oficio (último o) principal? ☐
(Especifique)_____________________________
(Use las categorías de Hollingshead en la página de referencia)

8. ¿Alguien contribuye de cualquier manera a su sustento? 0-No 1-Si ☐
>El paciente esta recibiendo el sustento con regularidad (e.j., dinero, comida,vivienda) de parte de familiares/amistades. Incluya contribuciones del cónyuge; excluya mantenimiento institucional.

9. ¿Esto constituye la mayoría de su sustento? >Si el # 8 es "No", marque "N". 0-No 1-Sí ☐

10. ¿Patrón usual de empleo durante los últimos 3 años? ☐

1. A tiempo completo (35 o más horas)	5. Servicio militar
2. A tiempo parcial (horas regulares)	6. Jubilado/Incapacitado
3. A tiempo parcial (horas irregulares)	7. Desempleado
4. Estudiante	8. En un ambiente controlado

>La respuesta debe representar la mayoría de los últimos 3 años, no la selección más reciente. Si hay épocas iguales para más de una categoría, escoja la que mejor represente la situación más reciente.

11. ¿Cuántos días le han pagado por su trabajo durante los últimos 30 días? ☐☐
>Incluya empleo "por debajo de la mesa", días de enfermedad pagados y vacaciones.

COMENTARIOS DE EMPLEO / SUSTENTO

(Anote el número de la pregunta con sus notas)

Número de RMA _______________

EMPLEO / SUSTENTO (Continuación)

¿Cuánto dinero ha recibido durante los últimos 30 días por lo siguiente:

12. Empleo? ☐☐☐☐☐
>Incluya dinero pagado por " debajo de la mesa".

13. Compensación por desempleo? ☐☐☐☐☐

14. Asistencia pública? ☐☐☐☐☐
>Incluya cupones de alimentos y dinero para transportación, provisto por una agencia para ir y regresar a un centro de tratamiento.

15. Pensiones, beneficios o Seguro Social? ☐☐☐☐☐
>Incluya incapacidad, jubilación, pensiones, beneficios de veteranos, indemnización de empleo y SSI.

16. Esposo(a), familia o amigos? ☐☐☐☐☐
>Dinero por gastos personales, (e.j., ropa), incluya fuentes imprevistas de ingreso (e.j., apuestas). Solamente anote pagos en dinero en efectivo. Incluya dinero improvisto golpes de suerte, préstamos, apuestas, herencias, declaraciones de ingresos, etc.

17. Ilegalmente? ☐☐☐☐☐
>Dinero en efectivo recibido por venta de drogas, robo, venta de artículos hurtados, apuestas, prostitución, etc. No transfiera dinero en efectivo, el valor de drogas ofrecidas como parte del pago.

18. Cuántas personas dependen de usted para la mayoría de su sustento (comida, vivienda, etc.)? ☐☐
>Tiene que ser que dependa del paciente con regularidad, no incluya pensión del cónyuge y pensión alimenticia, no incluya al paciente o a la pareja si esta es autosuficiente.

19. ¿En los últimos 30 días, cuántos días ha tenido problemas de empleo? ☐☐
>Incluya la inhabilidad de conseguir empleo, si está activamente buscando empleo o problemas que tenga en su trabajo actual, el cual está poniendo a riesgo.

Para las preguntas #20 y #21 solicite al paciente que utilize la escala de Evaluación del Paciente.

20. ¿En los últimos 30 días cuánto le ha preocupado o molestado estos problemas de empleo? ☐
>Si el paciente ha estado encarcelado o detenido durante los últimos 30 días, no tuvo "problemas de empleo". En ese caso, una respuesta con "N" se indica.

21. ¿Cuán importante le es recibir consejería en relación a estos problemas de empleo? ☐
>Las evaluaciones del paciente en las preguntas #20 y #21 se refieren a la pregunta #19. Enfatize que esto se refiere a la ayuda en encontrar o prepararse para un trabajo, no el acto de darle un trabajo.

Evaluación de Severidad del Entrevistador

22. ¿Cómo eválua la necesidad del paciente de recibir consejería por sus problemas de empleo? ☐

Evaluación de Veracidad del Paciente

La información anterior esta significativamente mal representada por..

23. ¿Intención del paciente? 0-No 1-Sí ☐

24. ¿Incapacidad del paciente para comprender? 0-No 1-Sí ☐

COMENTARIOS DE EMPLEO / SUSTENTO

(Anote el número de la pregunta con sus notas)

____
____
____
____
____
____
____
____
____
____
____
____
____
____
____
____
____
____
____
____
____

Número de RMA _______________

USO DE DROGAS / ALCOHOL

Vías de administración:
1. Oral 2. Nasal 3. Fumado 4. Inyección (No intravenosa) 5. Inyeccion Intravenosa. Anote la vía de uso acostumbrado o corriente. Para más de una vía, escoja la más severa. Las vías estan enlistadas desde la menos severa a la más severa.

		Durante los últimos 30 Días	Durante la vida (Años)	Via de administración
1	Alcohol (cualquier uso)			
2	Alcohol (ha embriaguez)			
3	Heroína			
4	Metadona			
5	Otros Opíaceos/Analgésicos			
6	Barbitúricos			
7	Sedativos/Hipnosedantes/ Tranquilizantes			
8	Cocaína			
9	Anfetaminas			
10	Cannabis			
11	Alucinógenos			
12	Inhalantes			
13	Más de una sustancia al día (incluyendo alcohol)			

14 ¿De acuerdo al entrevistador, cuál sustancia constituye el problema principal?
>El entrevistador debe de determinar la droga de abuso principal. Codifique el número al lado de la droga en las preguntas #1-#12, "00" = no hay problema, "15" = alcohol y una o más drogas "16" = más de una droga Preguntele al paciente si tiene duda.

15. ¿Cuánto tiempo duró la última abstinencia voluntaria de esta sustancia principal? Meses
>La última abstinencia que ha durado por lo meno un mes; no necesariamente la más larga. Períodos de hospitalización o encarcelación no cuentan. Períodos del uso de antabuse, metadona o naltrexone durante la abstinencia cuentan. Solamente indique períodos de 30 días o más.
>00 = nunca abstemio.

16. ¿Cuántos meses hace que terminó esta abstinencia?
> Si la pregunta #15 = 00, #16 = NN.;
marque "00" si todavía está abstemio

17. ¿Cuántas veces ha sufrido.. DT's por alcohol?
>***Delerium Tremens (DT's):*** Suceden 24-48 horas después de la última bebida, o disminución significativa del consumo de alcohol: temblores, desorientación severa, fiebre, alucinaciones que usualmente requieren atención médica.

18. Sobredosis de drogas?
>*Sobredosis (SD):* Requiere intervención de alguien para recapacitar, el dormir no disminuye los efectos, incluya intentos de suicidio por sobredosis.

COMENTARIOS DE DROGAS / ALCOHOL

(Anote el número de la pregunta con sus notas)

Número De RMA _______________

DROGAS / ALCOHOL (Continuación)

19. ¿Cuántas veces en su vida ha sido tratado por abuso de acohol? ☐☐
>Incluya desintoxicación, domicilios de medio camino, consejería como paciente interno o externo, y A.A. (si 3 o más reuniones durante el período de un mes).

21. ¿Cuántas de estas han sido solamente desintoxicaciones de acohol? ☐☐
>Si la #19 = "00", luego la #21 = "NN"

23. ¿Cuánto dinero diría que ha gastado durante los últimos 30 días en alcohol? ☐☐☐☐☐
>Solamente incluya dinero en efectivo gastado.
¿Cuál es la carga financiera causada por drogas/alcohol?

20. ¿Cuántas veces en su vida ha sido tratado por abuso de drogas? ☐☐
>Incluya desintoxicación, domicilios de medio camino, consejería como paciente interno o externo, y N.A. (si 3 o más reuniones durante el período de un mes).

22. ¿Cuántas de estas han sido solamente desintoxicaciones de drogas? ☐☐
>Si la #20 = "00", luego la #22 = "NN"

24. ¿Cuánto dinero diría que ha gastado durante los últimos 30 días en drogas? ☐☐☐☐☐
>Solamente incluya dinero en efectivo gastado.
¿Cuál es la carga financiera causada por drogas/alcohol?

25. ¿Cuántos de los últimos 30 días ha sido tratado como paciente externo por el uso de drogas/alcohol? (Incluya a N.A. o A.A) ☐☐

26. ¿En los últimos 30 días, cuantos días ha tenido problemas de alcohol? ☐☐
>Solamente incluya: Ansiedad, síntomas de abstinencia, efectos negativos a causa del uso o deseos de descontinuar el uso, pero inhabilidad de hacerlo.

Para las preguntas #28 y #30 solicite al paciente que utilize la escala de Evaluación del Paciente.
El paciente está evaluando la necesidad de tratamiento adicional por el abuso de sustancias.

28. ¿En los últimos 30 días cuánto le han preocupado o molestado estos problemas de alcohol? ☐

30. ¿Cuán importante les, recibir tratamiento para estos problemas de alcohol? ☐

27. ¿En los últimos 30 días, cuantos días ha tenido problemas de drogas? ☐☐
>Solamente incluya: Ansiedad, síntomas de abstinencia, efectos negativos a causa del uso o deseos de descontinuar el uso, pero inhabilidad de hacerlo.

Para las preguntas #29 y #31 solicite al paciente que utilize la escala de Evaluación del Paciente.
El paciente está evaluando la necesidad de tratamiento adicional por el abuso de sustancias.

29. ¿En los últimos 30 días cuánto le han preocupado o molestado estos problemas de drogas? ☐

31. ¿Cuán importante les, recibir tratamiento para estos problemas de drogas? ☐

Evaluación de Severidad del Entrevistador

¿Cómo eválua la necesidad del paciente para recibir tratamiento:

32. Para sus problemas de alcohol? ☐

33. Para sus problemas de drogas? ☐

Evaluación de Veracidad del Paciente

La información anterior está significativamente mal representada por:

34. ¿Intención del paciente? 0-No 1-Sí ☐

35. ¿Incapacidad del paciente para comprender? 0-No 1-Sí ☐

COMENTARIOS SOBRE DROGAS / ALCOHOL

(Anote el número de la pregunta con sus notas)

____

____

____

____

____

____

____

____

____

____

____

____

____

____

____

____

____

____

Número De RMA _______________

ESTADO LEGAL

1. ¿Esta admisión fue iniciada o sujerida por el sistema judicial? 0-No 1-Sí ☐
   >Juez, oficial de probatoria/libertad condicional, etc.

2. ¿Está en libertad condicional o probatoria? ☐
   >Anote la duración y nivel en los comentarios. 0-No 1-Sí

¿Cuántas veces en su vida ha sido arrestado y cargado con lo siguiente?

03 Ratería/vandalismo	☐☐	10 Asalto	☐☐
04 Violacion deProbatoria/ libertad condicional	☐☐	11 Incendio provocado	☐☐
05 Cargos de drogas	☐☐	12 Violación sexual	☐☐
06 Falsificación de documentos	☐☐	13 Homicidio/Homicidio sin premeditación	☐☐
07 Delitos de armas	☐☐	14 Prostitución	☐☐
08 Robo con allanamiento de morada, hurto, entrada por fuerza	☐☐	15 Desacato a la corte	☐☐
09 Robo	☐☐	16 Otro delito:____________	☐☐

>Incluya el número total de cargos, no solamente convicciones. No incluya crimenes juveniles (antes de los 18 años) a menos que fuera acusado como un adulto. Incluya solamente cargos formales.

17. ¿Cuántos de estos cargos resultaron en convicciones? ☐☐
    >Si #03 - #16 = "00", marque "NN"
    >No incluya delitos menores en las preguntas 18-20 más abajo.
    >Convicciones incluyen: Multas, libertad condicional, encarcelaciones, sentencias suspendidas y declaraciones de culpabilidad.

¿Cuántas veces en su vida ha sido acusado por lo siguiente?

18. ¿Desorden público, holgazanería o intoxicación publica? ☐☐

19. ¿Manejar en estado de embriaguez? ☐☐

20. ¿Infracciones mayores de tránsito? ☐☐
    >Violaciones de tránsito: manejando a exceso de velocidad, imprudentemente o no poseyendo licencia de conducir, etc.

21. ¿Durante toda su vida, cuántos meses ha estado encarcelado? ☐☐ Meses
    >Si ha estado encarcelado 2 semanas o más, se redondea a un mes. Anote la cantidad total de meses encarcelado.

22. ¿Cuánto tiempo duró su último encarcelamiento? ☐☐ Meses
    >Marque "NN" si nunca estuvo encarcelado.

23. ¿Cuál fue el cargo? ☐☐
    >Use el código #03 - #16, #18 - #20. Si hay cargos múltiples, use el código más severo. Marque "NN" si nunca ha estado encarcelado.

24. ¿Actualmente está esperando acusaciones, juicio o sentencia? ☐ 0-No 1-Sí

25. ¿Por cuál violación? ☐☐
    >Se refiere a la pregunta #24, si hay varios, escoja el más grave. No incluya casos civiles, a menos que un delito criminal haya sido envuelto.

26. ¿Cuántos días de los últimos 30 ha estado detenido o encarcelado? ☐☐
    >Incluya el haber sido detenido y puesto en libertad el mismo día.

COMENTARIOS SOBRE EL ESTADO LEGAL

(Anote el número de la pregunta con sus notas)

ESTADO LEGAL (Continuación)

27. ¿Durante los últimos 30 días, cuántos días ha estado envuelto en actividades ilegales para su propio provecho? ☐☐
>Incluya posesión simple de drogas. Incluya ventas de drogas, prostitución,venta de artículos robados, etc. Se puede comprobar con la pregunta #17 en la sección de Empleo/Sustento.

Para las preguntas #28 y #29 solicite al paciente que utilize la Escala de Evaluación del Paciente.

28. ¿Cuán serios son estos problemas legales? ☐
>Excluya problemas civiles.

29. ¿Cuán importante le es recibir consejería o ser referido sobre sus problemas legales? ☐
>El paciente está evaluando la necesidad de obtener referidos adicionales para su defensa en cargos criminales.

Evaluación de Severidad del Entrevistador

30. ¿Cómo evalúa la necesidad del paciente de recibir servicios legales o consejería? ☐

Evaluación de Veracidad del Paciente

¿La información anterior está significativamente malrepresentada por:

31. Intención del paciente? ☐ 0-No 1-Sí

32. Incapacidad del paciente para comprender? ☐ 0-No 1-Sí

COMENTARIOS SOBRE EL ESTADO LEGAL

(Anote el número de la pregunta con sus notas)

____

____

____

____

____

____

____

____

____

____

____

____

____

____

HISTORIA FAMILIAR

¿Tiene familiares por sangre que han tenido lo que llamaría un problema significativo con bebidas alcohólicas, uso de drogas o problemas psiquiátricos? **Uno que provocó o hubiese provocado tratamiento.**

Lado Materno	Alc.	Dro.	Psiq.	Lado Paterno	Alc.	Dro.	Psiq.	Hermanos	Alc.	Dro.	Psiq.
Abuela	☐	☐	☐	Abuela	☐	☐	☐	Hermano/Hermana 1	☐	☐	☐
Abuelo	☐	☐	☐	Abuelo	☐	☐	☐	Hermano/Hermana 2	☐	☐	☐
Madre	☐	☐	☐	Padre	☐	☐	☐	Hermano/Hermana 3	☐	☐	☐
Tía	☐	☐	☐	Tía	☐	☐	☐	Hermano/Hermana 4	☐	☐	☐
Tío	☐	☐	☐	Tío	☐	☐	☐				

0 = Claramente No por todos los familiares en esa categoría X = Incierto o desconocido
1 = Claramente Sí por todos los familiares en esa categoría N = Nunca hubo un familiar
>En casos donde hay más de una persona por categoría, anote el más severo. Acepte la opinión del paciente para estas preguntas

COMENTARIOS SOBRE LA HISTORIA FAMILIAR

____

____

____

Número De RMA _______________

FAMILIA / RELACIONES SOCIALES

1.Estado Civil:

1- Casado 3- Viudo 5- Divorciado
2- Casado por segunda vez 4- Separado 6- Nunca casado
>Casado bajo ley consensual = #1, especifique en los comentarios

2. ¿Cuánto tiempo lleva en este estado civil (#1)?
>Si nunca ha estado casado, anote la cantidad de tiempo desde los 18 años.
Años / Meses

3. ¿Está satisfecho con esta situación?
Satisfecho = generalmente contento con la situación.
Se refiere al #1 y #2.

4. Arreglos normales de vivienda durante los últimos 3 años:
1-Con la pareja sexual e hijos
2-Solamente con la pareja sexual
3-Solamente con sus hijos
4-Con padres
5-Con familiares
6-Con amistades
7-Solo
8-En un ambiente controlado
9-Sin arreglo estable
>Escoja el arreglo que mejor represente los últimos 3 años.
Si hay una división exacta en el tiempo, anote el arreglo más reciente.

5. ¿Cuánto tiempo ha vivido bajo este arreglo?
>Si con los padres, desde los 18 años.
>Codifique años y meses viviendo en los arreglos de la #4.
Años / Meses

6. ¿Está satisfecho con estos arreglos?
0-No 1-indiferente 2-Sí

¿Vive con alguien que:

7. Actualmente tiene un problema con alcohol? 0-No 1-Sí

8. Usa drogas sin receta? 0-No 1-Sí
(o los abusos prescribieron drogas)

9. ¿Con quién pasa la mayor parte de su tiempo libre? (1-Familia 2-Amistades 3-Solo)
>Si una novia/novio es considerado como familiar por el paciente, el paciente está obligado a referirse a ellos como familiar durante toda la sección y no como amistad. Familiares no se consideran "amigos".

10. ¿Está satisfecho empleando su tiempo libre de esta manera? 0-No 1-indiferente 2-Sí
>Una respuesta satisfactoria tiene que indicar que la persona generalmente le gusta la situación. Refiriéndose a la pregunta #7.

11. ¿Cuántos amigos íntimos tiene?
>Enfatize la palabra **íntimo** y excluya miembros familiares. Estas son relaciones "recíprocas" o de apoyo mutuo.

. Diría que ha tenido relaciones íntimas y recíprocas con las siguientes personas:

12. Madre
13. Padre
14. Hermanos(as)
15. Pareja sexual/Esposo(a)
16. Hijos
17. Amigos

0=Claramente No por todos en la categoría X=Incierto o desconocido
1=Claramente Sí por todos en la categoría N=Nunca huvo un familiar
>Por recíproco, se interpreta "que usted haría todo lo posible por ayudarlos y ellos harían lo mismo por udsted."

COMENTARIOS SOBRE LA FAMILIA / RELACIONES SOCIALES

(Anote el número de la pregunta con sus notas)

Número de RMA _______________

FAMILIA / RELACIONES SOCIALES (Cont.)

¿Ha tenido periodos importantes en los que has experimentado dificultad en las relaciones con las sigientes personas:

0-No 1-Sí		En los últimos 30 días	En su vida
18.	Madre	☐	☐
19.	Padre	☐	☐
20.	Hermanos(as)	☐	☐
21.	Pareja Sexual/Esposo(a)	☐	☐
22.	Hijos	☐	☐
23.	Otros familiares significativos (especifique)_______________	☐	☐
24.	Amigos íntimos	☐	☐
25.	Vecinos	☐	☐
26.	Compañeros de trabajo	☐	☐

"Problemas serios" son problemas que han puesto la relación en peligro. Un "problema" requiere contacto de una forma, por teléfono o en persona.

¿Alguna de las personas (en preguntas #18 - #26) ha abusado de ud:

0-No 1-Sí		Durante los últimos 30 días	En su vida
27.	Emocionalmente?<br>>Te hizo mal através de palabras crueles.	☐	☐
28.	Físicamente?<br>>Te ha causado daño físico.	☐	☐
29.	Sexualmente?<br>>Actos/contactos sexuales forzados.	☐	☐

¿En los últimos 30 días, cuántos días ha tenido conflictos graves:

30. Con su familia? ☐☐

Para las preguntas #32 y #34 solicite al paciente que utilize la Escala de Evaluación del Paciente.

¿En los últimos 30 días cuánto le ha preocupado o molestado sus:

32. Problemas familiares? ☐

¿Cuán importante le es recibir tratamiento o consejería para sus:

34. Problemas familiares? ☐
>El paciente está evaluando la necesidad de su familia de consejería para resolver problemas familares, y no determinando la disposición de su familia para participar en consejería.

¿En los últimos 30 días, cuántos días ha tenido conflictos graves:

31. Con otras personas (excluyendo a familiares)? ☐☐

Para las preguntas #33 y #35 solicite al paciente que utilize la Escala de Evaluación del Paciente.

¿En los últimos 30 días cuánto le ha preocupado o molestado sus:

33. Problemas sociales? ☐
(Con personas fuera de su familia).

¿Cuán importante le es recibir tratamiento o consejería para sus:

35. Problemas Sociales? ☐
>Excluya la necesidad del paciente para solicitar tratamiento por tales problemas sociales como soledad, inhabilidad de socializar e insatisfacción con las amistades. La evaluación del paciente debe referirse a insatisfacción, conflictos u otros problemas serios. Excluya problemas que hubisen sidos eliminados si el paciente no estuviera abusando drogas.

Evaluación de Severidad del Entrevistador

36. ¿Cómo evalúa la necesidad del paciente de recibir consejería familiar o social? ☐

Evaluación de Veracidad del Paciente

¿La información anterior está significativamente mal representada por:

37. Intención del paciente? 0-No 1-Sí ☐

38. Incapacidad del paciente para comprender? ☐ 0-No 1-Sí

COMENTARIOS SOBRE LA FAMILIA / RELACIONES SOCIALES

(Anote el número de la pregunta con sus notas)

Número de RMA ______________

ESTADO PSIQUIATRICO

¿Cuántas veces en su vida ha sido tratado por cualquier problema psicológico o emocional:

1. En un hospital o como paciente interno? ☐☐

2. Como paciente externo o privado? ☐☐
   >No incluya abuso de sustancias, empleo o consejería familiar. Episodio de tratamiento = serie de más o menos visitas continuas o días de tratamiento, no el número de visitas o días de tratamiento.
   >Anote el diagnóstico en los comentarios si es conocido.

3. ¿Recibe una pensión por incapacidad psiquiátrica? ☐ 0-No 1-Sí

¿Ha tenido períodos significativos (que no hayan sido resultado directo del uso de drogas o alcohol) en que haya:

0-No 1-Sí

	Durante los últimos 30 días	En su vida
4. Tenido depresión seria, tristeza, desesperación, pérdida de interés, dificultad en llevar a cabo las funciones diarias?	☐	☐
5. Sentido ansiedad seria, tensión, preocupación sin razón, incapacidad de sentirse relajado?	☐	☐
6. Tenido alucinaciones (visuales o auditivas)?	☐	☐
7. Tenido dificultad en concentración, retención o memoria?	☐	☐
8. Tenido problemas controlando comportamientos violentos incluyendo episodios de coraje o violencia? >El paciente puede estar debajo de la influencia de alchohol/drogas.	☐	☐
9. Tenido serios pensamientos de suicidio? >El paciente seriamente consideró un plan para quitarse la vida.	☐	☐
10. Intentado suicidio? >Incluye actos de suicidio, gestos o intentos.	☐	☐
11. Sido recetado alguna medicina por algún problema psiquiátrico o emocional? >Recetado al paciente por un médico. Anote "Sí" cuando un medicamento ha sido recetado aunque el paciente no se lo esté tomando.	☐	☐

12. ¿Durante los últimos 30 días, cuántos días ha tenido estos problemas psicológicos o emocionales? ☐☐
   >Esto se refiere a problemas anotados en las preguntas #4 - #10.

Para las preguntas #13 y #14 solicite al paciente que utilize la escala de Evaluación del Paciente.

13. ¿En los últimos 30 días, cuánto le ha preocupado o molestado estos problemas psicológicos o emocionales? ☐
   >El paciente debe de indicar los días problemáticos de la pregunta #12.

14. ¿Cuán importante le es, recibir tratamiento para estos problemas psicológicos o emocionales? ☐

Lo siguiente sera completado por el entrevistador:

Durante la entrevista, el paciente estaba: 0-No 1-Sí

15. Evidentemente deprimido/introvertido ☐

16. Evidentemente hóstil ☐

COMENTARIOS DEL ESTADO PSIQUIATRICO

(Anote el número de la pregunta con sus notas)

Número de RMA ______________

ESTADO PSIQUIATRICO (Continuación)

17. Evidentemente ansioso/nervioso ☐

18. Teniendo problemas de contacto con la realidad, desórdenes de pensamiento, o pensamientos paranoides ☐

19. Teniendo problemas comprendiendo, concentrándose o recordando ☐

20. Teniendo pensamientos de suicidio ☐

Evaluación de Severidad del Entrevistador

21. ¿Cómo evalúa ud. la necesidad del paciente de recibir tratamiento psiquiátrico/psicológico? ☐

Evaluación de Veracidad del Paciente

¿La información anterior está significativamente mal representada por:

22. ¿Intención del paciente? 0-No 1-Sí ☐

23. ¿Incapacidad del paciente para comprender? 0-No 1-Sí ☐

G50. Molidad prevista de tratamiento más apropiada para el paciente:

1. Paciente interno/residencial
2. Paciente externo intensivo (más de seis horas a la semana)
3. Paciente externo
4. Mantenimiento de la metadona
5. No colocan al cliente. El tratamiento no es necesario. ☐

X

G12. Especial Codigo: ☐

1. Entrevistador canceló
2. Paciente rehusó
3. Paciente incapacitado para responder

N. Entrevista completado

COMENTARIOS SOBRE EL ESTADO PSIQUIATRICO (Anote el número de la pregunta con sus notas)

Número de RMA ______________

SUBESCALAS DEL VGNI-I (GAIN I)

VGNI-I

(SOLO PARA LA ENTREVISTA DE ADMISIÓN: NO INCLUYA EL EPISODIO DE TRATAMIENTO ACTUAL.)
Por favor, responda las siguientes preguntas usando el número de episodios.

S7. ¿Cuántas veces en su vida ha sido admitido para recibir tratamiento o consejería por su uso de alcohol o drogas?.. |___|___|
Episodios
[SI "0," PASE A S8]

Tabla de historia detallada de tratamiento
(Si esta es una evaluación autoadministrada, por favor pidale ayuda al personal para completar la siguiente información)

Ahora necesitamos llenar este formulario con todas las veces en que ha estado bajo tratamiento ambulatorio, ambulatorio intensivo, residencial u otras fromas de tratamiento formal por abuso de sustancias, comenzando con el primero hasta el más reciente episodio de tratamiento. Si usted cambió de niveles de cuidado o fue readmitido a tratamiento, por favor cuente cada episodio separadamente. Por favor no incluya programas de desintoxicación, autoayuda o recuperación (los cuales se acaban de reportar).
¿Cuál fue el primer (siguiente ...) programa de tratamiento en el que estuvo? (**Anexe tablas anteriores si están disponibles y actualice**)

**S7** –	b. ¿Cuál es el nombre del programa de tratamiento?	b1. Código del programa	c. ¿Qué tipo de tratamiento era este? (**Use los códigos de la siguiente página**)	d. Aproximadamente, ¿en qué fecha comenzó? (mm/dd/aaaa)	d1. ¿Está aún en tratamiento? (Si es asi, saltese e y g) S N	e. Aproximad-amente, ¿en qué fecha salió? (mm/dd/aaaa)	g. Aproximadamente, ¿cuántos **días** estuvo allí?
1					1 0		
2					1 0		
3					1 0		
4					1 0		
5					1 0		
6					1 0		
7					1 0		
8					1 0		
9					1 0		
10					1 0		
11					1 0		
12					1 0		
13					1 0		
14					1 0		
15					1 0		

99.	**Para uso del personal solamente. Indique número de la fila del índice de tratamiento con la cual la entrevista del VGNI-I actual se relaciona.**	

<table>
<tr><td colspan="2">Resumen de historia del tratamiento e instrucciones y códigos</td></tr>
<tr><td colspan="2">___ Por favor no incluya desintoxicación, grupos de autoayuda, casas de recuperación o santuarios.
___ En el primer renglón comience con la primera admisión y continúe hacia abajo hasta la más reciente.
___ Si el participante está aún bajo tratamiento, deje la fecha cuando se le dio de alta en blanco.
___ Si esta entrevistando de nuevo al participante, por favor anexe y actualice la tabla anterior.
___ Si está en tratamiento, anote 1 o Sí en d1 y saltese items e y g, si no ponga no.
___ Si tiene la fecha de admisión y cuando se le dio de alta (fecha incial y final), saltese la pregunta g (cuánto tiempo).
___ Si le falta la fecha de admisión, estado del tratamiento actual o fecha dado de alta, pregunte por cuanto tiempo y anote la respuesta en días.</td></tr>
<tr><td rowspan="2">Códigos de programas locales comunes (b1) y nombres (b)

(Inserte texto o consulte el apéndice de estudios
específicos)</td><td>Códigos para nivel de atención general (c):
0 Ya no asignado
10 Ambulatorio (“outpatient”)(OP)
15 Mantenimiento con Metadona
20 Ambulatorio intensivo (OR)
30 Internado/Residencial/Casa de Reinserción (media casa, casa de transición, “halfway house”) (ST/LT/HH)
99 Otro</td></tr>
<tr><td>Parámetros para la fecha (d/c):
Use las siguientes reglas si el participante no está seguro de la fecha exacta:
DÍA: Use el 5 para el comienzo del mes, el 15 para la mitad del mes y el 25 para el final del mes.
MES: Use marzo para comienzos de año, julio para mitad de año y octubre para el final de año, pero trate de calcularlo de tal forma que el número de semanas sea correcto.
AÑO: Aproxime basado en la edad u otra información.
Si el participante está aún bajo tratamiento, deje la fecha cuando se le dio de alta</td></tr>
</table>

VGNI-I

**S9. Hoja de trabajo detallada sobre el trastorno del uso de sustancias.**<br>**(Si esta es una evaluación autoadministrada, por favor pidale ayuda al personal para completar la siguiente información)**<br>Para cada uno de los problemas identificados en S9h-u, pregunte:<br>* Puede decirme qué sustancia... (Lea abajo)?<br>* Aproximadamente, ¿cuándo pasó? (Usando la Tarjeta B)<br>* ¿Alguna vez ha tenido este problema con alguna otra sustancia(s)?<br>* Repita para cada Sx en S9 hasta que no haya mas por reportar.<br>Anote el código de tiempo (3= Último mes, 2= Hace 2-12 meses, 1= Hace 1+años, "0" ó en blanco significa nunca)	1	2	3	4	5	6	7	8	9	1[illegible]
	Alcohol	Anfetaminas	Marihuana	Cocaina	Alucinogenooooo	Inhalante	Opioide	PCP	Sed/Ansioliticos	[illegible]
**DSM-IV criterio de abuso**										
h. repetidamente le causó que no cumpliera con sus responsabilidades?										
j. repetidamente usó en situaciones inseguras?										
k. le causó que tenga problemas a cada rato con la ley?										
m. siguió usando sabiendo que lo estaban llevando a peleas o metiéndolo en problemas con otras personas?										
**DSM-IV criterio de dependencia**										
n. necesitó más para sentirse drogado?										
p. tuvo problemas de abstinencia?										
q. usó más o por más tiempo del que realmente quería?										
r. no pudo usar menos o dejar de usar?										
s. pasó mucho tiempo tratando de conseguir o usando?										

t. le causó que dejara actividades o le causó problemas?										
u. siguió usando a pesar de problemas médicos o psicológicos?										
**Importancia clínica (para cada droga cuyo criterio de abuso/dependencia es +1 pregunte…)**										
v. A qué edad usó por primera vez...(Para alcohol, lea "A qué edad se emborrachó por primera vez?"):										
w. Cómo usa normalmente.. . (1-oral, 2-fumando, 3-inhalando, 4-intramuscular, 5-intravenoso, 6- NA, 7-otro)										

Para uso del personal solamente

Vea al manual por como puntuar dependencia poli-sustancia.
NOTA: Para mantener acuerdo, por lo menos uno de los períodos de tiempo se reportó en cada hilera del matrix S9 tiene que ser igual al item correspondiente en S9h-u. Ningún período de tiempo puede ser más temprano que lo que se reportó el item correspondiente en S9h-u.

VGNI- I

SFS/BAC

S2. Tabla de frecuencias sobre el uso de sustancias (Lea de izquierda a derecha con relación a aquellas sustancias que usó en los últimos 90 días)<br>(Si esta es una evaluación autoadministrada,<br>por favor pidale ayuda al personal para completar las siguientes preguntas)	1. Durante los últimos 90 días, ¿Cuántos días ...	2. ¿Cuánto fue lo que más usó (tragos/ joints/etc.) en un día?	3. ¿Durante cuántas horas hizo esto?	4. ¿Con cuántas otras personas (si alguna) estaba compartiendo?
a. usó alguna clase de alcohol?		Tragos		
b. se emborrachó o tomó 5 tragos o más?		X	X	X
c. usó marihuana, hachís, “blunts”<br>(cigarro de marihuana, MX: churro, COL: bareto) o THC?		joints		
d. usó crack, rocas de crack o base (“freebase”)?		rocks		
e. usó otras formas de cocaína (MX: soda)?		quarters		
f. usó inhalantes?		huffs		
g. usó heroína (sola o mezclada)?		dimes		
h. usó metadona de la calle o sin receta?		X	X	X
j. usó calmantes para el dolor, opioides u otros analgésicos?		5v. ¿Qué usó?		
k. usó PCP o polvo de ángel?		X	X	X
m. usó ácido, LSD, ketamina, special K, hongos u otros alucinógenos?		5v. ¿Qué usó?		
n. usó drogas para la ansiedad o tranquilizantes ?		5v. ¿Qué usó?		
pa. usó metanfetamina, cristal, “ice”, “glass” u otras formas de methedrine?			X	
pb. “speed”, “uppers” (reanimadores, activadores), anfetaminas,<br>éxtasis, MDMA u otros estimulantes?		5v. ¿Qué usó?		
q. usó "downers", pastillas para dormir, barbitúricos u otros sedantes?		5v. ¿Qué usó?		
r. usó alguna otra droga?		5v. ¿Qué usó?		

For 5v: Use códigos en S1 o explique con detalle.

Conversiones comunes y normas (0 a 90th percentil de los usuarios)
a.unidades de bebida estándar =1 cerveza=1 copa de vino=1 cóctel=1 trago; 40 onzas de cerveza=4 bebidas; "Fifth"=más de 26
bebidas; (1-20 norma)
c. onza=25-30 " joints"; "dime"=4-5 "joints"; "nicke"l=2-3" joints"; 1"blunt"=2-6" joints"; 1 gramo=1-2" joints"; 1 "bowl"=1"
joint"; 10 1-"hit pipes"= 1" joint";
d. 8" ball"=32 rocas;" teen"=16 rocas; gramo= 10 rocas;" dime"=1 roca;" nickel"= 1 hit= ó roca (redondee al número más
cercano); (1-20 norma)
e. gramo= 4 cuartos de gramo; (5-10 líneas= 1 cuarto de gramo) (1-10 norma)
f. (1-10 norma)
g. (gramo= 10 "dime bags"); (1-10 norma)

VGNI-I

Uso antes de estar en un ambiente controlado
(Si esta es una evaluación autoadministrada, por favor pidale ayuda al personal para completar la siguiente información)

Para ayudarlo a que recuerde el periodo de tiempo para el siguiente grupo de preguntas, saquemos el calendario y como lo hicimos antes señale los últimos 90 días en que estuvo menos de 13 dias en la cárcel, hospital u otro lugar donde no podía usar alcohol, marihuana u otras drogas. ¿Recuerda algo que estuviera ocurriendo alrededor de esa fecha (FECHA HACE 90 DÍAS ANTES DE QUE EL PARTICIPANTE ENTRARA A UN AMBIENTE CONTROLADO)?

(INDAGUE POR UN EVENTO ESPECÍFICO COMO SE HIZO ANTES)

Anote el evento v. ____

Cuando hablamos de cosas que estaban ocurriendo durante los últimos 90 días, estamos hablando de cosas que ocurrieron más o menos desde (NOMBRE DEL EVENTO ANTES DE ESTAR EN UN AMBIENTE CONTROLADO).

S2x. En esos 90 días en la comunidad, ...

	Días
1. ¿cuántos días pasaron sin que usara ninguna clase de alcohol, marihuana u otras drogas?	|__|__|
2. ¿cuántos días se emborrachó o estuvo drogado ("high") casi todo el día?.....	|__|__|
3. ¿cuántos días los problemas por el uso de alcohol u otras drogas no le dejaron cumplir con sus responsabilidades en el trabajo, escuela o casa?	|__|__|
4. ¿cuál fue el mayor número de días seguidos que estuvo sin usar alguna clase de alcohol, marihuana u otras drogas?	|__|__|

[SI NO USÓ EN S2a-r, SALTE LA HILERA RESPECTIVA EN S2ya-r]

S2y. En esos 90 días en la comunidad, ¿cuántos días usó...

a. alguna clase de alcohol?	|__|__|
b. alcohol hasta que se emborrachó (5 o más bebidas de una sola sentada)?......	|__|__|
c. alguna clase de marihuana, hachís, "blunts" (cigarro de marihuana, MX: churro, COL: bareto) u otras formas de THC?	|__|__|
d. alguna clase de crack, roca, pasta o base de cocaína ("freebase")?	|__|__|
e. otras formas de cocaína (MX: soda)?..	|__|__|
f. inhalantes?	|__|__|
g. heroína o heroína mezclada con otras drogas?	|__|__|
h. metadona de la calle o sin receta?...	|__|__|
j. algún calmante para el dolor, opioide u otros analgésicos?............	|__|__|
k. PCP o polvo de ángel?	|__|__|
m. ácido, LSD, ketamina, especial K, hongos u otros alucinógenos?.................	|__|__|
n. alguna droga para la ansiedad o tranquilizantes?..	|__|__|
pa. metanfetamina, cristal, "ice", "glass" u otras formas de methedrine?	|__|__|
pb. "speed", "uppers" (reanimadores, activadores), anfetaminas, éxtasis,	

MDMA u otros estimulantes? .... |__|__|
q. alguna clase de “downers”, “pastillas para dormir”, barbitúricos
u otros sedantes? .... |__|__|
r. alguna otra clase de droga?.. |__|__|
v. ________________________________

Ahora volvemos a los periodos de tiempo originales de los 90 días y los 12 meses para el resto de la entrevista.

En el cuestionario hay una lista de síntomas comunes de la ansiedad. Lea cada uno de los ítems atentamente, e indique cuanto le ha afectado en la última semana incluyendo hoy:

Inventario de Ansiedad de Beck (BAI)

		En absoluto	Levemente	Moderadamente	Severamente
1	Torpe o entumecido.				
2	Acalorado.				
3	Con temblor en las piernas.				
4	Incapaz de relajarse				
5	Con temor a que ocurra lo peor.				
6	Mareado, o que se le va la cabeza.				
7	Con latidos del corazón fuertes y acelerados.				
8	Inestable.				
9	Atemorizado o asustado.				
10	Nervioso.				
		**En absoluto**	**Levemente**	**Moderadamente**	**Severamente**
11	Con sensación de bloqueo.				
12	Con temblores en las manos.				
13	Inquieto, inseguro.				
14	Con miedo a perder el control.				
15	Con sensación de ahogo.				
16	Con temor a morir.				
17	Con miedo.				
18	Con problemas digestivos.				
19	Con desvanecimientos.				
20	Con rubor facial.				
		**En absoluto**	**Levemente**	**Moderadamente**	**Severamente**
21	Con sudores, frios o calientes.				

Inventario de Depresión de Beck.

En este cuestionario aparecen varios grupos de afirmaciones. Por favor, lea con atención cada una. A continuación, señale cuál de las afirmaciones de cada grupo describe mejor cómo se ha sentido durante esta última semana, incluido en el día de hoy. Si dentro de un mismo grupo, hay más de una afirmación que considere aplicable a su caso, márquela también. Asegúrese de leer todas las afirmaciones dentro de cada grupo antes de efectuar la elección, (se puntuará 0-1-2-3).

1) .

- ❑ No me siento triste
- ❑ Me siento triste.
- ❑ Me siento triste continuamente y no puedo dejar de estarlo.
- ❑ Me siento tan triste o tan desgraciado que no puedo soportarlo.

2) .

- ❑ No me siento especialmente desanimado respecto al futuro.
- ❑ Me siento desanimado respecto al futuro.
- ❑ Siento que no tengo que esperar nada.
- ❑ Siento que el futuro es desesperanzador y las cosas no mejorarán.

3) .

- ❑ No me siento fracasado.
- ❑ Creo que he fracasado más que la mayoría de las personas.
- ❑ Cuando miro hacia atrás, sólo veo fracaso tras fracaso.
- ❑ Me siento una persona totalmente fracasada.

4) .

- ❑ Las cosas me satisfacen tanto como antes.
- ❑ No disfruto de las cosas tanto como antes.
- ❑ Ya no obtengo una satisfacción auténtica de las cosas.
- ❑ Estoy insatisfecho o aburrido de todo.

5) .

- ❑ No me siento especialmente culpable.
- ❑ Me siento culpable en bastantes ocasiones.
- ❑ Me siento culpable en la mayoría de las ocasiones.
- ❑ Me siento culpable constantemente.

6) .

- ❑ No creo que esté siendo castigado.
- ❑ Me siento como si fuese a ser castigado.
- ❑ Espero ser castigado.
- ❑ Siento que estoy siendo castigado.

7) .

- ❑ No estoy decepcionado de mí mismo.
- ❑ Estoy decepcionado de mí mismo.
- ❑ Me da vergüenza de mí mismo.
- ❑ Me detesto.

8) .

- ❑ No me considero peor que cualquier otro.
- ❑ Me autocritico por mis debilidades o por mis errores.
- ❑ Continuamente me culpo por mis faltas.
- ❑ Me culpo por todo lo malo que sucede.

9) .

- ❑ No tengo ningún pensamiento de suicidio.
- ❑ A veces pienso en suicidarme, pero no lo cometería.
- ❑ Desearía suicidarme.
- ❑ Me suicidaría si tuviese la oportunidad.

10).

- ❑ No lloro más de lo que solía llorar.
- ❑ Ahora lloro más que antes.
- ❑ Lloro continuamente.
- ❑ Antes era capaz de llorar, pero ahora no puedo, incluso aunque quiera.

11).

- ❑ No estoy más irritado de lo normal en mí.
- ❑ Me molesto o irrito más fácilmente que antes.
- ❑ Me siento irritado continuamente.
- ❑ No me irrito absolutamente nada por las cosas que antes solían irritarme.

12).

- ❑ No he perdido el interés por los demás.
- ❑ Estoy menos interesado en los demás que antes.
- ❑ He perdido la mayor parte de mi interés por los demás.
- ❑ He perdido todo el interés por los demás.

13).

- ❑ Tomo decisiones más o menos como siempre he hecho.
- ❑ Evito tomar decisiones más que antes.
- ❑ Tomar decisiones me resulta mucho más difícil que antes.
- ❑ Ya me es imposible tomar decisiones.

14).

- ❑ No creo tener peor aspecto que antes.
- ❑ Me temo que ahora parezco más viejo o poco atractivo.
- ❑ Creo que se han producido cambios permanentes en mi aspecto que me hacen parecer poco atractivo.
- ❑ Creo que tengo un aspecto horrible.

15).

- ❑ Trabajo igual que antes.
- ❑ Me cuesta un esfuerzo extra comenzar a hacer algo.
- ❑ Tengo que obligarme mucho para hacer algo.
- ❑ No puedo hacer nada en absoluto.

16).

- ❑ Duermo tan bien como siempre.
- ❑ No duermo tan bien como antes.
- ❑ Me despierto una o dos horas antes de lo habitual y me resulta difícil volver a dormir.
- ❑ Me despierto varias horas antes de lo habitual y no puedo volverme a dormir.

17).

- ❑ No me siento más cansado de lo normal.
- ❑ Me canso más fácilmente que antes.
- ❑ Me canso en cuanto hago cualquier cosa.
- ❑ Estoy demasiado cansado para hacer nada.

18).

- ❑ Mi apetito no ha disminuido.
- ❑ No tengo tan buen apetito como antes.
- ❑ Ahora tengo mucho menos apetito.
- ❑ He perdido completamente el apetito.

19).

- ❑ Últimamente he perdido poco peso o no he perdido nada.
- ❑ He perdido más de 2 kilos y medio.
- ❑ He perdido más de 4 kilos.
- ❑ He perdido más de 7 kilos.
- ❑ Estoy a dieta para adelgazar SI/NO.

20).

- ❑ No estoy preocupado por mi salud más de lo normal.
- ❑ Estoy preocupado por problemas físicos como dolores, molestias, malestar de estómago o estreñimiento.
- ❑ Estoy preocupado por mis problemas físicos y me resulta difícil pensar algo más.
- ❑ Estoy tan preocupado por mis problemas físicos que soy incapaz de pensar en cualquier cosa.

21).

- ❑ No he observado ningún cambio reciente en mi interés.
- ❑ Estoy menos interesado por el sexo que antes.
- ❑ Estoy mucho menos interesado por el sexo.
- ❑ He perdido totalmente mi interés por el sexo.

Guía para la interpretación del inventario de la depresión de Beck:

Puntuación	Nivel de depresión*
1-10	Estos altibajos son considerados normales.
11-16	Leve perturbación del estado de ánimo.
17-20	Estados de depresión intermitentes.
21-30	Depresión moderada.
31-40	Depresión grave.
+ 40	Depresión extrema.

* Una puntuación persistente de 17 o más indica que puede necesitar ayuda profesional.

Anexo 3.1 de la publicación "Desarrollo de Programas de Tratamiento Asertivo Comunitario en Andalucía. Documento marco". Servicio Andaluz de Salud, 2010.

ESCALA DE CALIDAD DE VIDA

WHOQOL-BREF

Versión adaptada de:

Murgieri, M. WHOQOL-100 Y WHOQOL-BREF. En red: Última actualización 30/03/2009. http://www.llave.connmed.com.ar/portalnoticias_vernoticia.php?codigonoticia=17621

Torres M, Quezada M; Rioseco R, Ducci ME. Calidad de vida de adultos mayores pobres de viviendas básicas: Estudio comparativo mediante uso de WHOQOL-BREF. Rev Med Chile 2008; 136: 325-333.

Otras referencias de interés:

Lucas R. Versión española del WHOQOL. Madrid: Ediciones Ergon. 1998

Versión original: OMS. The World Health Organization Quality of Life (WHOQOL)-BREF World Health Organization 2004

Antes de empezar con la prueba nos gustaría que contestara unas preguntas generales sobre usted: haga un círculo en la respuesta correcta o conteste en el espacio en blanco.

Sexo: Hombre Mujer

¿Cuándo nació? Día Mes Año

¿Qué estudios tiene? Ninguno Primarios Medios Universitarios

¿Cuál es su estado civil? Soltero/a Separado/a Casado/a

Divorciado/a En pareja Viudo/a

¿En la actualidad, está enfermo/a? Sí No

Si tiene algún problema con su salud, ¿Qué piensa que es? ________________________

____Enfermedad/Problema

Instrucciones: Este cuestionario sirve para conocer su opinión acerca de su calidad de vida, su salud y otras áreas de su vida. Por favor **conteste todas las preguntas**. Si no está seguro/a de qué respuesta dar a una pregunta, escoja la que le parezca más apropiada. A veces, ésta puede ser la primera respuesta que le viene a la cabeza.

Tenga presente su modo de vivir, expectativas, placeres y preocupaciones. Le pedimos que piense en su vida ***durante las dos últimas semanas*** .Por ejemplo, pensando en las dos últimas semanas, se puede preguntar:

		Nada	Un poco	Moderado	Bastante	Totalmente
	¿Obtiene de otras personas el apoyo que necesita?	1	2	3	4	5

Rodee con un círculo el número que mejor defina cuánto apoyo obtuvo de otras personas en las dos últimas semanas. Si piensa que obtuvo bastante apoyo de otras personas, usted debería señalar con un círculo el número 4, quedando la respuesta de la siguiente forma:

		Nada	Un poco	Moderado	Bastante	Totalmente
	¿Obtiene de otras personas el apoyo que necesita?	1	2	3	(4)	5

Recuerde que cualquier número es válido, lo importante es que represente su opinión

Por favor, lea la pregunta, valore sus sentimientos y haga un círculo en el número de la escala que represente mejor su opción de respuesta.

		Muy mala	Regular	Normal	Bastante buena	Muy buena
1	¿Cómo calificaría su calidad de vida?	1	2	3	4	5

		Muy insatisfecho/a	Un poco insatisfecho/a	Lo normal	Bastante satisfecho/a	Muy satisfecho/a
2	¿Cómo de satisfecho/a está con su salud?	1	2	3	4	5

Las siguientes preguntas hacen referencia al grado en que ha experimentado ciertos hechos en las dos últimas semanas.

		Nada	Un poco	Lo normal	Bastante	Extremadamente
3	¿Hasta qué punto piensa que el dolor (físico) le impide hacer lo que necesita?	1	2	3	4	5
4	¿En qué grado necesita de un tratamiento médico para funcionar en su vida diaria?	1	2	3	4	5
5	¿Cuánto disfruta de la vida?	1	2	3	4	5
6	¿Hasta qué punto siente que su vida tiene sentido?	1	2	3	4	5
7	¿Cuál es su capacidad de concentración?	1	2	3	4	5
8	¿Cuánta seguridad siente en su vida diaria?	1	2	3	4	5
9	¿Cómo de saludable es el ambiente físico a su alrededor?	1	2	3	4	5

Las siguientes preguntas hacen referencia a si usted experimenta o fue capaz de hacer ciertas cosas en las dos últimas semanas, y en qué medida.

		Nada	Un poco	Lo normal	Bastante	Totalmente
10	¿Tiene energía suficiente para la vida diaria?	1	2	3	4	5
11	¿Es capaz de aceptar su apariencia física?	1	2	3	4	5
12	¿Tiene suficiente dinero para cubrir sus necesidades?	1	2	3	4	5
13	¿Dispone de la información que necesita para su vida diaria?	1	2	3	4	5
14	¿Hasta qué punto tiene oportunidad de realizar actividades de ocio?	1	2	3	4	5
15	¿Es capaz de desplazarse de un lugar a otro?	1	2	3	4	5

SIGA EN LA PÁGINA SIGUIENTE

Las siguientes preguntas hacen referencia a si en las dos últimas semana ha sentido satisfecho/a y cuánto, en varios aspectos de su vida

		Muy insatisfecho/a	Poco	Lo normal	Bastante satisfecho/a	Muy satisfecho/a
16	¿Cómo de satisfecho/a está con su sueño?	1	2	3	4	5
17	¿Cómo de satisfecho/a está con su habilidad para realizar sus actividades de la vida diaria?	1	2	3	4	5
18	¿Cómo de satisfecho/a está con su capacidad de trabajo?	1	2	3	4	5
19	¿Cómo de satisfecho/a está de sí mismo?	1	2	3	4	5
20	¿Cómo de satisfecho/a está con sus relaciones personales?	1	2	3	4	5
21	¿Cómo de satisfecho/a está con su vida sexual?	1	2	3	4	5
22	¿Cómo de satisfecho/a está con el apoyo que obtiene de sus amigos/as?	1	2	3	4	5
23	¿Cómo de satisfecho/a está de las condiciones del lugar donde vive?	1	2	3	4	5
24	¿Cómo de satisfecho/a está con el acceso que tiene a los servicios sanitarios?	1	2	3	4	5
25	¿Cómo de satisfecho/a está con los servicios de transporte de su zona?	1	2	3	4	5

SIGA EN LA PÁGINA SIGUIENTE

La siguiente pregunta hace referencia a la frecuencia con que usted ha sentido o experimentado ciertos sentimientos en las dos últimas semanas.

		Nunca	Raramente	Moderadamente	Frecuentemente	Siempre
26	¿Con qué frecuencia tiene sentimientos negativos, tales como tristeza, desesperanza, ansiedad, o depresión?	1	2	3	4	5

¿Le ha ayudado alguien a rellenar el cuestionario?

____

¿Cuánto tiempo ha tardado en contestarlo?

____

¿Le gustaría hacer algún comentario sobre el cuestionario?

____

____

____

Gracias por su ayuda

Normas de administración y puntuación

En Badía X, Alonso J. La medida de la salud. Guía de escalas de medición en español. 4ta edición Barcelona: Lilly. 2007

- Concepto: Proporciona un perfil de calidad de vida percibida por la persona. Fue diseñado para ser usado tanto en población general como en pacientes. La WHOQOL-BREF proporciona una manera rápida de puntuar los perfiles de las áreas que explora, sin embargo no permite la evaluación de las facetas individuales de las áreas. Se recomienda utilizarla en epidemiología y en clínica cuando la calidad de vida es de interés, pero no es el objetivo principal del estudio o intervención.

- Existen dos versiones la WHOQOL-100 y la WHOQOL-BREF ambas con estudios de validación en español (que aún continúan completándose). La versión aquí recogida, la WHOQOL-BREF (26 ítems) produce un perfil de 4 dimensiones: Salud física, Salud psicológica. Relaciones sociales, Ambiente.

En Murgieri, M. WHOQOL-100 Y WHOQOL-BREF. En red: Última actualización 30/03/2009. http://www.llave.connmed.com.ar/portalnoticias_vernoticia.php?codigonoticia=17621:

- Administración:

    - El tiempo de referencia que contempla el WHOQOL es de dos semanas.
    - El cuestionario debe ser autoadministrado.
    - Cuando la persona no sea capaz de leer o escribir por razones de educación, cultura o salud puede ser entrevistada.

Freire de Oliveira M, Ábalos Medina GM, Olmedo Alguacil M, Ramírez Rodrigo J, Fernández Pérez AM, Villaverde Gutiérrez C. Estudio comparativo de los instrumentos WHOQOL-BREF Y SF-36, para medir calidad de vida en mayores. Scientia, 2008; 3: En red: http://www.revista-scientia.es/documentos/2008/3.pdf

- Puntuación:

    - El instrumento WHOQOL-BREF ofrece un perfil de calidad de vida, siendo cada dimensión o dominio puntuado de forma independiente. Cuanto mayor

sea es la puntuación en cada dominio, mejor es el perfil de calidad de vida de la persona evaluada.

- Sin embargo no todos los ítems se puntúan de forma directa, hay que recodificar de forma inversa parte de las puntuaciones. Las instrucciones para la recodificación de estos ítems, sobre cómo calcular la puntuación en cada dominio y la estandarización de estas puntuaciones para compararlas con otras escalas se pueden son consulta en: Organización Mundial de la Salud (OMS). WHOQOL-BREF Introduction, administration, scoring and version of the assessment. OMS. Ginebra. 1996: http://www.who.int/mental_health/media/en/76.pdf (ver tablas 3 y 4 en páginas: 12 y 13).

En Bobes García, J; G.-Portilla, MP; Bascarán Fernández, MT, Saiz Martínez; PA, Bousoño García M. Banco de instrumentos básicos para la práctica de la psiquiatría clínica. 3. ª edición. Barcelona: Ars Médica. 2004

- Puntuación:

  - Se obtiene un perfil del paciente y una puntuación sobre percepción de calidad de vida global y salud general. No existen puntos de corte propuestos. A mayor puntuación, mayor calidad de vida.
  - Para su corrección se remite al lector a la versión española del WHOQOL: Lucas Carrasco R. Versión española del WHOQOL. Madrid: Ergón, 1998.

ANEXO 6.9. Escala de espiritualidad WHOQOL-SRPB

Traducción de la sección SRPB de la prueba WHOQOL

Las siguientes preguntas exploran sus **creencias espirituales, religiosas o personales** y cómo estas creencias han afectadosu calidad de vida. Estas preguntas están diseñadas para aplicarse a personas procedentes de diferentes culturas y que sostienen una gran variedad de creencias espirituales, religiosas o personales. Si usted sigue una religión en particular, como el judaísmo, el cristianismo, el islam o el budismo, es probable que responda a las siguientes preguntas con sus referencias religiosas. Si usted no sigue una religión particular, pero aun así todavía cree que existe algo más elevado y más poderoso más allá del mundo físico y material, puede responder a las siguientes preguntas desde esa perspectiva. Por ejemplo, usted podría creer en una fuerza espiritual mayor o el poder curativo de la naturaleza. Alternativamente, puede que usted no crea en una entidad espiritual superior, pero tenga creencias o principios personales fuertes, como la creencia en una teoría científica, una forma personal de vida, una filosofía en particular o un código moral y ético.

Si bien algunas de estas preguntas utilizan palabras tales como espiritualidad, por favor responda en términos de su propio sistema de creencias ya sea religioso, espiritual o personal.

Las siguientes preguntas exploran cómo sus creencias han afectado a diferentes aspectos de su calidad de vida en las últimas dos semanas. Por ejemplo, una pregunta es "¿Hasta qué punto se siente usted conectado con su cuerpo, mente y alma?" Si usted lo ha experimentado mucho, marque el número que se encuentra cerca de "Mucho". Si usted no lo ha experimentado en absoluto, marque el número que se encuentra cerca de "No,en absoluto". Usted deberá marcar uno de los números intermedios si desea indicar que su respuesta se encuentra en algún lugar entre "No, en absoluto" y "Mucho". Las preguntas se refieren a las **últimas dos semanas**.

SP1.1 ¿ En qué medida le ayuda a superar tiempos difíciles tener alguna conexión con un ser espiritual?

Nada en absoluto<br>1	Un poco<br>2	Moderadamente<br>3	Mucho<br>4	Extremadamente<br>5

SPI.2 ¿En qué medida le ayuda a tolerar el estrés alguna conexión con un ser espiritual?

Nada en absoluto<br>1	Un poco<br>2	Moderadamente<br>3	Mucho<br>4	Extremadamente<br>5

SP1.3 ¿En qué medida le ayuda a comprender a los demás alguna conexión con un ser espiritual?

Nada en absoluto 1	Un poco 2	Moderadamente 3	Mucho 4	Extremadamente 5

SP1.4 ¿En qué medida le proporciona consuelo / seguridad alguna conexión con un ser espiritual?

Nada en absoluto 1	Un poco 2	Moderadamente 3	Mucho 4	Extremadamente 5

SP2.1 ¿En qué medida le encuentra sentido a la vida?

Nada en absoluto 1	Un poco 2	Moderadamente 3	Mucho 4	Extremadamente 5

SP2.2 ¿ En qué medida cuidar de otras personas le proporciona sentido a su vida?

Nada en absoluto 1	Un poco 2	Moderadamente 3	Mucho 4	Extremadamente 5

SP2.3 ¿En qué medida siente que su vida tiene un propósito?

Nada en absoluto 1	Un poco 2	Moderadamente 3	Mucho 4	Extremadamente 5

SP2.4 ¿ En qué medida siente que está aquí por alguna razón?

Nada en absoluto 1	Un poco 2	Moderadamente 3	Mucho 4	Extremadamente 5

SP.5.1 ¿En qué medida siente una fuerza espiritual interior?

Nada en absoluto 1	Un poco 2	Moderadamente 3	Mucho 4	Extremadamente 5

SP5.2 ¿En qué medida puede encontrar fuerza espiritual en los momentos difíciles?

Nada en absoluto 1	Un poco 2	Moderadamente 3	Mucho 4	Extremadamente 5

SP8.1 ¿En qué medida la fe contribuye a su bienestar?

Nada en absoluto 1	Un poco 2	Moderadamente 3	Mucho 4	Extremadamente 5

SP8.2 ¿En qué medida la fe le da alivio en la vida diaria?

Nada en absoluto 1	Un poco 2	Moderadamente 3	Mucho 4	Extremadamente 5

SP8.3 ¿En qué medida la fe le da fuerza en la vida diaria?

Nada en absoluto 1	Un poco 2	Moderadamente 3	Mucho 4	Extremadamente 5

SP3.2 ¿En qué medida las cosas bellas de la vida le llenan espiritualmente?

Nada en absoluto 1	Un poco 2	Moderadamente 3	Mucho 4	Extremadamente 5

SP3.3 ¿En qué medida tiene sentimientos de inspiración / entusiasmo en su vida?

Nada en absoluto 1	Un poco 2	Moderadamente 3	Mucho 4	Extremadamente 5

SP3.4 ¿ En qué medida está usted agradecido por las cosas de la naturaleza que puede disfrutar?

Nada en absoluto 1	Un poco 2	Moderadamente 3	Mucho 4	Extremadamente 5

SP7.1 ¿Cuán optimista se siente?

Nada en absoluto 1	Un poco 2	Moderadamente 3	Mucho 4	Extremadamente 5

SP7.2 ¿En qué medida tiene esperanza sobre su vida?

Nada en absoluto 1	Un poco 2	Moderadamente 3	Mucho 4	Extremadamente 5

SP3.1 ¿ En qué medida es capaz de experimentar asombro sobre lo que le rodea? (Por ejemplo, la naturaleza, el arte, la música).

Nada en absoluto 1	Un poco 2	Moderadamente 3	Mucho 4	Extremadamente 5

SP4.1 ¿En qué medida siente alguna conexión entre su mente, su cuerpo y su alma?

Nada en absoluto 1	Un poco 2	Moderadamente 3	Mucho 4	Extremadamente 5

SP4.3 ¿En qué medida siente que la forma en la que vive es consistente con lo que siente y piensa?

Nada en absoluto 1	Un poco 2	Moderadamente 3	Mucho 4	Extremadamente 5

SP4.4 ¿ Cuánto le ayudan sus creencias a crear coherencia entre lo que hace, piensa y siente?

Nada en absoluto 1	Un poco 2	Moderadamente 3	Mucho 4	Extremadamente 5

SP5. 3 ¿Qué tanto la fuerza espiritual le ayuda a vivir mejor?

Nada en absoluto 1	Un poco 2	Moderadamente 3	Mucho 4	Extremadamente 5

SP5.4 ¿En qué medida su fuerza espiritual le ayuda a sentirse feliz en la vida?

Nada en absoluto<br>1	Un poco<br>2	Moderadamente<br>3	Mucho<br>4	Extremadamente<br>5

SP6.1 ¿En qué medida siente paz dentro de sí mismo?

Nada en absoluto<br>1	Un poco<br>2	Moderadamente<br>3	Mucho<br>4	Extremadamente<br>5

SP6.2 ¿En qué medida tiene usted paz interior?

Nada en absoluto<br>1	Un poco<br>2	Moderadamente<br>3	Mucho<br>4	Extremadamente<br>5

SP6.3 ¿ Cuán capaz es usted de sentir paz cuando lo necesita?

Nada en absoluto<br>1	Un poco<br>2	Moderadamente<br>3	Mucho<br>4	Extremadamente<br>5

SP6.4 ¿En qué medida siente una sensación de armonía en su vida?

Nada en absoluto<br>1	Un poco<br>2	Moderadamente<br>3	Mucho<br>4	Extremadamente<br>5

SP7.3 ¿En qué medida ser optimista mejora su calidad de vida?

Nada en absoluto<br>1	Un poco<br>2	Moderadamente<br>3	Mucho<br>4	Extremadamente<br>5

SP7.4 ¿ Cuán capaz es de mantenerse optimista en tiempos de incertidumbre?

Nada en absoluto<br>1	Un poco<br>2	Moderadamente<br>3	Mucho<br>4	Extremadamente<br>5

SP8.4 ¿ En qué medida la fe le ayuda a disfrutar de la vida?

Nada en absoluto<br>1	Un poco<br>2	Moderadamente<br>3	Mucho<br>4	Extremadamente<br>5

SP4.2 ¿Cuán satisfecho se siente de tener un equilibrio entre mente, cuerpo y alma?

Muy insatisfecho<br>1	Insatisfecho<br>2	Ni satisfecho ni insatisfecho<br>3	Satisfecho<br>4	Muy satisfecho<br>5

Anexo 3.3 de la publicación "Desarrollo de Programas de Tratamiento Asertivo Comunitario en Andalucía. Documento marco". Servicio Andaluz de Salud, 2010.

Cuestionario de Satisfacción

Client Satisfaction Questionnaire (CSQ-8)

PERSONAS USUARIAS Y FAMILIARES

Versión adaptada de

Echeburúa E y Corral, P. Manual de violencia familiar. Madrid: Siglo XXI. 1998. Págs: 50-51

Versión original:

Roberts R, Attkisson C. Assessing client satisfaction among Hispanics. Eval Program Plann.1983; (6):401-413.

Otras referencias de interés:

Roberts RE, Attkinsson CC, Mendias RM. Assessing the client satisfaction questionnaire in English and Spanish. Hispanic J Behav Sci 1984; 6(4):385-95.

Por favor para mejorar nuestros servicios nos gustaría hacerle algunas preguntas sobre la atención que usted ha recibido.

Nos interesa conocer su verdadera opinión, sea esta positiva o negativa. Por favor responda a todas las cuestiones que le planteamos. También le agradeceríamos que al final del cuestionario aportase sus comentarios y sugerencias.

Por favor señale primero su tipo de vinculación con el programa

☐ Usuario/a directo/a ☐ Familiar

RODEE CON UN CIRCULO LA OPCIÓN CORRECTA A CADA PREGUNTA

1.- ¿Cómo evaluaría la calidad de los servicios que ha recibido?

4	3	2	1
Excelente	Buena	Regular	Mala

2.- ¿Recibió la clase de servicio que usted requería?

4	3	2	1
No definitivamente	En muy pocos casos	Si en general	Si definitivamente

3.- ¿Hasta que punto ha ayudado nuestro programa a solucionar sus problemas?

4	3	2	1
En casi todos	En la mayor parte	Solo en algunos	En ninguno

4.- ¿Si un/a amigo/a estuviera en necesidad de ayuda similar, le recomendaría nuestro programa?

4	3	2	1
No definitivamente	No , creo que no	Si, creo que si	Si definitivamente

5.- ¿Cómo de satisfecho/a esta usted con la cantidad de ayuda que ha recibido?

4	3	2	1
Nada satisfecho/a	Indiferente o moderadamente no satisfecho/a	Moderadamente satisfecho/a	Muy satisfecho/a

6.- ¿Los servicios que ha recibido le han ayudado a enfrentarse mejor a sus problemas?

4	3	2	1
Si<br>me ayudaron<br>mucho	Si<br>me ayudaron<br>algo	No<br>realmente<br>no me ayudaron	No<br>parecían poner<br>las cosas peor

7.- ¿En general, cómo de satisfecho/a está usted con los servicios que ha recibido?

4	3	2	1
Muy<br>satisfecho/a	Moderadamente<br>satisfecho/a	Algo<br>insatisfecho/a	Muy<br>Insatisfecho/a

8.- ¿Si necesitara ayuda otra vez volvería a nuestro programa?

4	3	2	1
No<br>definitivamente	No<br>posiblemente	Si<br>creo que si	Si<br>con seguridad

Por favor escriba aquí sus comentarios y sugerencias:

Lo que más me ha gustado de la atención que he recibido ha sido:

____

Creo que se tendría que mejorar:

____

MUCHAS GRACIAS POR SU COLABORACIÓN

Normas de administración y puntuación

En: Attkisson CC, Greenfield TK. The UCSF Client Satisfaction Scales: I. The Client Satisfaction Questionnaire-8. In Maruish M (Ed.). The use of psychological testing for treatment planning and outcome assessment. 3ª edición. Mahwah: Lawrence Erlbaum Associates. 2004.

- Administración: Autoaplicado.

  Se recomienda distribuir el cuestionario en el lugar donde se presta la atención o en la sala de espera con una persona designada para solicitar de forma sistemática la participación voluntaria de los y las pacientes o familiares en la muestra. Pueden utilizarse diversos protocolos de toma de muestras: muestra sistemática o aleatoria, estratificada por la duración de los servicios, hasta la fecha, y a todas las personas atendidas durante un período (al menos de dos semanas).

- Puntuación: **Advertencia a profesionales**: Tener en cuenta al aplicar este cuestionario que los ítems 2, 4, 5 y 8 tienen puntuación inversa.

  El cuestionario se basa en 8 preguntas que deben responder los y las pacientes o familiares al final de su estancia en el servicio sobre la base de una escala análogo-visual. Cada pregunta se evalúa entre 1 y 4 puntos y la satisfacción está directamente relacionada con el número de puntos, de modo que la suma de lugar a una variable semicuantitativa que toma valores entre 8 y 32 puntos.

  Se puede calcular (a) la suma no ponderada de los valores corregidos de las respuestas (teniendo en cuenta que los ítems 2, 4, 5 y 8 tendrían puntuación inversa), y (b) medidas de tendencia central (tales como la media, desviación típica, mediana y modo) de los ítems individuales y de la puntuación total.

En: Martínez Azumendi, O y Beitia Fernández, M. Satisfacción, cumplimiento de expectativas y valoración de la ayuda percibida, en primeras consultas en un Centro de Salud Mental. Psiquis, 2000: 21-22

- La versión española parece comportase igual que la inglesa.

- El cuestionario ha servido de comparación para otras escalas de satisfacción más amplias. Las escalas más largas parecen ser más útiles para evaluar aspectos más específicos de los programa de servicios humanos. El CSQ-8 queda como una medida genérica de utilidad.
